# Definitions and operationalizations of pediatric chronic patients: a scoping review

**DOI:** 10.1007/s00431-025-06556-0

**Published:** 2025-11-25

**Authors:** Cor-Jan van der Perk, Lisa-Maria van Klaveren, Karlijn S. Timmer, Heleen N. Haspels, Faridi S. Jamaludin, Lotte Haverman, Willem B. de Vries, Anne M. Eskes, Jolanda M. Maaskant

**Affiliations:** 1 Amsterdam UMC, Emma Children’s Hospital, University of Amsterdam, Meibergdreef 9, 1105 AZ Amsterdam, the Netherlands; 2 Amsterdam Reproduction & Development Research Institute, Amsterdam, the Netherlands; 3 Amsterdam Public Health, Amsterdam, the Netherlands; 4 Institute of Education and Training, Amsterdam UMC, Amsterdam, the Netherlands; 5 Department of Pediatric Intensive, Erasmus Medical Centre, Sophia Children’s Hospital, Rotterdam, the Netherlands; 6 Research Support, Medical Library, Amsterdam UMC, University of Amsterdam, Amsterdam, the Netherlands; 7 Department of Surgery, Amsterdam UMC, Amsterdam, the Netherlands; 8 Department of Internal Medicine, Amsterdam UMC, Amsterdam, the Netherlands

**Keywords:** Pediatric, Definitions, Operationalizations, Chronic conditions, Scoping review

## Abstract

There are significant inconsistencies in the description of different chronic pediatric patient categories, their corresponding definitions and operationalizations. These inconsistencies hamper shared understanding in clinical practice, research and policy-making. To move forward in this field, this scoping review aimed to provide an overview of these definitions and operationalizations. Medline, Embase, PsycINFO, the Cochrane library and CINAHL were searched for studies published between 2019 and 2023, with definitions of patient categories referring to children aged 0–18 years with a chronic condition. Studies selection and data extraction were conducted independently by multiple reviewers. The data synthesis emerged from an iterative process conducted by the research team. Of the 3570 studies, 522 studies contained a definition of a patient category and were included in the review. In 428 studies (82%), 11 definitions were identified, describing eight commonly used patient categories. In 177 (34%) studies, the definition of the pediatric patient category was operationalized. Fifteen different operationalization tools were found. *Conclusion*: Our review reveals the heterogeneous formulation and interchangeable use of definitions of pediatric chronic patients. A minority of studies reported the use of operationalization tools. Achieving consensus on factors that define different patient categories, along with greater uniformity in operationalization tools is needed to enhance the quality and comparability of research, support the quality of clinical care, and enable more efficient use of healthcare resources.

**Table:** 

What is Known: • There is significant heterogeneity in definitions and operationalizations of different chronic pediatric patient categories.	
What is New: • This scoping review provides an overview of the existing definitions and operationalization tools. • Only a minority of studies report on the use of operationalization tools.

**Supplementary Information:**

The online version contains supplementary material available at 10.1007/s00431-025-06556-0.

## Background

The number of children living with one or more chronic condition(s) has increased due to improvements in healthcare and increased treatment options [1]. Approximately 30 to 40% of the pediatric population has at least one chronic condition [1–3]. The complexity of the condition often results in higher healthcare needs and an increase in the number of multidisciplinary healthcare professionals involved [4, 5].

A wide range of terminology is used to describe pediatric chronic patients. For instance, terms such as ‘medical complexity’ [4], ‘special healthcare needs’ [6] and ‘complex chronic conditions’ [7] are frequently used interchangeably, despite varying degrees of complexity. The literature reveals significant inconsistencies in the description of these different chronic patient categories and their corresponding definitions, as well as a lack of clarity in their operationalization [8, 9]. This inconsistency hampers shared understanding in clinical practice, research and policy, potentially leading to inaccurate assessments of care needs of children and their caregivers and reduced care effectiveness. From a research perspective, the absence of standardized definitions and operationalizations compromises the comparability and reproducibility of findings [10, 11]. Therefore, it is crucial to translate existing definitions into measurable and observable operationalizations [11]. Emphasizing this need, van der Lee et al. have highlighted the need for standardized definitions for pediatric chronic patients to support both clinical and research applications [12]. Although efforts have been made to clarify and distinguish different patient categories, substantial variation in terminology, definitions, and operationalizations persists [4, 10].

A clear understanding of the terminology, definitions, and operationalization methods for pediatric chronic patient categories is needed. Therefore, the aim of this scoping review was to systematically map how these patients are categorized and defined, and how definitions are applied in research across all care settings and levels of complexity.

## Methods

We used the methodological framework for scoping reviews [13], and the Preferred Reporting Items for Systematic reviews and Meta-Analyses extension for Scoping Reviews (PRISMA-ScR) checklist [14] for the execution and reporting of the study. As far as the researchers are aware, there is currently no widely accepted prospective registration system specifically for scoping reviews. Therefore, the protocol was not prospectively registered. For the PRISMA-ScR checklist, see Online Resource 1.

### Aims

1. To review definitions of pediatric chronic patients.
2. To examine and summarize how these definitions are operationalized.

### Identifying relevant studies

Prior to conducting this scoping review, the pediatric literature was screened to identify terminology for patient categories frequently used to describe pediatric chronic patients. See: Online Resource 2. These terms were used to build the search strategy. The following databases were systematically searched in consultation with a clinical librarian (FJ): Medline, Embase, PsycINFO, Cochrane Library and CINAHL on 25th of April 2023.

### Inclusion and exclusion criteria

The literature search encompassed studies published between 2019 and 2023 in English, Spanish, German, Dutch, Portuguese, or French. The search strategy is presented in Online Resource 3. Studies were included if they provided definitions to describe pediatric chronic patients aged 0 to 18 years. In this context definitions refer to formal statements specifying the meaning, boundaries, or criteria for classifying patient categories [15]. Eligible study types included quantitative studies, qualitative studies, quality improvement projects, implementation studies, publications describing the development of a definition for a specific pediatric patient category, and reviews.

Study selection was carried out using the web application Rayyan [16]. In this system automatic deduplication took place. Two independent reviewers (CJP, JM) screened independently the first 500 titles and abstracts to ensure consistency in the selection procedure. Based on this initial screening and discussions within the research team, the in- and exclusion criteria were refined and consequently used for the remaining articles.

Studies were excluded when they reported on specific diagnoses (e.g., asthma) or focused on the transition from pediatric to adult care. The latter were excluded because, although we believe that the transition to adult care is a very critical phase, it is also a distinct one that may require different conceptualizations. Excluded publication types were errata, letters to the editor, posters and conference abstracts, narratives, educational programs for healthcare professionals, case reports, expert opinion publications, and clinimetric studies.

### Study selection

All titles and abstracts were screened by two reviewers independently (CJP, LK). Discrepancies were discussed and resolved. Subsequently, the studies potentially eligible for inclusion were independently divided among the research team for full text selection. Full-text selection was performed by a single reviewer (CJP, JM, KT, LK). Each researcher reviewed 250–300 studies. Discrepancies and doubts in the process of screening were discussed in the research team until consensus was reached.

### Charting the data

A charting table was developed and independently tested on 20 randomly selected studies by two authors (CJP, JM). Subsequently, the remaining articles were divided among four researchers for data extraction (CJP, JM, KT, LK). Extracted data included study characteristics, the patient categories used to describe the study populations, their definitions, and methods of operationalization. In this context, operationalization refers to the process of turning concepts or criteria into measurable and observable terms [17]. To ensure consistency in the approach, data charting was checked in 10% of randomly selected studies. No notable differences were found.

### Collating, summarizing and reporting results

The definitions describing the patient categories as used in the included publications were initially summarized by the first author (CJP). Subsequently, another researcher (JM) further summarized these definitions by identifying similarities and differences in key conceptual elements across the categories. A manual, inductive approach was applied. We considered the following key conceptual elements: healthcare needs, specialized care needs, family needs, healthcare utilisation, medical health condition, duration of condition, and functional limitation. Two additional researchers (LK, KT) independently verified the consistency of this synthesis with the original definitions. The tools used to operationalize the definitions, along with their main characteristics, were also summarized by the first author. Doubts and results were discussed within the research team until consensus was reached. To support interpretation, two researchers (CJP, LK) systematically compared the textual components of the definitions and visualized their interrelationships. These visualizations were iteratively reviewed and refined by two other researchers (JM, AE) before finalization.

### Critical quality appraisal

In accordance with the guideline for scoping reviews, the quality of the included studies was not critically appraised.

### Ethical considerations

Ethical approval of this study was not required as per Medical Research Involving Human Subjects Act.

## Results

### Identification and characteristics of included studies

**Fig. 1:**
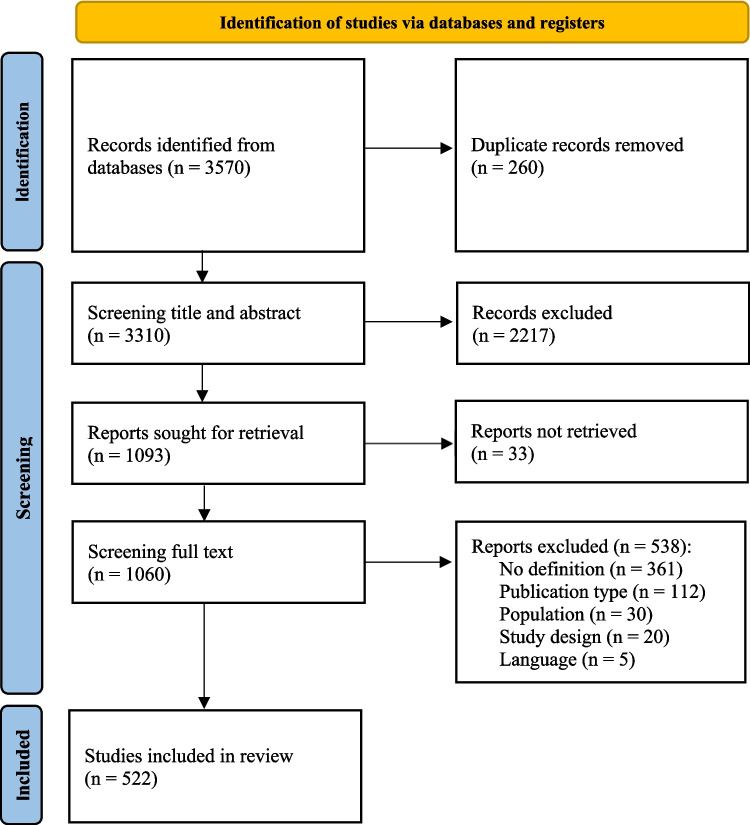
PRISMA Flow diagram of the scoping review process

The search strategies yielded a total of 3570 records. After deduplication and screening of title and abstracts, 1060 publications were identified as potentially relevant. During the full-text screening, 538 reports were excluded for the following reasons: no definition, publication type, population, study design, and language. We finally included 522 studies. The selection process is visualized in Fig. 1. Most of the included studies originated from the USA (55%) and Europe (23%). We identified a variety of study designs, such as quantitative studies (42%), qualitative studies (26%), and reviews (16%). A summary of all study characteristics is represented in Table 1.

**Table 1:** Characteristics of included studies (*n* = 522)

	***n***	**%**
**Country of origin**		
USA	287	55%
Europe	118	23%
Canada	56	11%
South America	24	5%
Asia	23	4%
Australia	12	2%
Africa	2	< 1%
**Study type**		
Quantitative study	217	42%
Qualitative study	137	26%
Review	82	16%
Quality improvement study	33	6%
Mixed methods study	19	4%
Definition study	13	2%
Implementation study	10	2%
Other	11	2%
**Study participants**		
Parents or caregivers	204	39%
Pediatric patients	187	36%
Healthcare professionals	22	4%
Siblings	4	1%
School professionals	3	1%
Managers	3	1%
Combinations	91	17%
Not registered	8	2%

### Data synthesis

The references of the included publications are presented in Online resources 4 and 5 to improve the readability of the manuscript.

#### Definitions

In 428 studies (428/522, 82%), we identified 11 definitions describing eight patient categories: (a) children with medical complexity (CMC), (b) children with complex chronic conditions (CCC) (2 definitions), (c) children (and youth) with special healthcare needs (C(Y)SHCN), (d) children with life-limiting conditions (LLC) (2 definitions), (e) children with profound and multiple intellectual disabilities (PMID), (f) pediatric chronic condition (PCC), (g) children with technology dependence (TD), (h) pediatric chronic critical illness (PCCI) (2 definitions). An overview is provided in Table 2. In 37 studies (7%), other patient categories were used to describe the population, however the corresponding definitions showed considerable overlap in wording with the 11 most commonly used definitions. The definitions in the remaining 94 studies (18%) were considerably different. Further elaboration can be found in the subsequent sections of this paragraph.

**Table 2:** Definitions per patient category

Patient category	Definition	Author
CMC	Children with multiple chronic conditions, severe functional limitations, substantial family care needs, and high healthcare use	(Cohen et al., 2011)
C(Y)SHCN	Children (or youth) who have a chronic condition or are at an increased risk for a chronic condition, including physical, developmental, behavioral, or emotional conditions, and require health and health-related services of a type or amount beyond which is required by children generally	(McPherson et al., 1998)
CCC	Children with any medical condition that can be reasonably expected to last at least 12 months (unless death intervenes) and to involve either several different organ systems or 1 organ system severely enough to require specialty pediatric care and probably some period of hospitalization in a tertiary care center	(Feudtner et al., 2000)
	Any physical, mental, or developmental condition that can be expected to last at least a year, using healthcare resources above the level for a healthy child, requiring treatment to control. This can be expected to be episodically or continuously debilitating; a progressive condition that is associated with deteriorating health with a decreased life expectancy in adulthood	(Simon et al., 2010)
LLC	Children for which there is no reasonable hope of cure and from which children or young people will ultimately die prematurely	(Fraser et al., 2012)
	Life-limiting conditions are typically defined as either (1) life-threatening conditions for which curative treatment may be feasible but can fail, (2) conditions where premature death is in evitable, (3) progressive conditions without curative options or (4) irreversible but non-progressive conditions causing severe disability, leading to susceptibility to health	(Chambers, 2018)
PMID	Persons with PMID have an estimated IQ below 20. They have profound neuromotor dysfunctions, often accompanied by sensory impairments and medical problems, such as seizures, respiratory and feeding problems. Persons with PMID have little or no understanding of verbal language and no apparent symbolic interaction with objects and are therefore always dependent on others	(Nakken & Vlaskamp, 2002)
PCC	A disease or a condition is considered to be a pediatric chronic condition if: (1) it occurs in children aged 0 up to 18 years, (2) the diagnosis is based on medical scientific knowledge and can be established using reproducible and valid methods or instruments according to professional standards, (3) it is not (yet) curable or, for mental health conditions, if it is highly resistant to treatment, and (4) it has been present for longer than 3 months or it will be, very probably, last longer than 3 months, or it has occurred three times or more during the past years and will probably reoccur	(Mokkink et al., 2008)
TD	Children dependent on a medical device to compensate for the loss of a vital bodily function and substantial and ongoing nursing care to avert death or further disability	(United States Congress Office of Technology Assessment, 1987)
PCCI	Children with complex and chronic medical conditions who require recurrent and prolonged Intensive Care Unit hospitalizations and require medical technology for the support of vital functions	(Shapiro et al., 2017)
	Pediatric patients staying in the intensive care unit for at least 14 days and having at least one additional criterion, including prolonged mechanical ventilation, tracheostomy, sepsis, severe wound (burn) or trauma, encephalopathy, traumatic brain injury, status epilepticus, postoperative (cardiac and non-cardiac), and neuromuscular disease	(Demirkiran et al., 2021)

*CMC* Children with medical complexity, *C(Y)SHCN* Children (and Youth) with Special Healthcare Needs, *CCC* Complex Chronic Conditions, *LLC* Life Limiting Conditions, *PMID* Profound and Multiple Intellectual Disabilities, *PCC* Pediatric Chronic Condition, *TD* Technology Dependence, *PCCI* Pediatric Chronic Critical Illness

- Children with Medical Complexity (CMC)
  In 187 studies (187/522), the definition proposed by Cohen et al. was used: *“Children with multiple chronic conditions, severe functional limitations, substantial family care needs, and high health service use”* [4]. Most studies (176/187) named their population CMC. Eleven studies (11/187) used different terminology to describe the patient category while using Cohen’s definition, e.g. medically complex children, infants or neonates with medical complexity. Ten studies provided additional information to the definition, such as referring to the high risk of mortality and morbidity, and the need for multidisciplinary highly specialized care. For corresponding references, see Online Resource 4A.
- Children (and Youth) with Special Health Care Needs (C(Y)SHCN)
  In 100 studies (100/522), the definition of McPherson et al. was used: *“Children who have a chronic condition or are at an increased risk for a chronic condition, including physical, developmental, behavioral, or emotional conditions, and require health and health-related services of a type or amount beyond which is required by children generally”* [6].
  Most studies (88/100) used the term C(Y)SHCN to describe their patient population. Twelve studies (12/100) used different wordings to describe the patient category while using McPherson’s definition, e.g. pediatric complex chronic care patients or children with complex care needs. For corresponding references, see Online Resource 4B.
- Children with Complex Chronic Conditions (CCC)
  We found 45 studies (45/522) providing two different definitions of CCC. In 43 studies, the definition of Feudtner et al. was used: *“Children with any medical condition that can be reasonably expected to last at least 12 months (unless death intervenes) and to involve either several different organ systems or 1 organ system severely enough to require specialty pediatric care and probably some period of hospitalization in a tertiary care centre”* [18]. Most studies (37/43) named their population CCC. Six studies (6/43) used different terminology to describe the patient category while using Feudtner’s definition, e.g. children with complex health conditions, children with life-limiting complex chronic conditions, pediatric home healthcare users, or children with multiple complex chronic conditions. Three studies provided additional information to the definition, e.g. referring to the higher risk of mortality compared to children with other health conditions.
  Two studies (2/45) used the definition by Simon et al.: *“Any physical, mental, or developmental condition that can be expected to last at least a year, using healthcare resources above the level for a healthy child, requiring treatment to control. This can be expected to be episodically or continuously debilitating; a progressive condition that is associated with deteriorating health with a decreased life expectancy in adulthood”* [19].
  For corresponding references, see Online Resource 4C.
- Children with Life-Limiting Conditions (LLC)
  We found 32 studies (32/522) providing two different definitions of LLC. In 16 studies, the definition of Fraser et al. was used: *“Children for which there is no reasonable hope of cure and from which children or young people will ultimately die prematurely, e.g. Duchenne muscular dystrophy or neurodegenerative disease”* [20].
  A second definition as described by the charity ‘Together for Short Lives’ was used in the other 16 studies: *“Life-limiting conditions are typically defined as either (1) life-threatening conditions for which curative treatment may be feasible but can fail, (2) conditions where premature death is inevitable, (3) progressive conditions without curative options or (4) irreversible but non-progressive conditions causing severe disability, leading to susceptibility to health”* [21]. Two studies slightly modified the Together for Short Lives definition referring to non-malignant LLC in childhood. For corresponding references, see Online Resource 4D.
- Children with Profound and Multiple Intellectual Disabilities (PMID)
  In 24 studies (24/522), the definition of Nakken and Vlaskamp was used: *“Persons with PMID have an estimated IQ below 20. They have profound neuromotor dysfunctions, often accompanied by sensory impairments and medical problems, such as seizures, respiratory and feeding problems. Persons with PMID have little or no understanding of verbal language and no apparent symbolic interaction with objects and are therefore always dependent on others.”* [22]. Most studies (19/24) named their population PMID. Five studies (5/24) used different terminology to describe the patient category while using Nakken and Vlaskamp’s definition, e.g. children with significant cognitive and motor developmental delays, or children with poly-handicap. For corresponding references, see Online Resource 4E.
- Pediatric chronic conditions (PCC)
  In 21 studies (21/522), the definition of Mokkink et al. was used: *“A disease or a condition is considered to be a chronic condition in childhood if: (1) it occurs in children aged 0 up to 18 years, (2) the diagnosis is based on medical scientific knowledge and can be established using reproducible and valid methods or instruments according to professional standards, (3) it is not (yet) curable or, for mental health conditions, if it is highly resistant to treatment, and (4) it has been present for longer than 3 months or it will be, very probably, last longer than 3 months, or it has occurred three times or more during the past years and will probably reoccur.”* [23]. Seven studies provided additional information to the definition, such as referring to daily life limitations, psychological problems, and a duration of the disease of at least 12 months. For corresponding references, see Online Resource 4F.
- Children with technology dependence (TD)
  In 10 studies (10/522), the definition as described by the United States Congress Office of Technology Assessment (OTA) was used: *“Children with a medical device to compensate for the loss of a vital bodily function and substantial and ongoing nursing care to avert death or further disability”* [24]. Most studies (7/10) named their population TD. Three studies used different terminology to describe the patient category while using OTA’s definition, i.e. medically complex technology-dependent children, children dependent on respiratory support, or children with invasive long-term ventilator dependence. For corresponding references, see Online Resource 4G.
- Pediatric Chronic Critical Illness (PCCI)
  We found 9 studies (9/522) on PCCI providing two different definitions. In eight studies the definition of Shapiro et al. was used*: “Children with complex and chronic medical conditions who require recurrent and prolonged ICU hospitalizations and require medical technology for the support of vital functions”* [25]. One study provided additional information to this definition, referring to multi-organ dysfunction and a long stay at a Pediatric Intensive Care Unit. One study used the definition of Demirkiran et al.: *“Pediatric patients staying in the intensive care unit for at least 14 days and having at least one additional criterion, including prolonged mechanical ventilation, tracheostomy, sepsis, severe wound (burn) or trauma, encephalopathy, traumatic brain injury, status epilepticus, postoperative (cardiac and non-cardiac), and neuromuscular disease”* [26]. For corresponding references, see Online Resource 4H.

### Other findings

We identified 94 publications (94/522, 18%) that could not be classified under any of the aforementioned definitions or patient categories. Eighteen studies did not correspond to one of the patient categories. However, these standalone patient categories frequently incorporated criteria from existing definitions or constructed new definitions by combining elements of the existing definitions. Particularly, in 21 studies the patient category PCC showed a considerable variation in definitions. The time indication was a recurrent topic, as well as additional information such as medical care, daily life limitations, quality of life, and psychological problems. In 36 studies a definition was used to describe another patient category. Finally, we found 19 studies, that used operationalization tools to define the patient categories, e.g. the CSHCN screener [27], National Survey of Children’s Health [28] and the Chronic Condition Indicator [29] were used to define C(Y)SHCN (online resource 6). For corresponding references, see Online Resource 4I.

### Similarities and relations between the definitions

**Fig. 2:**
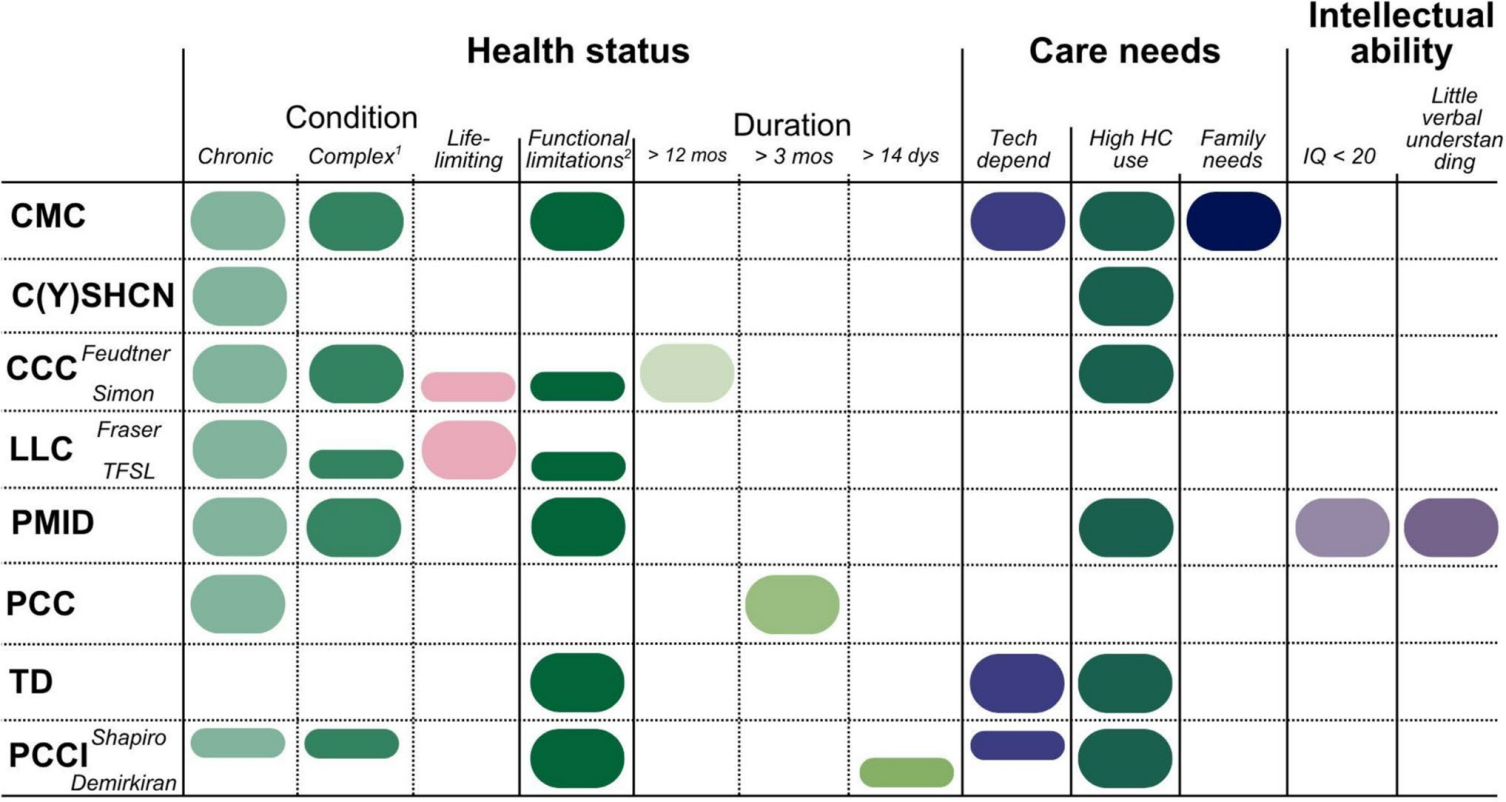
Overview of textual aspects of definitions. Abbreviations: *CMC* Children with Medical Complexity; *CYSHCN* Children and Youth with Special Healthcare Needs; *CCC *Children with Chronic Complex Conditions; *LLC* Children with Life Limiting Conditions; *PMID* Profound and Multiple Intellectual Disabilities; *PCC* Pediatric Chronic Condition; *TD* Children with Technology Dependence; *PCCI* Pediatric Chronic Critical Illness

All definitions included a description of the health status of children, with some definitions incorporating the care needs, and intellectual ability of the children. See Fig. 2.

**Fig. 3:**
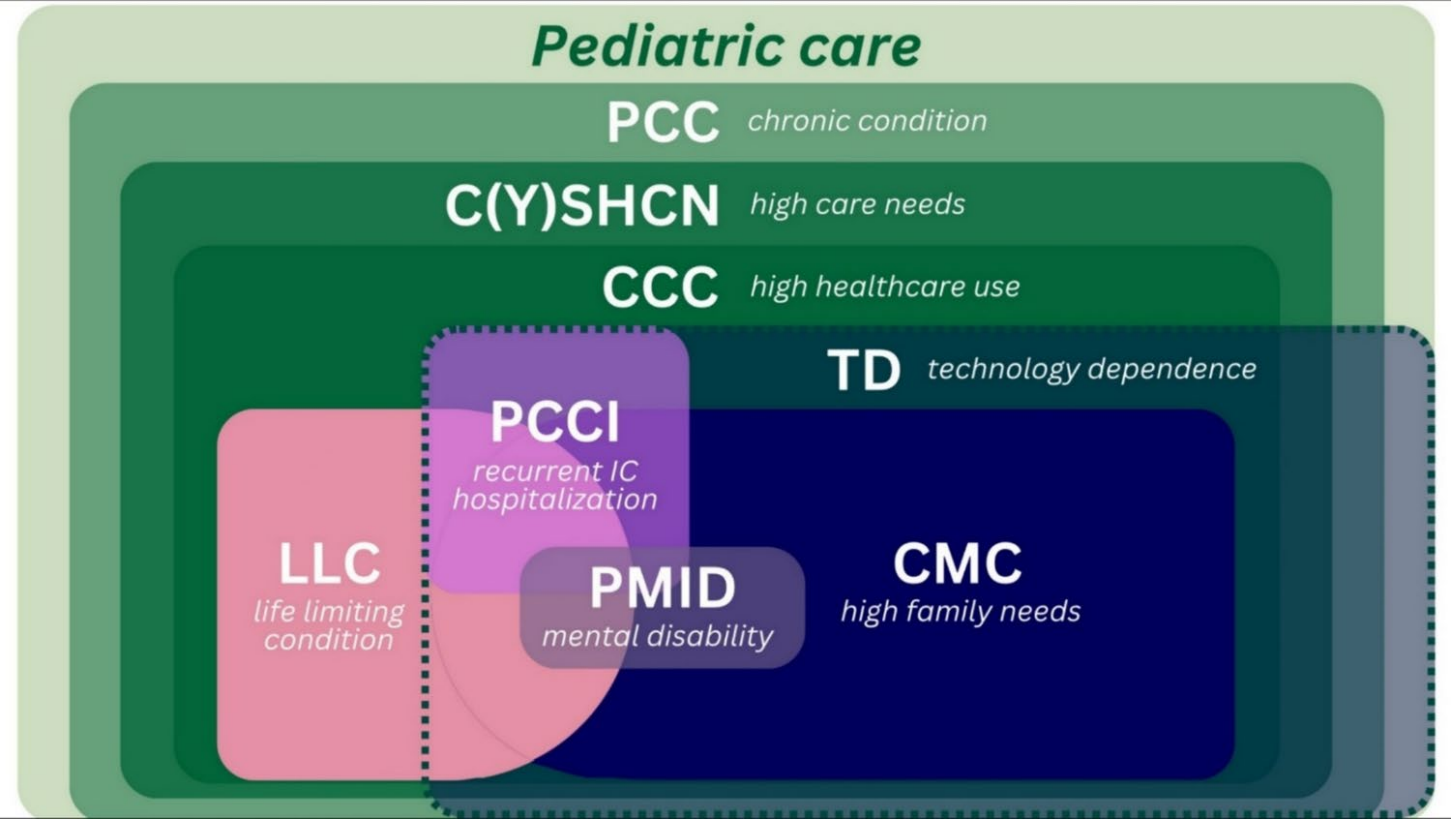
Nested structure of patient category definitions. Abbreviations: *PCC* Pediatric Chronic Condition; *CYSHCN* Children and Youth with Special Healthcare Needs; *CCC* Children with Chronic Complex Conditions; *TD* Children with Technology Dependence; *CMC* Children with Medical Complexity; *PCCI* Pediatric Chronic Critical Illness; *PMID* Profound and Multiple Intellectual Disabilities; *LLC* Children with Life Limiting Conditions

Based on shared textual components, the outlined definitions seem to be nested, with one patient category representing a subpopulation of another. For example, CSHCN can be considered a subpopulation of PCC, while CCC and CMC can be seen as subpopulations of CSHCN. See Fig. 3.

#### Operationalizations

In total 177 (177/522, 34%), studies provided an operationalization of the definition used to describe the patient category. In 105 studies (105/177, 59%) an operationalisation tool was used, and in 72 studies (72/177, 41%) the operationalization was practice or opinion-based.

We found 15 different operationalization tools: (1) CSHCN screener [27], (2) Complex Chronic Condition Classification System (CCCS) [30], (3) National Survey Children’s Health (NSCH) [28], (4) Pediatric Medical Complexity Algorithm (PMCA) [31], (5) the International statistical Classification of Diseases and related health problems (ICD) [18], (6) Standard Operational Definition for Children with Medical Complexity [32], (7) Gross Motor Function Classification System (GMFCS) [33], (8) 3 M Clinical Risk Group classification (CRG) [34], (9) Chronic Condition Indicator (CCI) [29], (10) Children With Disabilities Algorithm (CWDA) [35], (11) Office of Technology assessment (OTA) criteria [24], (12) Assessment of Complex Clinical Assistance Needs in Pediatrics (ACCAPED) [36], (13) Adjusted Morbidity Groups classification system (AMG) [37], (14) Parent of a Child with Medical Complexity Eligibility Questionnaire [38] and (15) Bob’s Level of Support Scale (BLSS) [39].

Five of the 15 tools used the ICD codes to determine different patient categories: PMCA [31], CWDA [35], CCCS [18], CRG [34], and the CCI [29]. Several tools were used to operationalize one specific patient category. For instance, the Gross Motor Function Classification System was only used to operationalize PMID. In contrast, we also found that several tools were used across patient categories, e.g. the CSHCN screener was not only used to specify the definition of CSHCN, but also used to operationalize CMC and PCC. An overview of all identified tools is provided in Table 3. For corresponding references, see Online Resource 5.

**Table 3:** Tools used to operationalize the different patient categories with pediatric chronicity

Tool	ICD	Source	Description of method	Total studies *n*/Studies per patient category (*n*)
CSHCN screener	No	Maternal and Child Health Bureau of the Health Resources and Services Administration, USA	The CSHCN Screener identifies children with a range of chronic conditions and special needs It is a five-item, parent-reported tool. The first part of each CSHCN Screener question asks whether a child experiences one of five different health consequences (1) use of prescription medication, (2) above average use of medical, mental health or educational services, (3) functional limitations, (4) the need of specialized therapies, or (5) treatment or counseling for emotional or developmental problems The second and third sections of each screening question are directed at respondents who answered “yes” to the first part. These sections inquire if the outcome is caused by a health condition and, if so, whether that condition has persisted or is expected to persist for at least 12 months. To meet the criteria of the CSHCN screener, all three parts of at least one screening question (or both parts in the case of question 5) must be answered with “yes.” (Bethell et al., 2002) Kuo et al. (2014) developed cut-off values to use the CSHCN screener to identify CMC, being: “a need for medical care, evidenced by a positive response to the medical care question on the Children with Special Health Care Needs Screener; multiple needs across different domains, as evidenced by a positive response to at least three of the remaining four screener questions; and having seen at least two specialists in the previous year.” (Kuo et al., 2014)	*n* = 25/CSHCN (*n* = 17), CMC (*n* = 7), PCC (*n* = 1)
Complex Chronic Condition Classification System (CCCS)	Yes	Children’s Hospital of Philadelphia and the University of Pennsylvania, USA	The CCCS are groupings of the ICD codes that identifies patients according to the definition of CCC (Feudtner et al., 2000). The algorithm can be used to identify children with a specific CCC category or to identify patients with multiple CCC categories, including: Cardiovascular, Neurologic or neuromuscular, Malignancy, Congenital or genetic, Respiratory, Neonatal, Hematologic or Immunologic, Renal, Metabolic, Gastrointestinal, Transplant. If a patient has any of these categories identified, then ICD procedure codes are examined to determine if the patient has technology dependency in any of the categories (Feudtner et al., 2000; Feudtner et al., 2014)	*n* = 29/CCC (*n* = 21), CMC (*n* = 7), TD (*n* = 1)
National Survey Children's Health (NSCH)	No	Maternal and Child Health Bureau of the Health Resources and Services Administration, USA	The NSCH is a household survey that generates national and state-level data on the physical and emotional health of children aged 0–17 years in the United States. It consists of a Screener Questionnaire (2 topics with a minimum of 15 questions) and a Topical Questionnaire (11 topics with 179 questions) on children's health and well-being, encompassing access to and use of health care, family interactions, parental health, school and after-school experiences, and neighborhood characteristics. The NSCH was also developed to assess the prevalence and situation of children with special health care needs (CSHCN) in the USA (van Dyck et al., 2004)	*n* = 17/CMC (*n* = 5), CSHCN (*n* = 10), CCHC (*n* = 1), PCC (*n* = 1)
Pediatric medical Complexity Algorithm (PMCA)	Yes	Washington State Medicaid, USA	The PMCA was designed to stratify children based on their medical complexity. This algorithm categorizes children into three levels of chronic disease suing ICDS codes**: (1) complex chronic disease (C-CD)** operationalized as: Significant physical, mental or developmental chronic conditions in two or more body systems, expected to last at least a year, will use health care resources above the level for a healthy child, require treatment for control of the condition OR a progressive, deteriorating condition with a decreased life expectancy in adulthood. OR continuous dependence on technology for at least six months. OR Malignancies: Progressive or metastatic malignancies that impact life function, (2) **noncomplex chronic disease (NC-CD)** operationalized as: Chronic Conditions that last at least one year, involve a single body system are commonly lifelong and are non-progressive., and (3) **without chronic disease (CD)** operationalized as a physical, developmental or mental health condition that is not expected to last more than a year, using temporarily (for < 1 year health care resources above the normal level for a healthy child (Simon et al., 2014)	*n* = 11/CMC (*n* = 11)
International Classification of Diseases (ICD)	Yes	World Health Organisation (WHO)	The ICD is a global medical classification system that contains 12,000 hierarchically structured concepts of diseases, signs, symptoms, abnormal findings, patient complaints, social circumstances, and external causes of injury or diseases. The ICD maps health conditions to corresponding categories with specific variations, assigning designated codes(World Health Organisation, 2005)	*n* = 6/CMC (*n* = 1), TD (*n* = 2), PCC (*n* = 2), LLC (*n* = 1)
Children with Disabilities Algorithm (CWDA)	Yes	Center of Excellence for Pediatric Quality Measurement (CEPQM), USA	CWDA identifies children with disabilities (CWD) based on ICD-9 codes. The algorithm helps identify CWD to evaluate care quality and experiences, and compare it to care provided to children without disabilities. The CWDA includes 669 ICD-9-CM codes associated with disabilities in children, in both inpatient and outpatient datasets (Chien et al., 2015)	In one study combined with PMCA
Chronic Condition Indicator (CCI)	Yes	Hwang et al., 2001	The CCI is used to classify ICD-9-CM diagnosis codes into two categories: chronic or non-chronic. Chronic conditions include cancer, diabetes, mental illness, and heart disease, while non-chronic conditions include infections, pregnancy, and injuries. This tool uses around 14,000 diagnosis codes from the ICD (Hwang et al., 2001)	In one study combined with PMCA, in one study combined with CRG
The standard Operational Definition for Children with Medical Complexity (SODCMC)	No	Provincial Council for Maternal and Child Health (PCMCH), Ontario, Canada	The SODCMC was developed as a decision tool to decide whether a child is eligible for Complex Care for Kids of Ontario(CCKO) program. The SODCMC has five operationalization categories: Technology dependent and/or users of high intensity care (e.g. dependent on mechanical ventilators, child has any chronic condition that requires great level of care), fragility (e.g. severe and/or life-threatening condition),, chronicity (condition is expected to last at least six more months), complexity (involvement of at least five healthcare practitioners, family circumstances impede their ability to provide day-to-day car), and geography(significant challenges to seek appropriate rural medical services). For eligibility the child must be under 18 years of age and meets one criterion from four of the five categories("Complex Care for Kids Ontario (CCKO), 2016)	*n* = 5/CMC (*n* = 5)
Gross Motor Function Classification System (GMFCS)	No	Neurodevelopmental Clinical Research Unit, McMaster University, Canada	The GMFCS classifies the gross motor function of children and youth with cerebral palsy, focusing on self-initiated movements such as sitting, walking, and wheeled mobility. It is a five-level system based on functional abilities and the need for assistive devices. It emphasizes current performance in daily settings (home, school, community) rather than maximum potential. Children at Level I walk without restrictions but struggle with advanced motor skills, while those at Level V have severe mobility limitations even with assistive technology (Rosenbaum et al., 2008)	*n* = 3/PMID (*n* = 3)
3 M Clinical Risk Group classification (CRG)	Yes	3 M Health Information Systems, USA	CRG is used for tracking chronic disease rates, profiling healthcare usage, adjusting risk for payment models, and linking patient satisfaction with care quality CRG classifies individuals in 9 health status groups based on their clinical and demographic characteristics, using data from claims systems, electronic patient records and other healthcare data. Individuals without chronic conditions are grouped as healthy or assigned to a significant acute group if they have a history of serious acute conditions (Hughes et al., 2004)	*n* = 2/MCC (*n* = 2)
Office of Technology Assessment (OTA) criteria	No	United States Congress’ Office of Technology Assessment, USA	The OTA assesses four groups of children dependent on technology due to their clinical needs: Group I: Children dependent on mechanical ventilators for part of each day Group II: Children requiring prolonged intravenous administration of nutritional substances or drugs Group III: Children needing daily respiratory or nutritional support via devices like tracheotomy tubes, suctioning, oxygen, or tube feeding Group IV: Children dependent on other medical devices compensating for vital body functions, needing daily or near-daily nursing care, including those on apnea monitors, renal dialysis, or using urinary catheters or colostomy bags Groups I-III involve life-threatening dependencies with frequent, complex nursing tasks. Group IV has less frequent or complex nursing needs, reducing the risk of long-term hospitalization (OTA, 1987)	*n* = 2/TD (*n* = 2)
The Assessment of Complex Clinical Assistance Needs in Pediatrics (ACCAPED)	No	Developed by a multidisciplinary group of experts in Pediatric palliative care (PPC), Italy	The ACCAPED Scale is questionnaire for assessing the clinical needs of children with life-limiting or life-threatening illnesses. It evaluates 10 domains of pediatric palliative care (PPC) needs, including breathing, feeding, mobility, and pain. Each domain is scored from 1 to 50 based on complexity. A "surprise question" is also included to assess the likelihood of survival within 12 months, adding up to 50 points. Total scores guide referrals: low-complexity (≤ 29) to community care, moderate (30–49) to general PPC, and high-complexity (≥ 50) to specialized PPC services (Lazzarin et al., 2021)	*n* = 1/CMC (*n* = 1)
the Adjusted Morbidity Groups classification system (AMGC)	No	the Catalan Institute of Health (ICS), Spain	The AMGC is a morbidity measurement specifically developed for the Spanish healthcare system for primary care. It stratifies the population into six morbidity groups, each further divided into five complexity levels, plus one healthy group, resulting in 31 categories (Monterde et al., 2016)	*n* = 1/CMC (*n* = 1)
Parent of a Child with Medical Complexity Eligibility Questionnaire	No	Buchanan et al (2022)	Parent of a Child with Medical Complexity Eligibility Questionnaire is parental questionnaire to determine whether a child meets the criteria for CMC. It contains six questions. If parents answer “yes” to at least the 2 questions for medical complexity the child or the parent is included in the study. (1) Are you currently a caregiver to a child with one or more disabilities? (2) Does the child in your care meet all of the following criteria: Has one or more chronic condition(s), either diagnosed or unknown. AND Requires frequent and/or prolonged visits to the hospital, pediatrician, or therapist or the ongoing involvement of multiple subspecialty services and providers. AND Is reliant on medical technology, assistive support person(s), or equipment to support daily activities (feeding tube, ventilator, bi-pap, tracheostomy, wheelchair, etc.) (3) Has the child in your care received care at a local children’s hospital, children’s treatment center, or specialty pediatric clinic within the past 6 months? (Y/N) (Buchanan et al., 2022)	*n* = 1/CMC (*n* = 1)
the Bob's Level of Support Scale (BLSS)	No	origin unknown	The BLSS was designed for use in a developmental pediatrics clinic to screen families’ needs for increased care management support The BLSS assesses different levels of support for five dimensions: health services, family support services, behavioral and mental health services, educational services, and special issues (only available in included article, no ref.) (Crowley et al., 2018)	*n* = 1/CSHCN (*n* = 1)

*CCC* Complex Chronic Conditions, *CCHC* Children with complex health conditions, *CMC* Children with medical complexity, *C(Y)SHCN* Children (and Youth) with Special Healthcare Needs, *LLC* Life Limiting Conditions, *MCC* Medically complex children, *PCC* Pediatric Chronic Condition, *PCCI* Pediatric Chronic Critical Illness, *PMID* Profound and Multiple Intellectual Disabilities, *TD* Technology Dependence

## Discussion

This scoping review aimed to provide a comprehensive overview of the patient categories, definitions, and operationalizations used in studies concerning pediatric chronic patients with varying degrees of complexity in all care settings. We identified 522 studies on pediatric chronic patients published over the past five years. Notably, 82% of the studies used one of 11 existing definitions, suggesting some consensus in the field. However, the remaining 18% showed considerable heterogeneity in terminology and definitions, indicating further standardization is needed. Only 34% of studies described how definitions were operationalized, and just 20% used standardized tools, revealing a gap between conceptual definitions and their practical application in research and practice.

### Definitions and operationalizations

Establishing a clear distinction between the definitions of pediatric chronic patients remains challenging. Many definitions share similar criteria, such as chronicity and functional limitations, which contributes to blurred lines between patient categories. However, for certain patient categories, consistent use of specific definitions is evident. In particular, the definitions of CMC by Cohen et al. [4], and of CSHCN by McPherson et al. [6] are frequently used, and appear to be widely accepted within the field. Nevertheless, the definitions proposed by Cohen et al. [4], Feudtner et al. [18], and Simon et al. [19] are used interchangeably for CMC, CCC, and TD. This may support the argument put forth by Azar et al., who suggest a single, combined category for children with complex health conditions [10]. However, such an approach may be too simplistic, as each patient category is associated with unique demographic, clinical, and healthcare usage characteristics as described in the respective definitions [40]. To date, it remains difficult to map these features, partly due to the lack of clear operationalization tools. This may explain why only one-third of the studies included in this review operationalized the definitions.

Furthermore, even when tools exist, they are often applied to other patient categories than those for which they were originally intended. It is therefore recommended to select the tool that aligns with the study goals and characteristics of the patient population. For example, the Pediatric Medical Complexity Algorithm and Complex Chronic Condition Classification System should not be used interchangeably, because each patient category is designed for a specific patient population with unique clinical characteristics, and resource utilization. Importantly, there is still a lack of consensus on how to measure multisystem complexity [40].

In the included studies, the majority of definitions and operationalizations focused on medical diagnoses using ICD codes, with some incorporating additional criteria such as healthcare utilization and technology use. An advantage of the use of ICD codes is that they are standardized registrations, mostly well-documented in medical records and registries. This makes these data available for both prospective and retrospective evaluations. However, as recognized in the literature, the complexity of chronic conditions extends beyond medical diagnoses [41–44]. The burden of a chronic condition also includes significant psychological, social, and financial difficulties [45]. These factors are critical in understanding the full impact of pediatric chronic diseases, as they are often associated with higher healthcare costs and a diminished quality of life [46–48]. Some operationalization tools also include socioeconomic and psychosocial circumstances, mostly reported by patients or parents, limiting their applicability in retrospective research based on administrative data [7, 30, 31, 34]. Nevertheless, a shift from the medical model toward a more biopsychosocial model of health, that highlights the importance of a holistic perspective on the needs of patients and their families, would not only enhance the accuracy of patient identification, but may also support more effective, patient- and family-centered care. To help future tool development, we recommend including a minimum biopsychosocial element set, comprising functional status, technology dependence, healthcare utilization and psychosocial and family needs, to improve the quality of patient-centered assessments of chronic conditions in pediatric patients.

Approximately one-third of the operationalized definitions identified seemed to be practiced or opinion-based rather than based on standardized or validated criteria. While measurable elements were mentioned, the origin was unclear or they were derived from local expertise. This aligns with previous literature [49, 50]. One review reported that many descriptions of pediatric chronic critical illness were based on researchers’ opinions [49]. Another review reported significant differences in the assessment method of long-term morbidity, depending on the physician conducting the evaluation [50]. Reliance on local research opinions may limit the generalizability of findings and, combined with the lack of systematic operationalization, may undermine consistency, comparability and applicability across research and clinical practice.

Additionally, results showed that the term ‘children with special healthcare needs’ is still widely used as an established term, particularly in US surveillance and policy contexts. While both, Psychological Association [51] and the United Nations [52] recommend using disability inclusive language, replacing this term with “children with disabilities” for person-centered communication, it is important to keep in mind that these recommendations are different from the terms used in measurement and prevalence reports for research.

### Strengths and limitations

A first strength of this scoping review is its comprehensive overview of definitions and operationalizations used to describe pediatric chronic patients, in contrast to previous reviews that focused on specific patient categories [49, 53, 54]. Second, the independent verification of both the study screening and data charting phases minimized inconsistencies and ensured objective data evaluation [55, 56]. Third, the multidisciplinary composition of the research team contributed diverse perspectives and expertise, enhancing the robustness of the findings [57].

Nevertheless, some limitations should be acknowledged. First, the search was limited to the period 2018–2023. While we acknowledge that there is considerable literature prior to this period, our rationale was that recent studies typically build upon and reference earlier foundational work. This expectation was confirmed during data extraction, as we observed that key definitions and operationalizations from older studies were cited and incorporated in the more recent literature. Second, definitions that we considered similar were merged, which facilitated a coherent analytical framework but may have resulted in the loss of some conceptual nuances. Third, while the review identified operationalization tools, it did not assess their psychometric properties or practical applicability in clinical or research settings. Fourth, the grey literature, such as policy documents and organizational definitions, was not included in the search strategy. As a result, relevant definitions published outside academic journals may have been missed.

### Implications for future research and practice

We strongly recommend consistent use of patient categories and corresponding definitions for pediatric chronic patients to promote more uniformity and consistency across research and clinical practice. Operationalization tools should be in accordance with the specific definitions for which they were originally developed. When operationalization tools for specific definitions are lacking, priority should be given to the development and validation of these tools using a biopsychosocial model of health. Importantly, their usability should be assessed in the context of both prospective and retrospective research to enhance their practical relevance across diverse settings.

## Conclusion

A significant number of definitions are used across different patient categories in pediatric chronic care research. These definitions are heterogeneously formulated and used interchangeably, resulting in conceptual ambiguity. Moreover, only a minority of studies reported the use of operationalization tools. Achieving consensus on factors that define different patient categories and levels of complexity in pediatric chronic care research, along with greater uniformity in operationalization tools would represent an important step forward. Such standardization may enhance the quality and comparability of research, support the quality of clinical care, and enable more efficient use of healthcare resources.

## Supplementary Information

Below is the link to the electronic supplementary material.

ESM 1 Online resource 1 (PDF 233 KB)

ESM 2 Online resource 2 (PDF 89.0 KB)

ESM 3 Online resource 3 (PDF 167 KB)

ESM 4 Online resource 4 (PDF 303 KB)

ESM 5 Online resource 5 (PDF 141 KB)

ESM 6 Online resource 6 (PDF 129 KB)

## Data Availability

No datasets were generated or analysed during the current study.

## Abbreviations

ACCAPED: Assessment of Complex Clinical Assistance Needs in Pediatrics
AMG: Adjusted Morbidity Groups classification system
BLSS: Bob’s Level of Support Scale
CCC: Children with Complex Chronic Conditions
CCCCS: Complex Chronic Condition Classification System
CCHC: Children with Complex Health Conditions
CCI: Chronic Condition Indicator
CMC: Children with Medical Complexity
CRG: 3M Clinical Risk Group classification
CWDA: Children With Disabilities Algorithm
CYSHCN: Children and Youth with Special Healthcare Needs
GMFCS: Gross Motor Function Classification System
ICD: International Classification of Diseases
LLC: Children with Life Limiting Conditions
MCC: Medically Complex Children
NSCH: National Survey Children’s Health
OTA: Office of Technology Assessment
PCC: Pediatric Chronic Condition
PCCI: Pediatric Chronic Critical Illness
PMCA: Pediatric Medical Complexity Algorithm
PMID: Profound and Multiple Intellectual Disabilities
SODCMC: Standard Operational Definition for Children with Medical Complexity
TD: Children with Technology Dependence
