# Supplementary material for: Definitions and operationalizations of pediatric chronic patients: a scoping review
Source: Eur J Pediatr. 2025 Nov 25;184(12):789. doi: 10.1007/s00431-025-06556-0 (PMC12644160; doi:10.1007/s00431-025-06556-0)
Supplement: Supplementary file 2 — Online resource 2 (PDF 89.0 KB) [file 431_2025_6556_MOESM2_ESM.pdf]

Definitions and operationalizations of pediatric chronic patients: A scoping review

European Journal of Pediatrics

Cor-Jan van der Perk (CJP) [a,b,c], MSc, RN; Lisa-Maria van Klaveren (LK) [c,d], MSc, MA; Karlijn S. Timmer (KT) [a,b,c], MSc, RN, Heleen N. Haspels [a,e] , MSc; Faridi S. Jamaludin [f], Lotte Haverman [a] PhD; Willem B. de Vries [a], MD, PhD; Anne M. Eskes (AE) [g], RN, PhD; Jolanda M. Maaskant (JM) [a,b,h], RN, PhD

Affiliations [a]Amsterdam UMC, Emma Children's Hospital, Amsterdam, the Netherlands; [b]Amsterdam Reproduction & Development Research Institute, Amsterdam, the Netherlands; [c]Amsterdam Public Health, Amsterdam, the Netherlands; [d]Amsterdam UMC, Institute of Education and Training, Amsterdam, the Netherlands; [e]Erasmus Medical Centre, Sophia Children's Hospital, Department of Pediatric Intensive Rotterdam, the Netherlands; [f]Research support, Medical Library, Amsterdam UMC, University of Amsterdam, Amsterdam, the Netherlands; [g]Amsterdam UMC, Department of Surgery, Amsterdam, the Netherlands; [h]Amsterdam UMC, Department of Internal Medicine, Amsterdam, the Netherlands

Corresponding author address: Cor-Jan van der Perk, Emma Children's Hospital Amsterdam UMC, University of Amsterdam, Meibergdreef 9, 1105 AZ, Amsterdam the Netherlands, c.j.vanderperk@amsterdamumc.nl

Online Resource 2: Patient categories chronic conditions and complex care needs

Catastrophic medical complexity
Children with medical complexity (CMC)
Children with complex chronic disease
Children with special healthcare needs (CSHCN)
Children and Youth with special health care needs (CYSHCN)
Children with chronic conditions and complex care needs
Children with complex chronic conditions
Children with technology dependence
Complex care needs (CCN)
Complex needs
Complex healthcare needs
Complex health care needs
Complex medical needs
Complex medical care needs
Complex medical condition
Complex learning difficulties and disabilities (CLDD)
Complex and/or profound needs
Complex and Integrated Care Needs
Complex health conditions
Life limiting conditions
Medically complex children

Medically fragile children
Multiple complex needs (MCN)
Multisystem complexity
Pediatric Medical Complexity
Pediatric Chronic Critical Illness
Profound needs
Profound and multiple intellectual disabilities (PMID)
Profound intellectual and multiple disabilities (PIMD)
Severe medical conditions
Technology dependent
