# Supplementary material for: Definitions and operationalizations of pediatric chronic patients: a scoping review
Source: Eur J Pediatr. 2025 Nov 25;184(12):789. doi: 10.1007/s00431-025-06556-0 (PMC12644160; doi:10.1007/s00431-025-06556-0)
Supplement: Supplementary file 3 — Online resource 3 (PDF 167 KB) [file 431_2025_6556_MOESM3_ESM.pdf]

Definitions and operationalizations of pediatric chronic patients: A scoping review

European Journal of Pediatrics

Cor-Jan van der Perk (CJP) [a,b,c], MSc, RN; Lisa-Maria van Klaveren (LK) [c,d], MSc, MA; Karlijn S. Timmer (KT) [a,b,c], MSc, RN, Heleen N. Haspels [a,e] , MSc; Faridi S. Jamaludin [f], Lotte Haverman [a] PhD; Willem B. de Vries [a], MD, PhD; Anne M. Eskes (AE) [g], RN, PhD; Jolanda M. Maaskant (JM) [a,b,h], RN, PhD

Affiliations [a]Amsterdam UMC, Emma Children's Hospital, Amsterdam, the Netherlands; [b]Amsterdam Reproduction & Development Research Institute, Amsterdam, the Netherlands; [c]Amsterdam Public Health, Amsterdam, the Netherlands; [d]Amsterdam UMC, Institute of Education and Training, Amsterdam, the Netherlands; [e]Erasmus Medical Centre, Sophia Children's Hospital, Department of Pediatric Intensive Rotterdam, the Netherlands; [f]Research support, Medical Library, Amsterdam UMC, University of Amsterdam, Amsterdam, the Netherlands; [g]Amsterdam UMC, Department of Surgery, Amsterdam, the Netherlands; [h]Amsterdam UMC, Department of Internal Medicine, Amsterdam, the Netherlands

Corresponding author address: Cor-Jan van der Perk, Emma Children's Hospital Amsterdam UMC, University of Amsterdam, Meibergdreef 9, 1105 AZ, Amsterdam the Netherlands, c.j.vanderperk@amsterdamumc.nl

Online Resource 3. Search strategy

MEDLINE

#	Searches	Results
1	(exp Child/ or Adolescent/ or exp Infant/) and *Chronic Disease/cl, ep	1163
2	(Intensive Care Units, Pediatric/ or (PICU or PICUs).ti,ab,kf.) and (Critical Illness/ or Critical Care/ or critical*.ti,ab,kf.) and (Chronic Disease/ or chronic*.ti,ab,kf.)	255
3	((child* or pediatric* or paediatric* or youth* or adolescen* or infan*) adj3 ("medical complexit* " or "complex care need*" or "complex need*" or "profound need*" or "multiple complex need*" or "multiple intellectual disabilit*" or "complex chronic condition*" or "complex chronic disease*" or "complex medical condition*" or "complex healthcare need*" or "complex health care need*" or "complex health condition*" or "complex medical care need*" or "complex medical need*" or "special healthcare need*" or "special health care need*" or "medically complex" or "medically fragile" or "technology dependen*" or "catastrophic medical complexit*" or "severe medical	3817

	condition*" or "life limiting condition*" or "integrated care need*" or "multisystem complexit*" or "medical complex*" or "complex learning difficult*" or "complex learning disabilit*" or "chronic critical ill*" or "chronic health condition*")).ti,ab,kf.	
4	("profound intellectual and multiple disabilit*" or "profound and multiple intellectual disabilit*" or "complex and integrated care need*" or CYSHCN or CSHCN).ti,ab,kf.	719
5	(child* and "complex chronic condition*").ti,ab,kf.	434
6	1 or 2 or 3 or 4 or 5	5476
7	limit 6 to yr="1998 -Current"	5116
8	editorial/ or letter/ or published erratum/ or (letter or comment* or editorial).ti.	2120343
9	7 not 8	5002
10	Adult/ not (exp Child/ or Adolescent/ or exp Infant/)	3712307
11	9 not 10	4950

EMBASE

#	Searches	Results
1	(child health/ or exp child/ or exp adolescent/ or exp infant/) and (*chronic disease/ or multiple chronic conditions/) and (disease classification/ or epidemiology.fs.)	725
2	(pediatric intensive care unit/ or (PICU or PICUs).ti,ab,kf.) and (critical illness/ or intensive care/ or critical*.ti,ab,kf.) and (exp chronic disease/ or chronic*.ti,ab,kf.)	611
3	((child* or pediatric* or paediatric* or youth* or adolescen* or infan*) adj3 ("medical complexit* " or "complex care need*" or "complex need*" or "profound need*" or "multiple complex need*" or "multiple intellectual disabilit*" or "complex chronic condition*" or "complex chronic disease*" or "complex medical condition*" or "complex healthcare need*" or "complex health care need*" or "complex health condition*" or "complex medical care need*" or "complex medical need*" or "special healthcare need*" or "special health care need*" or "medically complex" or "medically fragile" or "technology dependen*" or "catastrophic medical complexit*" or "severe medical condition*" or "life limiting condition*" or "integrated care need*" or "multisystem complexit*" or "medical complex*" or "complex learning difficult*" or "complex learning disabilit*" or "chronic critical ill*" or "chronic health condition*")).ti,ab,kf.	5142
4	("profound intellectual and multiple disabilit*" or "profound and multiple intellectual disabilit*" or "complex and integrated care need*" or CYSHCN or CSHCN).ti,ab,kf.	909
5	(child* and "complex chronic condition*").ti,ab,kf.	660
6	1 or 2 or 3 or 4 or 5	6899
7	limit 6 to yr="1998 -Current"	6583
8	letter/ or editorial/ or note/ or (letter or comment* or editorial).ti.	2943473
9	7 not 8	6415
10	adult/ not (exp child/ or exp adolescent/ or exp infant/)	8062399
11	9 not 10	6247

PsycINFO

#	Searches	Results
1	((child* or pediatric* or paediatric* or youth* or adolescen* or infan*) adj3 ("medical complexit* " or "complex care need*" or "complex need*" or "profound need*" or "multiple complex need*" or "multiple intellectual disabilit*" or "complex chronic condition*" or "complex chronic disease*" or "complex medical condition*" or "complex healthcare need*" or "complex health care need*" or "complex health condition*" or "complex medical care need*" or "complex medical need*" or "special healthcare need*" or "special health care need*" or "medically complex" or "medically fragile" or "technology dependen*" or "catastrophic medical complexit*" or "severe medical condition*" or "life limiting condition*" or "integrated care need*" or "multisystem complexit*" or "medical complex*" or "complex learning difficult*" or "complex learning disabilit*" or "chronic critical ill*" or "chronic health condition*")).ti,ab,id.	1636
2	("profound intellectual and multiple disabilit*" or "profound and multiple intellectual disabilit*" or "complex and integrated care need*" or CYSHCN or CSHCN).ti,ab,id.	497
3	(child* and "complex chronic condition*").ti,ab,id.	64
4	pediatrics/ and chronic illness/	602
5	chronically ill children/	453
6	1 or 2 or 3 or 4 or 5	2794
7	limit 6 to yr="1998 -Current"	2496
8	(letter or comment* or editorial).ti.	54997
9	7 not 8	2468

Cochrane Library

#	Searches	Results
1	((child* or pediatric* or paediatric* or youth* or adolescen* or infan*) NEAR/3 ("medical complexit* " or "complex care need*" or "complex need*" or "profound need*" or "multiple complex need*" or "multiple intellectual disabilit*" or "complex chronic condition*" or "complex chronic disease*" or "complex medical condition*" or "complex healthcare need*" or "complex health care need*" or "complex health condition*" or "complex medical care need*" or "complex medical need*" or "special healthcare need*" or "special health care need*" or "medically complex" or "medically fragile" or "technology dependen*" or "catastrophic medical complexit*" or "severe medical condition*" or "life limiting condition*" or "integrated care need*" or "multisystem complexit*" or "medical complex*" or "complex learning difficult*" or "complex learning disabilit*" or "chronic critical ill*" or "chronic health condition*")):ti,ab,kw	45
2	(profound and (multiple disabilit* OR intellectual disabilit*)):ti,ab,kw	96
3	(complex and integrated care need*):ti,ab,kw	247
4	(child* and "complex chronic condition*"):ti,ab,kw	3
5	(CYSHCN or CSHCN):ti,ab,kw	10

6	#1 or #2 or #3 or #4 or #5	395

CINAHL

Searches	Results
(MM "Child, Medically Fragile")<br><br>OR<br><br>TI ((child* or pediatric* or paediatric* or youth* or adolescen* or infan*) N3 ("medical complexit* " or "complex care need*" or "complex need*" or "profound need*" or "multiple complex need*" or "multiple intellectual disabilit*" or "complex chronic condition*" or "complex chronic disease*" or "complex medical condition*" or "complex healthcare need*" or "complex health care need*" or "complex health condition*" or "complex medical care need*" or "complex medical need*" or "special healthcare need*" or "special health care need*" or "medically complex" or "medically fragile" or "technology dependen*" or "catastrophic medical complexit*" or "severe medical condition*" or "life limiting condition*" or "integrated care need*" or "multisystem complexit*" or "medical complex*" or "complex learning difficult*" or "complex learning disabilit*" or "chronic critical ill*" or "chronic health condition*")) OR AB ((child* or pediatric* or paediatric* or youth* or adolescen* or infan*) N3 ("medical complexit* " or "complex care need*" or "complex need*" or "profound need*" or "multiple complex need*" or "multiple intellectual disabilit*" or "complex chronic condition*" or "complex chronic disease*" or "complex medical condition*" or "complex healthcare need*" or "complex health care need*" or "complex health condition*" or "complex medical care need*" or "complex medical need*" or "special healthcare need*" or "special health care need*" or "medically complex" or "medically fragile" or "technology dependen*" or "catastrophic medical complexit*" or "severe medical condition*" or "life limiting condition*" or "integrated care need*" or "multisystem complexit*" or "medical complex*" or "complex learning difficult*" or "complex learning disabilit*" or "chronic critical ill*" or "chronic health condition*"))<br><br>OR<br><br>TI ("profound intellectual and multiple disabilit*" or "profound and multiple intellectual disabilit*" or "complex and integrated care need*" or CYSHCN or CSHCN) OR AB ("profound intellectual and multiple disabilit*" or "profound and multiple intellectual disabilit*" or "complex and integrated care need*" or CYSHCN or CSHCN)<br><br>OR<br><br>TI (child* and "complex chronic condition*") OR AB (child* and "complex chronic condition*")<br><br>**NOT**<br><br>TI letter or comment* or editorial<br><br>**NOT**<br><br>(MH "Adult") NOT ((MH "Child+") OR (MH "Adolescence+") OR (MH "Infant+"))	3944
