# Supplementary material for: Definitions and operationalizations of pediatric chronic patients: a scoping review
Source: Eur J Pediatr. 2025 Nov 25;184(12):789. doi: 10.1007/s00431-025-06556-0 (PMC12644160; doi:10.1007/s00431-025-06556-0)
Supplement: Supplementary file 4 — Online resource 4 (PDF 303 KB) [file 431_2025_6556_MOESM4_ESM.pdf]

Definitions and operationalizations of pediatric chronic patients: A scoping review

European Journal of Pediatrics

Cor-Jan van der Perk (CJP) [a,b,c], MSc, RN; Lisa-Maria van Klaveren (LK) [c,d], MSc, MA; Karlijn S. Timmer (KT) [a,b,c], MSc, RN, Heleen N. Haspels [a,e] , MSc; Faridi S. Jamaludin [f], Lotte Haverman [a] PhD; Willem B. de Vries [a], MD, PhD; Anne M. Eskes (AE) [g], RN, PhD; Jolanda M. Maaskant (JM) [a,b,h], RN, PhD

Affiliations [a]Amsterdam UMC, Emma Children's Hospital, Amsterdam, the Netherlands; [b]Amsterdam Reproduction & Development Research Institute, Amsterdam, the Netherlands; [c]Amsterdam Public Health, Amsterdam, the Netherlands; [d]Amsterdam UMC, Institute of Education and Training, Amsterdam, the Netherlands; [e]Erasmus Medical Centre, Sophia Children's Hospital, Department of Pediatric Intensive Rotterdam, the Netherlands; [f]Research support, Medical Library, Amsterdam UMC, University of Amsterdam, Amsterdam, the Netherlands; [g]Amsterdam UMC, Department of Surgery, Amsterdam, the Netherlands; [h]Amsterdam UMC, Department of Internal Medicine, Amsterdam, the Netherlands

Corresponding author address: Cor-Jan van der Perk, Emma Children's Hospital Amsterdam UMC, University of Amsterdam, Meibergdreef 9, 1105 AZ, Amsterdam the Netherlands, c.j.vanderperk@amsterdamumc.nl

Online Recource 4: References to definitions

Online Recource 4A: Reference list Children with Medical Complexity (CMC)

**Definition CMC (n=187)**	
Definition according to Cohen et al. (2011)	(1-166)
Definition Cohen with additional information	(167-176)
**Studies using other term patient category with definition Cohen et al. (n=11)**	
Pediatric medical complexity	(177)
Medically complex children	(178-182)
Infants or neonates with medical complexity	(183-187)

1. Abebe E, Scanlon MC, Chen H, Yu D. Complexity of Documentation Needs for Children With Medical Complexity: Implications for Hospital Providers. Hospital pediatrics. 2020;10(8):670-8.

2. Abebe E, Scanlon MC, Lee KJ, Chui MA. What do family caregivers do when managing medications for their children with medical complexity? Applied ergonomics. 2020;87:103-108.
3. Adams S, Beatty M, Moore C, Desai A, Bartlett L, Culbert E, et al. Perspectives on team communication challenges in caring for children with medical complexity. BMC health services research. 2021;21(1):300.
4. Adams S, Nicholas D, Mahant S, Weiser N, Kanani R, Boydell K, et al. Care maps and care plans for children with medical complexity. Child: care, health and development. 2019;45(1):104-10.
5. Amarri S, Ottaviani A, Campagna A, De Panfilis L. Children with medical complexity and paediatric palliative care: a retrospective cross-sectional survey of prevalence and needs. Italian journal of pediatrics. 2021;47(1):110.
6. Antolick MM, Looman WS, Cady RG, Kubiatowicz K. Identifying and Communicating Postdischarge Goals for Hospitalized Children With Medical Complexity: A Process Improvement Pilot in a Specialty Pediatric Setting. Journal of pediatric health care : official publication of National Association of Pediatric Nurse Associates & Practitioners. 2020;34(2):90-8.
7. Aryee E, Perrin JM, Clancy S, Merrill C, Curran M, Oreskovic NM. Mental Health of Caregivers of Children with Medical Complexity During COVID-19. Journal of developmental and behavioral pediatrics : JDBP. 2023;44(3):e212-e7.
8. Barnert ES, Coller RJ, Nelson BB, Thompson LR, Tran J, Chan V, et al. Key Population Health Outcomes for Children with Medical Complexity: A Systematic Review. Maternal and child health journal. 2019;23(9):1167-76.
9. Batson L, Donohue PK, Boss RD, Seltzer RR. Family challenges in personal transportation of children with medical complexity. Journal of pediatric rehabilitation medicine. 2022;15(4):655-65.
10. Bergman DA, Keller D, Kuo DZ, Lerner C, Mansour M, Stille C, et al. Costs and Use for Children With Medical Complexity in a Care Management Program. Pediatrics. 2020;145(4):1-10.
11. Buchanan F, Cohen E, Milo-Manson G, Shachak A. What makes difficult decisions so difficult?: An activity theory analysis of decision making for physicians treating children with medical complexity. Patient Educ Couns. 2020;103(11):2260-8.
12. Bucholz EM, Toomey SL, Schuster MA. Trends in Pediatric Hospitalizations and Readmissions: 2010-2016. Pediatrics. 2019;143(2):1-10.
13. Cohen SS, Toly VB, Lerret SM, Sawin KJ. The Impact of COVID-19 on Systems of Care for Children and Youth With Special Health Care Needs. Journal of Pediatric Health Care. 2023;37(2):106-16.
14. Dewan T, Birnie K, Drury J, Jordan I, Miller M, Neville A, et al. Experiences of medical traumatic stress in parents of children with medical complexity. Child: care, health and development. 2023;49(2):292-303.
15. Diskin C, Buchanan F, Cohen E, Dewan T, Diaczun T, Gordon M, et al. The impact of the COVID-19 pandemic on children with medical complexity. BMC pediatrics. 2022;22(1):496.
16. Driansky A, Pilapil M, Mastrogiannis A. Updating the healthcare maintenance visit for children with medical complexity: applying lessons learned from the coronavirus disease 2019 pandemic. Current opinion in pediatrics. 2022;34(2):248-54.

17. Ferro F, Tozzi AE, Erba I, Dall'Oglio I, Campana A, Cecchetti C, et al. Impact of telemedicine on health outcomes in children with medical complexity: an integrative review. European journal of pediatrics. 2021;180(8):2389-400.
18. Foster CC, Fuentes MM, Wadlington LA, Jacob-Files E, Desai AD, Simon TD, et al. Caregiver and provider experiences of physical, occupational, and speech therapy for children with medical complexity. Journal of pediatric rehabilitation medicine. 2021;14(3):505-16.
19. Foster CC, Kwon S, Whitlow L, Cullen JP, Agrawal RK, Goodman D, et al. Connecting Hospital to Home: Characteristics of and Rehospitalization Rates in Hospitalized Children With Private-Duty Nursing. Hospital pediatrics. 2019;9(7):530-7.
20. Foster CC, Shaunfield S, Black LE, Labellarte PZ, Davis MM. Improving Support for Care at Home: Parental Needs and Preferences When Caring for Children with Medical Complexity. Journal of pediatric health care : official publication of National Association of Pediatric Nurse Associates & Practitioners. 2022;36(2):154-64.
21. Frankel H, Matiz LA, Friedman S. Siblings of Children with Medical Complexity-A Vulnerable Population in the Medical Home. Journal of health care for the poor and underserved. 2022;33(2):702-13.
22. Fratantoni K, Raisanen JC, Boss RD, Miller J, Detwiler K, Huff SM. The Pediatric Home Health Care Process: Perspectives of Prescribers, Providers, and Recipients. Pediatrics. 2019;144(3):1-9.
23. Gower WA, Golden SL, King NMP, Nageswaran S. Decision Making about Tracheostomy for Children with Medical Complexity: Caregiver and Healthcare Provider Perspectives. Academic pediatrics. 2020:1094-100.
24. Hamilton H, West AN, Ammar N, Chinthala L, Gunturkun F, Jones T, et al. Analyzing Relationships Between Economic and Neighborhood-Related Social Determinants of Health and Intensive Care Unit Length of Stay for Critically Ill Children With Medical Complexity Presenting With Severe Sepsis. Frontiers in public health. 2022;10:789999.
25. Hsieh Y-H, Borgestig M, Gopalarao D, McGowan J, Granlund M, Hwang A-W, et al. Communicative Interaction with and without Eye-Gaze Technology between Children and Youths with Complex Needs and Their Communication Partners. International journal of environmental research and public health. 2021;18(10):5134.
26. Huang L, Freed GL, Dalziel K. Children With Special Health Care Needs: How Special Are Their Health Care Needs? Academic pediatrics. 2020;20(8):1109-15.
27. Ishihara T, Tanaka H. Factors affecting tracheostomy in critically ill paediatric patients in Japan: a data-based analysis. BMC pediatrics. 2020;20(1):237.
28. Jacobs S, Davies N, Butterick KL, Oswell JL, Siapka K, Smith CH. Shared decision-making for children with medical complexity in community health services: a scoping review. BMJ paediatrics open. 2023;7(1):e001866.
29. Kosaka M, Murata N, Kaneda Y, Kotera Y, Sagara S, Masunaga H, et al. Challenges when going on excursions with children with medical complexity in Japan. Pediatrics international : official journal of the Japan Pediatric Society. 2023;65(1):e15403.
30. Kusma JD, Davis MM, Foster C. Characteristics of Medicaid Policies for Children With Medical Complexity by State: A Qualitative Study. JAMA network open. 2022;5(10):e2239270.

31. Lawrence PR, Spratling R. A Theory for Understanding Parental Workload and Capacity to Care for Children With Medical Complexity. Research and theory for nursing practice. 2022;36(1):34-46.
32. Leach KF, Stack NJ, Jones S. Optimizing the multidisciplinary team to enhance care coordination across the continuum for children with medical complexity. Current problems in pediatric and adolescent health care. 2021;51(12):101128.
33. Lin E, Scharbach K, Liu B, Braun M, Tannis C, Wilson K, et al. A Multidisciplinary Home Visiting Program for Children With Medical Complexity. Hospital pediatrics. 2020;10(11):925-31.
34. Lin JL, Clark CL, Halpern-Felsher B, Bennett PN, Assis-Hassid S, Amir O, et al. Parent Perspectives in Shared Decision-Making for Children With Medical Complexity. Academic pediatrics. 2020;20(8):1101-8.
35. Lindstrom K, Cady R, Bushaw A. Family-centered care for children with medical complexity: A goal-planning initiative. The Nurse practitioner. 2020;45(8):49-55.
36. Looman WS, Park YS, Gallagher TT, Weinfurter EV. Outcomes research on children with medical complexity: A scoping review of gaps and opportunities. Child: care, health and development. 2020;46(1):121-31.
37. Mai K, Davis RK, Hamilton S, Robertson-James C, Calaman S, Turchi RM. Identifying Caregiver Needs for Children With a Tracheostomy Living at Home. Clinical pediatrics. 2020;59(13):1169-81.
38. Maypole J, Gavin T, de Banate MA, Sadof M. Lessons Learned, Best Practices: Care Coordination for Children with Medical Complexity. Pediatric annals. 2020;49(11):e457-e66.
39. McKiernan A, Carr A, O'Keeffe L, Butler E, Quinn C, Guerin S. Levels of satisfaction with children's respite services, parental coping and family functioning. Health & social care in the community. 2020;28(2):568-75.
40. Meehan E, D'Aprano A L, Gibb SM, Mountford NJ, Williams K, Harvey AR, et al. Comprehensive care programmes for children with medical complexity. Cochrane Database of Systematic Reviews. 2019;2019(5):CD013329.
41. Mitchell SM. True Resilience: A Look Inside COVID's Effect on Children with Medical Complexity and Their Families. Current pediatrics reports. 2021;9(4):171-7.
42. Mooney-Doyle K, Lindley LC. The Association between Poverty and Family Financial Challenges of Caring for Medically Complex Children. Nursing Economic$. 2019;37(4):198-208.
43. Mooney-Doyle K, Lindley LC. Family and Child Characteristics Associated With Caregiver Challenges for Medically Complex Children. Family & community health. 2020;43(1):74-81.
44. Morse BL, Serna RW, LaLumiere M, Rogal M, Foley K, Lombardo M, et al. Leveraging Parent Pain Perspectives to Improve Pain Practices for Children with Medical Complexity. Pain management nursing : official journal of the American Society of Pain Management Nurses. 2021;22(2):169-76.
45. Morse BL, Solodiuk JC, Greco CD, Mauskar S, Hauer J. Initial Validation of GRASP: A Differential Diagnoses Algorithm for Children With Medical Complexity and an Unknown Source of Pain. Hospital pediatrics. 2020;10(8):633-40.

46. Murphy NA, Alvey J, Valentine KJ, Mann K, Wilkes J, Clark EB. Children With Medical Complexity: The 10-Year Experience of a Single Center. Hospital pediatrics. 2020;10(8):702-8.
47. Murphy S, Ehritz C. Clinical Nurse Specialist Practice Strategies for Children With Medical Complexity. Clinical nurse specialist CNS. 2021;35(1):38-43.
48. Nageswaran S, Banks Q, Golden SL, Gower WA, King NMP. The role of religion and spirituality in caregiver decision-making about tracheostomy for children with medical complexity. Journal of health care chaplaincy. 2022;28(1):95-107.
49. Nageswaran S, Ellis MB, Beveridge MS. Communication Challenges Faced by Spanish-Speaking Caregivers of Children with Medical Complexity: a Qualitative Study. Journal of racial and ethnic health disparities. 2022;9(6):2218-26.
50. Nageswaran S, Gower WA, King NMP, Golden SL. Tracheostomy decision-making for children with medical complexity: What supports and resources do caregivers need? Palliative & supportive care. 2022:1-7.
51. Nageswaran S, Sebesta MR, Golden SL. Transitioning Children With Medical Complexity From Hospital to Home Health Care: Implications for Hospital-Based Clinicians. Hospital pediatrics. 2020;10(8):657-62.
52. Nassel D, Chartrand C, Dore-Bergeron M-J, Lefebvre F, Ballantyne M, Van Overmeire B, et al. Very Preterm Infants with Technological Dependence at Home: Impact on Resource Use and Family. Neonatology. 2019;115(4):363-70.
53. Nkoy FL, Hofmann MG, Stone BL, Poll J, Clark L, Fassl BA, et al. Information needs for designing a home monitoring system for children with medical complexity. International journal of medical informatics. 2019;122:7-12.
54. Onofri A, Pavone M, De Santis S, Verrillo E, Caggiano S, Ullmann N, et al. Telemedicine in children with medical complexity on home ventilation during the COVID-19 pandemic. Pediatric pulmonology. 2021;56(6):1395-400.
55. Parente V, Parnell L, Childers J, Spears T, Jarrett V, Ming D. Point-of-Care Complexity Screening Algorithm to Identify Children With Medical Complexity. Hospital pediatrics. 2021 Jan;11(1):44-51.
56. Parpia C, Moore C, Beatty M, Miranda S, Adams S, Stinson J, et al. Evaluation of a Secure Messaging System in the Care of Children With Medical Complexity: Mixed Methods Study. JMIR formative research. 2023;7:e42881.
57. Passos Dos Santos R, Macdonald ME, Carnevale FA. Moral experiences of children with medical complexity: A participatory hermeneutic ethnography in Brazil. Journal of child health care : for professionals working with children in the hospital and community. 2022:13674935221112156.
58. Pitch N, Davidson L, Mekhuri S, Patel R, Patel S, Ambreen M, et al. Exploring the experience of family caregivers of children with medical complexity during COVID-19: a qualitative study. BMC pediatrics. 2023;23(1):160.
59. Quartarone S, Lilian Lin JL, Orkin J, Fayed N, French S, Major N, et al. Implementing a Care Coordination Strategy for Children with Medical Complexity in Ontario, Canada: A Process Evaluation. International Journal of Integrated Care (IJIC). 2022;22(2):1-14.
60. Rennick JE, St-Sauveur I, Knox AM, Ruddy M. Exploring the experiences of parent caregivers of children with chronic medical complexity during pediatric intensive care unit hospitalization: an interpretive descriptive study. BMC pediatrics. 2019;19(1):272.

61. Rojas CR, Moore A, Coffin A, McClam C, Ehritz C, Hogan A, et al. Medication Rounds: A Tool to Promote Medication Safety for Children with Medical Complexity. Joint Commission journal on quality and patient safety. 2023;49(4):226-34.
62. Sadof M, Carlin S, Brandt S, Maypole J. A Step-by-Step Guide to Building a Complex Care Coordination Program in a Small Setting. Clinical pediatrics. 2019;58(8):897-902.
63. Santos RPD, Macdonald ME, Carnevale F. A scoping review of the moral experiences of children with medical complexity in Brazil. Revista brasileira de enfermagem. 2020;73(2):e20190268.
64. Scarselli A, Smarrazzo A, De Sanctis F, Rava L, Carletti M, Ciofi Degli Atti M, et al. The burden of central line-associated bloodstream infections in children with medical complexity. The journal of vascular access. 2023;24(2):198-204.
65. Seltzer RR, Raisanen JC, Williams EP, Da Silva T, Donohue PK, Boss RD. Exploring Medical Foster Care as a Placement Option for Children With Medical Complexity. Hospital pediatrics. 2019;9(9):697-706.
66. Soscia J, Adams S, Cohen E, Moore C, Friedman JN, Gallagher K, et al. The parental experience and perceptions of blenderized tube feeding for children with medical complexity. Paediatrics & child health. 2021;26(8):462-9.
67. Thomson J, Butts B, Camara S, Rasnick E, Brokamp C, Heyd C, et al. Neighborhood Socioeconomic Deprivation and Health Care Utilization of Medically Complex Children. Pediatrics. 2022;149(4):1-9.
68. Vance AJ, Pan W, Malcolm WH, Brandon DH. Development of parenting self-efficacy in mothers of high-risk infants. Early human development. 2020;141:104946.
69. Watkinson MD, Ehlenbach M, Chung PJ, Kelly M, Werner N, Jolliff A, et al. Interventions in the Home and Community for Medically Complex Children: A Systematic Review. Pediatrics. 2023.
70. Yamada H, Ohno K, Shiota M, Togawa M, Utsunomiya Y, Akaboshi S, et al. Prevalence and clinical characteristics of children with medical complexity in Tottori Prefecture, Japan: A population-based longitudinal study. Brain & development. 2020;42(10):747-55.
71. Yu J, Cook S, Imming C, Knezevich L, Ray K, Houtrow A, et al. A Qualitative Study of Family Caregiver Perceptions of High-Quality Care at a Pediatric Complex Care Center. Acad Pediatr. 2021:107-15.
72. Yu JA, Henderson C, Cook S, Ray K. Family Caregivers of Children With Medical Complexity: Health-Related Quality of Life and Experiences of Care Coordination. Academic pediatrics. 2020;20(8):1116-23.
73. Yu JA, McKernan G, Hagerman T, Schenker Y, Houtrow A. Most Children With Medical Complexity Do Not Receive Care in Well-Functioning Health Care Systems. Hospital pediatrics. 2021;11(2):183-91.
74. Yu JA, McKernan G, Hagerman T, Schenker Y, Houtrow A. Identifying children with medical complexity from the national survey of children s health combined 2016 17 data set. Hospital Pediatrics. 2021;11(2):192-7.
75. Barreda CB, Ehlenbach ML, Nackers A, Kelly MM, Shadman KA, Sklansky DJ, et al. Complex Care Program Enrollment and Change in ED and Hospital Visits from Medical Device Complications. Pediatric quality & safety. 2021;6(5):e450.

76. Barton HJ, Coller RJ, Loganathar S, Singhe N, Ehlenbach ML, Katz B, et al. Medical Device Workarounds in Providing Care for Children With Medical Complexity in the Home. Pediatrics. 2021;147(5):1-10.
77. Beauchamp-Walters J, Aleti G, Herrera L, Debelius J, Lima N, Dalal P, et al. Impact of exclusive enteral nutrition on the gut microbiome of children with medical complexity. JPEN Journal of parenteral and enteral nutrition. 2023;47(1):77-86.
78. Blaine K, Wright J, Pinkham A, O'Neill M, Wilkerson S, Rogers J, et al. Medication Order Errors at Hospital Admission Among Children With Medical Complexity. Journal of patient safety. 2022;18(1):e156-e62.
79. Caggiano S, Pavone M, Cherchi C, Paglietti MG, Schiavino A, Petreschi F, et al. Children with medical complexity and paediatric palliative care: data by a respiratory intermediate care unit. Pediatric pulmonology. 2022:918-26.
80. Conkol KJ, Martinez-Strengel A, Coller RJ, Bergman DA, Whelan E-M. Pediatric Hospitalists' Lessons Learned From an Innovation Award to Improve Care for Children With Medical Complexity. Hospital pediatrics. 2020;10(8):694-701.
81. Frush JM, Ming DY, Crego N, Paden ME, Jones-Hepler B, Misiewicz R, et al. Caregiver Perspectives on Telemedicine for Postdischarge Care for Children With Medical Complexity: A Qualitative Study. Journal of pediatric health care. 2023;37(4):356-363.
82. Galligan MM, Hogan AK. The Goldilocks problem: Healthcare delivery models for children with medical complexity. Current problems in pediatric and adolescent health care. 2021;51(12):101127.
83. Khan A, Baird J. Family Safety Reporting in Medically Complex Children: Parent, Staff, and Leader Perspectives. Pediatrics. 2022;149(6):51-63.
84. Lail J, Fields E, Paolella A, Schoettker PJ. Primary Care Quality Improvement Metrics and National Committee on Quality Assurance Medical Home Recognition for Children With Medical Complexity. Pediatric quality & safety. 2019;4(6):e231.
85. Ann de Banate M, Maypole J, Sadof M. Care coordination for children with medical complexity. Current Opinion in Pediatrics. 2019;31(4):575-82.
86. Asan O, Elkefi S, Clouser KN, Percy S. Using health information technology to support the needs of Children with Medical Complexity: Mapping review of consumer informatics applications. Frontiers in digital health. 2022;4:992838.
87. Azzopardi C, Cohen E, Pepin K, Netten K, Birken C, Madigan S. Child Welfare System Involvement Among Children With Medical Complexity. Child maltreatment. 2022;27(2):257-66.
88. Barton HJ, Pflaster E, Loganathar S, Werner A, Tarfa A, Wilkins D, et al. What makes a home? Designing home personas to represent the homes of families caring for children with medical complexity. Applied ergonomics. 2023;106:103900.
89. Baumbusch J, Lloyd JEV, Lamden-Bennett SR, Ou C. The unintended consequences of COVID-19 public health measures on health care for children with medical complexity. Child: care, health and development. 2022;48(6):970-8.
90. Bayer ND, Hongyue W, Yu JA, Kuo DZ, Halterman JS, Yue L. A National Mental Health Profile of Parents of Children With Medical Complexity. Pediatrics. 2021;148(2):1-10.

91. Berry JG, Goodman DM, Coller RJ, Agrawal R, Kuo DZ, Cohen E, et al. Association of Home Respiratory Equipment and Supply Use with Health Care Resource Utilization in Children. The Journal of pediatrics. 2019;207:169-75.e2.
92. Berry JG, Johnson C, Crofton C, Staffa SJ, DiTillio M, Leahy I, et al. Predicting Postoperative Physiologic Decline After Surgery. Pediatrics. 2019;143(4):1-11.
93. Bird M, Carter N, Lim A, Kazmie N, Fajardo C, Reaume S, et al. A Novel Hospital-to-Home System for Children With Medical Complexities: Usability Testing Study. JMIR formative research. 2022;6(8):e34572.
94. Black L, Shaunfield S, Labellarte PH, Gaebler-Spira D, Foster CC. Physical and Environmental Barriers to Mobility and Participation in Children With Medical Complexity: A Qualitative Study. Clinical pediatrics. 2022;61(10):717-26.
95. Black L, Shaunfield S, Labellarte PH, Gaebler-Spira D, Foster CC. Physical and Environmental Barriers to Mobility and Participation in Children With Medical Complexity: A Qualitative Study. Clinical pediatrics. 2022;61(10):717-26.
96. Buchanan F, Lai C, Cohen E, Milo-Manson G, Shachak A. Decision-making for Parents of Children With Medical Complexities: Activity Theory Analysis. Journal of participatory medicine. 2022;14(1):e31699.
97. Buser S, Brandenberger J, Gmunder M, Pohl C, Ritz N. Asylum-Seeking Children with Medical Complexity and Rare Diseases in a Tertiary Hospital in Switzerland. Journal of immigrant and minority health. 2021;23(4):669-79.
98. Cady R, Bushaw A, Davis H, Mills J, Thomasson D. Care coordination for children with medical complexity. The Nurse practitioner. 2020;45(6):11-7.
99. Caldarelli V, Porcaro F, Filippo PD, Attanasi M, Fainardi V, Gallucci M, et al. Long-Term Ventilation in Children with Medical Complexity: A Challenging Issue. Children (Basel, Switzerland). 2022;9(11):1700.
100. Cardenas A, Esser K, Wright E, Netten K, Edwards A, Rose J, et al. Caring for the Caregiver (C4C): An Integrated Stepped Care Model for Caregivers of Children With Medical Complexity. Acad Pediatr. 2023;23(2):236-43.
101. Coller RJ, Kelly MM, Howell KD, Warner G, Butteris SM, Ehlenbach ML, et al. In-Home COVID-19 Testing for Children With Medical Complexity: Feasibility and Association With School Attendance and Safety Perceptions. American journal of public health. 2022;112:S878-S82.
102. Costain G, Walker S, Marano M, Veenma D, Snell M, Curtis M, et al. Genome Sequencing as a Diagnostic Test in Children With Unexplained Medical Complexity. JAMA Network Open. 2020;3(9):e2018109-e.
103. Curran JA, Breneol S, Vine J. Improving transitions in care for children with complex and medically fragile needs: a mixed methods study. BMC pediatrics. 2020;20(1):219.
104. D'Aprano A, Gibb S, Riess S, Cooper M, Mountford N, Meehan E. Important components of a programme for children with medical complexity: An Australian perspective. Child: care, health and development. 2020;46(1):90-103.
105. Desai AD, Wang G, Wignall J, Kinard D, Singh V, Adams S, et al. User-centered design of a longitudinal care plan for children with medical complexity. Journal of the American Medical Informatics Association : JAMIA. 2020;27(12):1860-70.
106. DiDomizio PG, Millar MM, Olson L, Murphy N, Moore D. Palliative Care Needs Assessment for Pediatric Complex Care Providers. Journal of pain and symptom management. 2023;65(2):73-80.

107. Gall VN, Buchhalter J, Antonelli RC, Richard C, Yohemas M, Lachuk G, et al. Improving care for families and children with neurodevelopmental disorders and co-occurring chronic health conditions using a care coordination intervention. Journal of Developmental and Behavioral Pediatrics. 2022;43(8):444-53.
108. Gallo M, Agostiniani R, Pintus R, Fanos V. The child with medical complexity. Italian journal of pediatrics. 2021;47(1):1.
109. Genna C, Thekkan KR, Geremia C, Di Furia M, Campana A, Dall'Oglio I, et al. Parents' process of recognition and response to clinical deterioration of their children with medical complexity at home: A grounded theory. J Clin Nurs. 2023;32(15-16):4677-4693.
110. Hannan KE, Bourque SL, Palmer C, Tong S, Hwang SS. Prevalence and Predictors of Medical Complexity in a National Sample of VLBW Infants. Hospital pediatrics. 2021;11(5):525-35.
111. Haque B, Khan T, Ushcatz I, Curtis M, Pan A, Wu W, et al. Contemporary aetiologies of medical complexity in children: A cohort study. Archives of Disease in Childhood. 2022;108(2):147-9.
112. Hofmann M, Yonkaitis C. Lessons Learned from Caregivers of Children with Medical Complexity: Implications for Policy & Providers. Home healthcare now. 2022;40(4):196-201.
113. Hsu NM, Morris K, Banaag A, Koehlmoos TP. TRICARE Extended Care Health Option Program: Prevalence of pediatric ECHO enrollees and healthcare service utilization in the Military Health System. Disability and health journal. 2023:101451.
114. Jasek EE. Identifying solutions to address issues of safety in home care environments for children and youth. Dissertation Abstracts International: Section B: The Sciences and Engineering. 2022;83(11).
115. Lawrence PR, Feinberg I, Spratling R. The Relationship of Parental Health Literacy to Health Outcomes of Children with Medical Complexity. Journal of pediatric nursing. 2021;60:65-70.
116. Leary JC, Krcmar R, Yoon GH, Freund KM, LeClair AM. Parent Perspectives During Hospital Readmissions for Children With Medical Complexity: A Qualitative Study. Hospital pediatrics. 2020;10(3):222-9.
117. Lee W, Luca S, Costain G, Snell M, Marano M, Curtis M, et al. Genome sequencing among children with medical complexity: What constitutes value from parents' perspective? Journal of Genetic Counseling. 2022;31(2):523-533.
118. LeGrow K, Cohen E, Espin S. Relational Aspects of Parent and Home Health Care Provider Care Practices for Children With Complex Care Needs Receiving Health Care Services in the Home: A Narrative Review. Acad Pediatr. 2022;22(2):196-202.
119. Lim A, Butt ML, Dix J, Elliott L, Paes B. Respiratory syncytial virus (RSV) infection in children with medical complexity. European journal of clinical microbiology & infectious diseases : official publication of the European Society of Clinical Microbiology. 2019;38(1):171-6.
120. Lin JL, Huber B, Amir O, Gehrmann S, Ramirez KS, Ochoa KM, et al. Barriers and Facilitators to the Implementation of Family-Centered Technology in Complex Care: Feasibility Study. Journal of medical Internet research. 2022;24(8):e30902.

121. Lord S, Moore C, Beatty M, Cohen E, Rapoport A, Hellmann J, et al. Assessment of Bereaved Caregiver Experiences of Advance Care Planning for Children With Medical Complexity. JAMA network open. 2020;3(7):e2010337.
122. Matsuzawa A, Shiroki Y, Arai J, Hirasawa A. Care coordination for children with medical complexity in Japan: Caregivers' perspectives. Child: care, health and development. 2020;46(4):436-44.
123. McKenzie K, Dudevich A, Costante A, Chen X-K, Foebel AD. How Children and Youth with Medical Complexity Use Hospital and Emergency Department Care across Canada. Healthcare quarterly (Toronto, Ont). 2021;24(1):10-3.
124. Mercer AN, Mauskar S, Baird J, Berry J, Chieco D, Copp K, et al. Family Safety Reporting in Hospitalized Children With Medical Complexity. Pediatrics. 2022;150(2):e2021055098.
125. Ming DY, Jackson GL, Sperling J, Gray M, Wyman Roth N, Spears T, et al. Mobile Complex Care Plans to Enhance Parental Engagement for Children With Medical Complexity. Clinical pediatrics. 2019;58(1):34-41.
126. Ming DY, Jones KA, Sainz E, Tkach H, Stewart A, Cram A, et al. Feasibility of implementing systematic social needs assessment for children with medical complexity. Implementation science communications. 2021;2(1):130.
127. Ming DY, Jones KA, White MJ, Pritchard JE, Hammill BG, Bush C, et al. Healthcare Utilization for Medicaid-Insured Children with Medical Complexity: Differences by Sociodemographic Characteristics. Maternal and child health journal. 2022;26(12):2407-18.
128. Ming DY, Li T, Ross MH, Frush J, He J, Goldstein BA, et al. Feasibility of Post-hospitalization Telemedicine Video Visits for Children With Medical Complexity. Journal of pediatric health care : official publication of National Association of Pediatric Nurse Associates & Practitioners. 2022;36(2):e22-e35.
129. Moyes A, Abbott T, Baker S, Reid C, Thorne R, Morelius E. A parent first: Exploring the support needs of parents caring for a child with medical complexity in Australia. Journal of pediatric nursing. 2022;67:e48-e57.
130. Orkin J, Beaune L, Moore C, Weiser N, Arje D, Rapoport A, et al. Toward an understanding of advance care planning in children with medical complexity. Pediatrics. 2020;145(3):e20192241.
131. Peinado Fabregat MI, Saynina O, Sanders LM. Obesity and Overweight Among Children With Medical Complexity. Pediatrics. 2023;151(1):e2022058687.
132. Pezeshkpour P, Armstrong N, Mahant S, Muthusami P, Amaral J, Parra D, et al. Evaluation of the experience with implanted venous port-a-caths in children with medical complexity and neurologic impairment. Pediatric Radiology. 2019;49:S195.
133. Plews-Ogan J, Babbar A, Keim-Malpass J. Compassion and connectedness as motivational drivers in the care of children with medical complexity. Journal of pediatric rehabilitation medicine. 2019;12(3):279-84.
134. Prieto V, Rozmus C, Cohen E, LoBiondo-Wood G. Caregiver Burden, Caregiving Satisfaction, and Health-Related Quality of Life Among Caregivers of Children with Medical Complexity. Pediatric Nursing. 2022;48(3):111-21.
135. Pulcini CD, Coller RJ, Houtrow AJ, Belardo Z, Zorc JJ. Preventing Emergency Department Visits for Children With Medical Complexity Through Ambulatory Care: A Systematic Review. Academic pediatrics. 2021;21(4):605-16.

136. Raisanen JC, Ruth A, Donohue PK, Detwiler K, Fratantoni K, Huff SM, et al. Paying for Pediatric Home Health Care: How Families of Children With Medical Complexity Navigate Gaps in Coverage. Family & community health. 2021;44(3):184-93.
137. Rasooly IR, Shults J, Guevara JP, Feudtner C. State Variation in Posthospital Home Nursing for Commercially Insured Medically Complex Children. Pediatrics. 2020;146(2):1-8.
138. Rogers J, Reed MP, Blaine K, Manning H. Children with medical complexity: A concept analysis. Nursing forum. 2021;56(3):676-83.
139. Seltzer R, Raisanen JC, Silva TD, Donohue PK, Williams EP, Shepard J, et al. Medical Decision-Making in Foster Care: Considerations for the Care of Children with Medical Complexity. Academic pediatrics. 2019:333-40.
140. Sidra M, Sebastianski M, Ohinmaa A, Rahman S. Reported costs of children with medical complexity-A systematic review. Journal of child health care. 2024 Jun;28(2):377-401.
141. Sonsteng-Person M, Garcia-Perez J, Copeland V, Lievano-Karim L, Abrams D, Jarman B, et al. "What I Would Do to Take Away Your Pain": A Photovoice Project Conducted by Mothers of Children With Medical Complexity. Qualitative health research. 2023;33(3):204-19.
142. Teicher J, Moore C, Esser K, Weiser N, Arje D, Cohen E, et al. The Experience of Parental Caregiving for Children With Medical Complexity. Clinical pediatrics. 2023:62(6):633-644.
143. Valdez RS, Lunsford C, Bae J, Letzkus LC, Keim-Malpass J. Self-Management Characterization for Families of Children With Medical Complexity and Their Social Networks: Protocol for a Qualitative Assessment. JMIR research protocols. 2020;9(1):e14810.
144. van der Perk C-J, van de Riet L, Alsem M, van Goudoever JB, Maaskant J. Prognostic factors influencing parental empowerment after discharge of their hospitalized child: A cross-sectional study. Journal of pediatric nursing. 2022;66:e145-e51.
145. Verduci E, Salvatore S, Bresesti I, Di Profio E, Pendezza E, Bosetti A, et al. Semi-Elemental and Elemental Formulas for Enteral Nutrition in Infants and Children with Medical Complexity-Thinking about Cow's Milk Allergy and Beyond. Nutrients. 2021;13(12):4230.
146. Williams LJ, Waller K, Chenoweth RP, Ersig AL. Stakeholder perspectives: Communication, care coordination, and transitions in care for children with medical complexity. Journal for specialists in pediatric nursing : JSPN. 2021;26(1):e12314.
147. Xu WY, Li Y, Song C, Bose-Brill S, Retchin SM. Out-of-Network Care in Commercially Insured Pediatric Patients According to Medical Complexity. Med Care. 2022;60(5):375-80.
148. Burrell M, Ciccarelli M. Identifying Children With Medical Complexity for Care Coordination in Primary Care Settings. Clinical pediatrics. 2023 Jul;62(7):781-785.
149. Gold JM, Chadwick W, Gustafson M, Valenzuela LF, Mello A, Nasr A. Parent perceptions and experiences regarding medication education at time of hospital discharge for children with medical complexity. Hospital Pediatrics. 2020;10(8):679-86.

150. Verma R, Mehdian Y, Sheth N, Netten K, Vinette J, Edwards A, et al. Screening for caregiver psychosocial risk in children with medical complexity: a cross-sectional study. BMJ paediatrics open. 2020;4(1):e000671.
151. Curfman AL, Haycraft M, McSwain SD, Dooley M, Simpson KN. Implementation and Evaluation of a Wraparound Virtual Care Program for Children with Medical Complexity. Telemedicine journal and e-health. 2023;29(6):947-953.
152. Foster CC, Fuentes MM, Wadlington LA, Jacob-Files E, Desai AD, Simon TD, et al. Caregiver and Provider Experiences of Home Healthcare Quality for Children With Medical Complexity. Home healthcare now. 2020;38(3):138-46.
153. Heneghan JA, Goodman DM, Ramgopal S. Demographic and Clinical Differences Between Applied Definitions of Medical Complexity. Hospital pediatrics. 2022;12(7):654-63.
154. Nkoy F, Stone B, Sheng X, Murphy N. High Parental Concern in Children With Medical Complexity: An Early Indicator of Illness. Hospital pediatrics. 2023;13(3):250-7.
155. Leyenaar JK, Schaefer AP, Freyleue SD, Austin AM, Simon TD, Van Cleave J, et al. Prevalence of Children With Medical Complexity and Associations With Health Care Utilization and In-Hospital Mortality. JAMA pediatrics. 2022;176(6):e220687.
156. Marquez C, Thompson R, Feinstein JA, Orth LE. Identifying opportunities for pediatric medication therapy management in children with medical complexity. Journal of the American Pharmacists Association. 2022;62(5):1587-95.e3.
157. Donnelly S, Shaw E, Timoney P, Foca M, Hametz P. Parents' Assessment of an Advanced-Practice Nurse and Care Coordination Assistant Model Medical Care Coordination Program for Children With Medical Complexity. Journal of pediatric health care. 2020;34(4):325-32.
158. Wang G, Wignall J, Kinard D, Singh V, Foster C, Adams S, et al. An implementation model for managing cloud-based longitudinal care plans for children with medical complexity. Journal of the American Medical Informatics Association. 2021;28(1):23-32.
159. Jolliff A, Werner NE, Barton HJ, Howell KD, Kelly MM, Morgen M, et al. Caregiver perceptions of in-home COVID-19 testing for children with medical complexity: a qualitative study. BMC pediatrics. 2022;22(1):533.
160. Keim-Malpass J, Constantoulakis L, Letzkus LC. Variability In States' Coverage Of Children With Medical Complexity Through Home And Community-Based Services Waivers. Health affairs (Project Hope). 2019;38(9):1484-90.
161. Keim-Malpass J, Cozad MJ, Svynarenko R, Mack JW, Lindley LC. Medical complexity and concurrent hospice care: A national study of Medicaid children from 2011 to 2013. Journal for specialists in pediatric nursing. 2021;26(4):e12333.
162. Jia Lu Lilian LIN, Quartaron S, Aidaru N, Chan CY, Hubbert J, Orkin J, et al. Process Evaluation of a Hub-and-Spoke Model to Deliver Coordinated Care for Children with Medical Complexity across Ontario: Facilitators, Barriers and Lessons Learned. Healthcare Policy. 2021;17(1):104-22.
163. Jonas D, Scanlon C, Bogetz JF. Parental Decision-Making for Children with Medical Complexity: An Integrated Literature Review. Journal of pain and symptom management. 2021:e111-e23.

164. Kelly MM, DeMuri GP, Barton HJ, Nacht CL, Butteris SM, Katz B, et al. Priorities for Safer In-Person School for Children With Medical Complexity During COVID-19. Pediatrics. 2022;149(3):1-11.
165. Kemp KA, Fairie P, Steele B, McNeil D, Kromm S, Johnson D, et al. The Experience of Parents of Hospitalized Children Living With Medical Complexity. Hospital pediatrics. 2021;11(10):1065-72.
166. Coller RJ, Komatz K. Children with Medical Complexity and Neglect: Attention Needed. Journal of child & adolescent trauma. 2020;13(3):293-8.
167. Care coordination for children with medical complexity and caregiver empowerment in the process: A literature review. Journal for Specialists in Pediatric Nursing. 2022;27(3):1-12.
168. Blackmer AB, Fox D, Arendt D, Phillips K, Feinstein JA. Perceived Versus Demonstrated Understanding of the Complex Medications of Medically Complex Children. The journal of pediatric pharmacology and therapeutics. 2021;26(1):62-72.
169. Boss RD, Henderson CM, Weiss EM, Falck A, Madrigal V, Shapiro MC, et al. The Changing Landscape in Pediatric Hospitals: A Multicenter Study of How Pediatric Chronic Critical Illness Impacts NICU Throughput. American journal of perinatology. 2022;39(6):646-51.
170. Carrilero N, Dalmau-Bueno A, Garcia-Altes A. Comorbidity patterns and socioeconomic inequalities in children under 15 with medical complexity: a population-based study. BMC pediatrics. 2020;20(1):358.
171. Brand EAJ, Shaw M, Galo J. Implementing Spiritual Care in the Pediatric Complex Care Clinic. The journal of pastoral care & counseling. 2023;77(1):27-33.
172. Mantler T, Jackson KT, Baer J, White J, Ache B, Shillington K, et al. Changes in Care-A Systematic Scoping Review of Transitions for Children with Medical Complexities. Current pediatric reviews. 2020;16(3):165-75.
173. Sprecher E, Toomey S, Epee-Bounya A, Hernandez B, Le T, Conroy K. Randomized Controlled Trial of Health Coaching for Parents of Children With Medical Complexity. Acad Pediatr. 2022;22(8):1482-8.
174. Werner NE, Fleischman A, Warner G, Barton HJ, Kelly MM, Ehlenbach ML, et al. Feasibility Testing of Tubes@HOME: A Mobile Application to Support Family-Delivered Enteral Care. Hospital pediatrics. 2022;12(7):663-73.
175. Driansky A, Pilapil M, Bianco K, Steinway C, Feigenbaum S, Yang A, et al. Caring for Children With Medical Complexity in the Early COVID-19 Pandemic in an Ambulatory Primary Care Setting. Frontiers in pediatrics. 2022;10:813393.
176. Sobush KT. Principles of Managing Children with Medical Complexity and a New Delivery Model. Missouri medicine. 2019;116(2):134-9.
177. Boss RD, Raisanen JC, Detwiler K, Fratantoni K, Huff SM, Neubauer K, et al. Lived Experience of Pediatric Home Health Care Among Families of Children With Medical Complexity. Clinical pediatrics. 2019:9922819894006.
178. Bleazard M. Compassion Fatigue in Nurses Caring for Medically Complex Children. Journal of hospice and palliative nursing. 2020;22(6):473-8.
179. Bleazard MT. Differentiating Paroxysmal Sympathetic Hyperactivity from Seizures in Medically Complex Children. Pediatric Nursing. 2019;45(2):67-74.
180. Kasparian AM, Badawy SM. Utility of Fitbit devices among children and adolescents with chronic health conditions: a scoping review. mHealth. 2022;8:26.

181. Penela-Sanchez D, Ricart S, Vidiella N, Garcia-Garcia JJ. A study of paediatric patients with complex chronic conditions admitted to a paediatric department over a 12 month period. An Pediatr (Engl Ed). 2021;95(4):233-239.
182. Perros I, Papalexakis EE, Vuduc R, Searles E, Sun J. Temporal phenotyping of medically complex children via PARAFAC2 tensor factorization. Journal of biomedical informatics. 2019;93:103125.
183. Feehan K, Kehinde F, Sachs K, Mossabeb R, Berhane Z, Pachter LM, et al. Development of a Multidisciplinary Medical Home Program for NICU Graduates. Maternal and child health journal. 2020;24(1):11-21.
184. Kieran E, Sara R, Claydon J, Hait V, de Salaberry J, Osiovich H, et al. Outcomes of Neonates With Complex Medical Needs. Advances in neonatal care : official journal of the National Association of Neonatal Nurses. 2019;19(4):275-84.
185. Vance AJ. Development of parental confidence among parents of infants with medical complexity. Dissertation Abstracts International: Section B: The Sciences and Engineering. 2019;80(11).
186. Vance AJ, Knafl K, Brandon DH. Patterns of Parenting Confidence Among Infants With Medical Complexity: A Mixed-Methods Analysis. Advances in neonatal care : official journal of the National Association of Neonatal Nurses. 2021;21(2):160-8.
187. Lakshmanan A, Kubicek K, Williams R, Robles M, Vanderbilt DL, Mirzaian CB, et al. Viewpoints from families for improving transition from NICU-to-home for infants with medical complexity at a safety net hospital: a qualitative study. BMC pediatrics. 2019;19(1):223.

Online Resource 4B: Reference list Children and Youth with Special Health Care Needs (CYSHCN)

**Definition CYSHCN (n=100)**	
Definition McPherson et al. (1998)	(188-275)
**Studies using other term patient category with definition McPherson et al. (n=12):**	
Pediatric complex chronic care patients	(276)
Children with complex care needs	(277-282)
Children with complex health needs	(283-285)
Children with complex healthcare needs	(286, 287)

188. Abdullah ZA, Aziz KF. Parents' perception of rehabilitation services for their children with special healthcare needs in helena center in Erbil city. Indian Journal of Forensic Medicine and Toxicology. 2020;14(3):825-31.
189. Arrue AM, Hokerberg YHM, Jantsch LB, da Gama SGN, de Oliveira RdVC, Okido ACC, et al. Prevalence of children with special healthcare needs: An epidemiological survey in Brazil. Journal of pediatric nursing. 2022;67:95-101.
190. Atilgan ED, Tuncer A. The effects of breathing exercises in mothers of children with special health care needs:A randomized controlled trial. Journal of back and musculoskeletal rehabilitation. 2021;34(5):795-804.
191. Batista Medeiros JP, Tatsch Neves E, Vasconcelos Pitombeira MG, Vieira Figueiredo S, Barbosa Campos D, Verde Gomes IL. Continuity of care for children with special healthcare needs during the COVID-19 pandemic. Revista Brasileira de Enfermagem. 2022;75(2):1-8.
192. Bravo LG, Ahmed C, Choi K. Addressing Social Context in Adverse Childhood Experience Screening Policy: Implications for Children With Special Health Care Needs. Journal of pediatric health care. 2023;37(2):213-6.
193. Cohen E, Quartarone S, Orkin J, Moretti ME, Emdin A, Guttmann A, et al. Effectiveness of Structured Care Coordination for Children With Medical Complexity: The Complex Care for Kids Ontario (CCKO) Randomized Clinical Trial. JAMA Pediatrics 2023;177(5):461-471.
194. Corvey KJ. Employer sponsored health insurance and children with special health care needs. Dissertation Abstracts International: Section B: The Sciences and Engineering. 2020;81(12).
195. Dembo RS, LaFleur J, Akobirshoev I, Dooley DP, Batra N, Mitra M. Racial/ethnic health disparities among children with special health care needs in Boston, Massachusetts. Disability and health journal. 2022;15(3):101316.
196. Fuller AE, Brown NM, Grado L, Oyeku SO, Gross RS. Material Hardships and Health Care Utilization Among Low-Income Children with Special Health Care Needs. Academic pediatrics. 2019;19(7):733-9.
197. Ghandour RM, Hirai AH, Kenney MK. Children and Youth With Special Health Care Needs: A Profile. Pediatrics. 2022;149:S731-S47.
198. Gigli KH, Graaf G. Changes in Use and Access to Care for Children and Youth With Special Health Care Needs During the COVID-19 Pandemic. Journal of pediatric health care. 2023;37(2):185-92.

199. Graaf G, Annis I, Martinez R, Thomas KC. Predictors of Unmet Family Support Service Needs in Families of Children with Special Health Care Needs. Maternal and child health journal. 2021;25(8):1274-84.
200. Graaf G, Snowden L. Public Health Coverage and Access to Mental Health Care for Youth with Complex Behavioral Healthcare Needs. Administration and policy in mental health. 2020;47(3):395-409.
201. Kohl SE, Barnett ED. What do we know about travel for children with special health care needs? A review of the literature. Travel medicine and infectious disease. 2020;34:101438.
202. Kuo DZ, Comeau M, Perrin JM, Coleman C, White P, Lerner C, et al. Moving From Spending to Investment: A Research Agenda for Improving Health Care Financing for Children and Youth With Special Health Care Needs. Acad Pediatr. 2022;22(2):S47-S53.
203. Kuo DZ, Rodgers RC, Beers NS, McLellan SE, Nguyen TK. Access to Services for Children and Youth With Special Health Care Needs and Their Families: Concepts and Considerations for an Integrated Systems Redesign. Pediatrics. 2022;149:S775-S81.
204. Li M, Ji C, Wang B, Yao D, Wang X, Zeng Y, et al. Incomplete Vaccination Among Children With Special Health Care Needs in Zhejiang, China: Analysis of Retrospective Data. Frontiers in pediatrics. 2019;7:173.
205. Lindly OJ, Martin AJ, Lally K. A Profile of Care Coordination, Missed School Days, and Unmet Needs Among Oregon Children with Special Health Care Needs with Behavioral and Mental Health Conditions. Community mental health journal. 2020;56(8):1571-80.
206. Liu S, Lombardi J, Fisher PA. The COVID-19 Pandemic Impact on Households of Young Children With Special Healthcare Needs. Journal of pediatric psychology. 2022;47(2):158-70.
207. Marcon SS, Dias BC, Neves ET, Marcheti MA, Lima RAG. (In)visibility of children with special health needs and their families in primary care. Revista brasileira de enfermagem. 2020;73:e20190071.
208. McKay S. Immigrant Children With Special Health Care Needs: A Review. Current problems in pediatric and adolescent health care. 2019;49(2):45-9.
209. Michaud M, Dietz IC. The effects of the SARS-CoV-2 pandemic on children and youth with special health care needs. Frontiers in pediatrics. 2022;10:1007770.
210. Min EE, Wheeler H, Hennessy C, Abebe E. Medication Management Strategies by Family Caregivers of Children with Special Health Care Needs. Child care health and development. 2023 Sep;49(5):925-932.
211. Moeenuddin Z, Kim-Kupfer C, Owchar E, Baker J, Duffield A, Santoro T. The Influence of Care Coordination on Patients With Special Health Care Needs in a Pediatric Residency Continuity Clinic. Global pediatric health. 2019;6:2333794X19848677.
212. Munambah N, Cordier R, Speyer R, Toto S, Ramugondo EL. A Systematic Review Comparing the Play Profiles of Children with Special Health Care Needs with Typically Developing Children. BioMed research international. 2020;2020:9582795.
213. O'Connor M, O'Connor E, Quach J, Vashishtha R, Goldfeld S. Trends in the prevalence and distribution of teacher-identified special health-care needs across

three successive population cohorts. Journal of paediatrics and child health. 2019;55(3):312-9.
214. Parmar A, Esser K, Barreira L, Miller D, Morinis L, Chong Y-Y, et al. Acceptance and Commitment Therapy for Children with Special Health Care Needs and Their Parents: A Systematic Review and Meta-Analysis. International journal of environmental research and public health. 2021;18(15):8205.
215. Parrish Ii RH, Casher D, van den Anker J, Benavides S. Creating a Pharmacotherapy Collaborative Practice Network to Manage Medications for Children and Youth: A Population Health Perspective. Children (Basel, Switzerland). 2019;6(4).
216. Passos dos Santos R, Gais Severo VR, Jaciara Kegler J, Bigolin Jantsch L, Cordeiro D, Tatsch Neves E. Perfil de crianças com necessidades especiais de saúde e seus cuidadores em um hospital de ensino. Ciencia, Cuidado e Saude. 2020;19:1-8.
217. Perez Jolles M, Zullig LL, Lee P-J, Kolhatkar G. Disparities in Shared Decision Making and Service Receipt Among Children With Special Health Care Needs and Developmental Delay: A National Survey Analysis. Journal of primary care & community health. 2020;11:2150132720924588.
218. Sellmaier C. Work, family, and community ecologies and their influence on work-life fit for fathers of children with special health care needs. Families in Society. 2019;100(1):68-79.
219. Sellmaier C. Physical and Mental Health of Mothers and Fathers Caring for Children with Special Health Care Needs: The Influence of Community Resources. Journal of Family Issues. 2022;43(11):2815-40.
220. Sellmaier C, Buckingham SR. 'I think sometimes that dads are kind of forgotten (...) so it's nice that we also get a voice.': Work-life experiences of employed US Fathers caring for a child with special health care needs. Community, Work & Family. 2021:Not specified.
221. Sezgin E, Noritz G, Lin S, Huang Y. Feasibility of a Voice-Enabled Medical Diary App (SpeakHealth) for Caregivers of Children With Special Health Care Needs and Health Care Providers: Mixed Methods Study. JMIR formative research. 2021;5(5):e25503.
222. Sezgin E, Oiler B, Abbott B, Noritz G, Huang Y. "Hey Siri, Help Me Take Care of My Child": A Feasibility Study With Caregivers of Children With Special Healthcare Needs Using Voice Interaction and Automatic Speech Recognition in Remote Care Management. Frontiers in public health. 2022;10:849322.
223. Smith SL, Aytur SA, Humphreys BP. Effects of Telehealth Parent Coaching in Supporting Family Participation, Cohesion, and Adaptability. OTJR Occupation, Participation and Health. 2022:24-34.
224. Sonik RA, Coleman-Jensen A, Parish SL. Household food insufficiency, health status and emergency healthcare utilisation among children with and without special healthcare needs. Public Health Nutrition. 2020;23(17):3204-10.
225. van Zwieten A, Teixeira-Pinto A, Lah S, Nassar N, Craig JC, Wong G. Special health care needs during childhood and academic achievement in secondary school. Child: care, health and development. 2022;48(2):311-23.
226. Wells R, Daniel P, Barger B, Rice CE, Bandlamudi M, Crimmins D. Impact of medical home-consistent care and child condition on select health, community, and family level outcomes among children with special health care needs. Children's Health Care. 2020:Not specified.

227. Balistreri KS. Food insufficiency and children with special healthcare needs. Public health. 2019;167:55-61.
228. Bird M, Li L, Ouellette C, Hopkins K, McGillion MH, Carter N. Use of Synchronous Digital Health Technologies for the Care of Children With Special Health Care Needs and Their Families: Scoping Review. JMIR pediatrics and parenting. 2019;2(2):e15106.
229. Bradshaw S, Bem D, Shaw K, Taylor B, Chiswell C, Salama M, et al. Improving health, wellbeing and parenting skills in parents of children with special health care needs and medical complexity - a scoping review. BMC pediatrics. 2019;19(1):301.
230. Brodie N, Bernstein B, McNesby F, Turchi R. The Experience of Latina Mothers of Children and Youth With Special Health Care Needs: A Qualitative Study. Global pediatric health. 2019;6:2333794X19851390.
231. Camelo Castillo W, Onasanya O, dosReis S, Hogans B, Reeves G. Caregiver Burden in Caregivers of Children With Special Health Care Needs and Association With Chronic Pain. Medical care. 2022;60(5):368-74.
232. Coller RJ, Berry JG, Kuo DZ, Kuhlthau K, Chung PJ, Perrin JM, et al. Health System Research Priorities for Children and Youth With Special Health Care Needs. Pediatrics. 2020;145(3):1-12.
233. Cosmo de Oliveira Carvalho C, Guilherme Pereira Pimentel T, Evangelista Cabral I. Child with special health needs at one hospital of the brazilian unified health system. Revista de Pesquisa: Cuidado e Fundamental. 2021;13(1):1296-302.
234. Costich MA, Peretz PJ, Davis JA, Stockwell MS, Matiz LA. Impact of a Community Health Worker Program to Support Caregivers of Children With Special Health Care Needs and Address Social Determinants of Health. Clinical pediatrics. 2019;58(11):1315-20.
235. da Silveira A, Santini Costenaro RG, Tatsch Neves E. Adolescents with special health care needs: challenges of school inclusion according to family members/caregivers' viewpoint. Revista de Pesquisa: Cuidado e Fundamental. 2020;12(1):1290-5.
236. de Oliveira Nörberg PK, Calcagno Gomes G, Rodrigues Costa A, Goulart Nobre CM, Avila Minasi AS, Quadros Alvarez S. The itinerary of families to obtain the diagnosis of the children with special healthcare needs. Ciencia, Cuidado e Saude. 2022;21:1-9.
237. Edwards L, Leafman JS. Perceptions of Gastrostomy Buttons Among Caregivers of Children With Special Health Care Needs. Journal of pediatric health care. 2019;33(3):270-9.
238. Foster CC, Chorniy A, Kwon S, Kan K, Heard-Garris N, Davis MM. Children With Special Health Care Needs and Forgone Family Employment. Pediatrics. 2021;148(3):1-11.
239. Fuller AE, Garg A, Brown NM, Tripodis Y, Oyeku SO, Gross RS. Relationships Between Material Hardship, Resilience, and Health Care Use. Pediatrics. 2020;145(2):1-8.
240. Graaf G, Baiden P, Boyd G, Keyes L. Barriers to Respite Care for Children with Special Health Care Needs. Journal of developmental and behavioral pediatrics. 2022;43(3):117-29.
241. Green C, Jung H-Y, Wu X, Abramson E, Walkup JT, Ford JS, et al. Do Children with Special Health Care Needs with Anxiety have Unmet Health Care Needs? An Analysis of a National Survey. Maternal and child health journal. 2019;23(9):1220-31.

242. Havinga J, Tumin D, Peedin L. Birth Weight Gradient in Parent-Reported Special Healthcare Needs among Children Born Preterm. The Journal of pediatrics. 2019:73-8.
243. Jetelina KK, Rodriguez P, Oke OK, Mathew MS, Schoppa S. Factors Influencing the Implementation of Social Determinants of Health Screening and Referral Processes in Pediatric Settings Serving Medically Complex Patients. Journal of Applied Research on Children. 2020;11(1):1-17.
244. Jewitt N, Orkin J, Cohen E, Narang I, Al-Saleh S, Amin R. The impact of polysomnograms and family-centred decision making in children with medical complexity. Paediatrics & child health. 2021;26(2):114-9.
245. Johnels L, Vehmas S, Wilder J. Musical interaction with children and young people with severe or profound intellectual and multiple disabilities: a scoping review. International Journal of Developmental Disabilities. 2021;69(4):487-504.
246. Johnston EE, Currie ER, Chen Y, Kent EE, Ornstein KA, Bhatia S, et al. Palliative Care Knowledge and Characteristics in Caregivers of Chronically Ill Children. Journal of Hospice & Palliative Nursing. 2020;22(6):456-64.
247. Lillvis DF, Sheehan KM, Yu J, Noyes K, Bass KD, Kuo DZ. Characterizing physical trauma in children and youth with special health care needs. The journal of trauma and acute care surgery. 2022;93(3):299-306.
248. Matiz LA, Kostacos C, Robbins-Milne L, Chang SJ, Rausch JC, Tariq A. Integrating Nurse Care Managers in the Medical Home of Children with Special Health Care needs to Improve their Care Coordination and Impact Health Care Utilization. Journal of pediatric nursing. 2021;59:32-6.
249. Matiz LA, Robbins-Milne L, Rausch JA. EMR Adaptations to Support the Identification and Risk Stratification of Children with Special Health Care Needs in the Medical Home. Maternal and child health journal. 2019;23(7):919-24.
250. Mattson G, Kuo DZ. Psychosocial Factors in Children and Youth With Special Health Care Needs and Their Families. Pediatrics. 2019;143(1):1-14.
251. Mirza M, Keating E, Krischer A, Pinto J. Care coordination Experiences of Low-Income Parents of Children and Youth with Special Health Care Needs: An Exploratory Study. Journal of health care for the poor and underserved. 2022;33(4):1925-48.
252. Monroy Tapiador MA, Climent Alcala FJ, Rodriguez Alonso A, Molina Amores C, Mellado Pena MJ, Calvo Rey C. Current situation of children with medical complexity: An experience between primary and hospital care. Atencion Primaria. 2021;53(6):102046.
253. Moretti A, Cianci P, De Paoli A, Meroni F, Taje S, Mariani M, et al. Burden of care in families of patients with rare genetic diseases: analysis of a large Italian cohort. European journal of medical genetics. 2021;64(7):104230.
254. Pankewicz A, Davis RK, Kim J, Antonelli R, Rosenberg H, Berhane Z, et al. Children With Special Needs: Social Determinants of Health and Care Coordination. Clinical pediatrics. 2020;59(13):1161-8.
255. Ranade-Kharkar P. Understanding information needs and designing tools for care coordination of children and youth with special health care needs (CYSHCN). Dissertation Abstracts International Section A: Humanities and Social Sciences. 2020;81(2):Not specified.

256. Roman SB, Dworkin PH, Dickinson P, Rogers SC. Analysis of Care Coordination Needs for Families of Children with Special Health Care Needs. Journal of developmental and behavioral pediatrics : JDBP. 2020;41(1):58-64.
257. Rose-Jacobs R, De Cuba SE, Bovell-Ammon A, Black MM, Coleman SM, Cutts D, et al. Housing instability among families with young children with special health care needs. Pediatrics. 2019;144(2):e20181704.
258. Rosenthal E, Franklin Gillette S, DuPaul GJ. Pediatric siblings of children with special health care needs: Well-being outcomes and the role of family resilience. Children's Health Care. 2021:452-65.
259. Stille CJ, Coller RJ, Shelton C, Wells N, Desmarais A, Berry JG. National Research Agenda on Health Systems for Children and Youth With Special Health Care Needs. Acad Pediatr. 2022;22(2):S1-S6.
260. Swann-Thomsen HE, Lindsay R, Rourk S, Hofacer R, Nguyen E. National Survey Data to Evaluate Case Management Services: A Systematic Review on Care Coordination Using the National Survey of Children With Special Health Care Needs. Professional case management. 2022;27(3):124-40.
261. Graaf G, Gigli K. Care coordination and unmet need for specialised health services among children with special healthcare needs in the USA: results from a cross-sectional analysis of the national survey of children with special healthcare needs. BMJ open. 2022;12(11):e063373.
262. Jin X, Wong CL, Li H, Chen J, Chong YY, Bai Y. Acceptance and Commitment Therapy for psychological and behavioural changes among parents of children with chronic health conditions: A systematic review. Journal of advanced nursing. 2021;77(7):3020-33.
263. McLellan SE, Mann MY, Scott JA, Brown TW. A Blueprint for Change: Guiding Principles for a System of Services for Children and Youth With Special Health Care Needs and Their Families. Pediatrics. 2022;149:S722-S30.
264. Moonpanane K, Kodyee S, Potjanamart C, Purkey E. Adjusting the family's life: A grounded theory of caring for children with special healthcare needs in rural areas, Thailand. PloS one. 2021;16(10):e0258664.
265. Nascimento Vasconcellos R, do Nascimento Souza MH, Medeiros da Nóbrega V, Collet N. A família da criança com necessidades especiais de saúde e suas relações sociais. Revista Brasileira de Enfermagem. 2022;75:1-9.
266. Pegorin TC, Furlan de Léo MM, Spiegelberg Zuge S, de Brum CN, da Rosa L, da Conceição VM. Quality of life and mental disorders in caregivers of children with special needs. Rev Rene. 2021;22(1):1-8.
267. Precce ML, Moraes JRMM, Pacheco STA, Silva LFD, Conceicao DSD, Rodrigues EDC. Educational demands of family members of children with special health care needs in the transition from hospital to home. Revista brasileira de enfermagem. 2020;73:e20190156.
268. Vasconcellos RN, Souza MHdN, Nobrega VMd, Collet N. The family of the child with special health care needs and their social relationships. Revista brasileira de enfermagem. 2022;75:e20210031.
269. Morton B, Damato EG, Ciccarelli MR, Currie J. Care Coordination for Children with Special Healthcare Needs Anticipating Transition: A Program Evaluation. Journal of pediatric nursing. 2021;61:7-14.

270. Casacio GDdM, Ferrari RAP, Zilly A, Silva RMMd. Therapeutic itinerary of children with special health care needs: analysis guided by care systems. Revista gaucha de enfermagem. 2022;43:e20210115.
271. Position Statement on Children and Youth with Special Health Care Needs: Key Issues on Care Coordination, Transitions, and Leadership. Journal of Pediatric Healthcare. 2021;35(3):317-9.
272. Silveira Ad, Huppes GM, Soster FF, Bueno TdV, Bartsch L, Mafalda MLD. Each child is a child: singularity of children with special health needs. Journal of Nursing & Health. 2021;11(3):1-12.
273. Tres DA, Martini RG, Toso BRGdO, Zanatta EA. Characterization of Home Care Services and care for children with special health care needs. Revista da Escola de Enfermagem da USP. 2022;56:e20220032.
274. Chisolm DJ, Keedy HE, Hart LC, Chavez LJ, Dolce M, Morack J, et al. Exploring Health Literacy, Transition Readiness, and Healthcare Utilization in Medicaid Chronically Ill Youth. The Journal of adolescent health. 2021;69(4):622-8.
275. Mihaila I, Berg K, Acharya K. Associations Between Participation and Mental Health for Vulnerable Youth with Special Healthcare Needs. Maternal and child health journal. 2021;25(9):1482-90.
276. Kobussen TA, Hansen G, Brockman RJ, Holt TR. Perspectives of Pediatric Providers on Patients With Complex Chronic Conditions: A Mixed-Methods Sequential Explanatory Study. Critical care nurse. 2020;40(5):e10-e7.
277. Birchley G, Thomas-Unsworth S, Mellor C, Baquedano M, Ingle S, Fraser J. Factors affecting decision-making in children with complex care needs: a consensus approach to develop best practice in a UK children's hospital. BMJ paediatrics open. 2022;6(1):e001589.
278. Brenner M, Greene J, Doyle C, Koletzko B, Del Torso S, Bambir I, et al. Increasing the Focus on Children's Complex and Integrated Care Needs: A Position Paper of the European Academy of Pediatrics. Frontiers in pediatrics. 2021;9:758415.
279. Brenner M, O'Shea MP, Larkin P, Berry J. Key constituents for integration of care for children assisted with long-term home ventilation: a European study. BMC pediatrics. 2020;20(1):71.
280. Cassidy L, Quirke MB, Alexander D, Greene J, Hill K, Connolly M, et al. Integrated care for children living with complex care needs: an evolutionary concept analysis. European journal of pediatrics. 2023;182(4):1517-1532.
281. Sur A, Paria A, Sivashankar S. Management pathway for infants requiring chronic care in neonatal units-a scoping review of practices. Eur J Pediatr. 2022;181(9):3235-42.
282. LeGrow K, Cohen E, Espin S. Mother-nurse decision making practices for children with complex health care needs receiving homecare services: A qualitative descriptive study. Child: care, health and development. 2022;48(4):605-12.
283. Grant M, McNeilly P. Children and young people's experiences of having a sibling with complex health needs: a literature review. Nursing Children & Young People. 2021;33(6):20-6.
284. Ronan S, Brown M, Marsh L. Parents' experiences of transition from hospital to home of a child with complex health needs: A systematic literature review. Journal of Clinical Nursing (John Wiley & Sons, Inc). 2020;29(17):3222-35.

285. Sandhu S, Ming DY, Crew C, Morreale MC, Cleveland S, Lail J, et al. Identifying Priorities to Improve the System of Care for Children With Complex Health Needs in North Carolina: Process and Outcomes of Systematic Stakeholder Engagement. Acad Pediatr. 2022;22(6):1041-8.
286. Brenner M, Doyle A, Begley T, Doyle C, Hill K, Murphy M. Enhancing care of children with complex healthcare needs: an improvement project in a community health organisation in Ireland. BMJ open quality. 2021;10(1):e001025.
287. Kelly KJ, Doucet S, Luke A, Azar R, Montelpare W. Exploring the Use of a Facebook-Based Support Group for Caregivers of Children and Youth With Complex Care Needs: Qualitative Descriptive Study. JMIR pediatrics and parenting. 2022;5(2):e33170.

Online Resource 4C: Reference list Chronic Complex Conditions (CCC)

**Definitions CCC (n=45)**	
Definition Feudtner et al. (2000)	(288-324)
**Studies using other term patient category with definition Feudtner et al. (n=6)**	
complex health conditions	(325)
children with complex medical needs	(326)
with life-limiting complex chronic conditions	(327)
pediatric home healthcare users	(328)
chronic complex health conditions	(329)
multiple complex chronic conditions	(330)
**Additional information**	
Mortality	(294)
High complex care	(291)
Neurological CCC	(288)
**Second definition**	
Definition Simon et al. (2010)	(331, 332)

288. Berry JG, Difazio RL, Melvin P, Glader L, Casto E, Shore BJ. Hospital resource use after hip reconstruction surgery in children with neurological complex chronic conditions. Developmental medicine and child neurology. 2020:204-10.

289. Fonseca R, Carvalho M, Querido A, Figueiredo MH, Bally J, Charepe Z. Therapeutic letters: A qualitative study exploring their influence on the hope of parents of children receiving pediatric palliative care in Portugal. Journal for specialists in pediatric nursing. 2021;26(3):e12325.

290. Antoon JW, Goldman JL, Lee BR. Severe Cutaneous Adverse Reactions: Comparing Outcomes in Children With and Without Complex Chronic Conditions. The journal of allergy and clinical immunology In practice. 2019:790-2.e3.

291. Arias Lopez MDP, Fernandez AL, Fiquepron K, Meregalli C, Ratto ME, Siaba Serrate A. Prevalence of Children With Complex Chronic Conditions in PICUs of Argentina: A Prospective Multicenter Study. Pediatric critical care medicine. 2019:E143-E51.

292. Bogetz JF, Revette A, DeCourcey D. Bereaved Parent Perspectives on the Benefits and Burdens of Technology Assistance among Children with Complex Chronic Conditions. Journal of palliative medicine. 2022;25(2):250-8.

293. Bogetz JF, Revette A, DeCourcey DD. Clinical Care Strategies That Support Parents of Children With Complex Chronic Conditions. Pediatric critical care medicine. 2021;22(7):595-602.

294. Bogetz JF, Revette A, Partin L, DeCourcey DD. Relationships and Resources Supporting Children With Serious Illness and Their Parents. Hospital pediatrics. 2022;12(9):832-42.

295. Bogetz JF, Revette A, Rosenberg AR, DeCourcey D. "I Could Never Prepare for Something Like the Death of My Own Child": Parental Perspectives on Preparedness at End of Life for Children With Complex Chronic Conditions. Journal of pain and symptom management. 2020;60(6):1154-62.e1.

296. Brown A, Quaile M, Morris H, Tumin D, Parker CL, Warren L, et al. Outpatient Follow-up Care After Hospital Discharge of Children With Complex Chronic Conditions at a Rural Tertiary Care Hospital. Clinical pediatrics. 2021;60(13):512-9.
297. DeCourcey DD, Silverman M, Oladunjoye A, Wolfe J. Advance Care Planning and Parent-Reported End-of-Life Outcomes in Children, Adolescents, and Young Adults With Complex Chronic Conditions. Critical care medicine. 2019;47(1):101-8.
298. Deming RS, Wolfe J, DeCourcey DD. Weighing Distress and Benefit: Understanding the Research Participation Experiences of Bereaved Parents of Children with Complex Chronic Conditions. Journal of pain and symptom management. 2020:39-.
299. Edwards JD, Goodman DM. The Child With Severe Chronic Illness in the ICU: A Concise Review. Critical care medicine. 2022;50(5):848-59.
300. Fornehed MLC, Svynarenko R, Keim-Malpass J, Cozad MJ, Qualls KA, Stone WL, et al. Comparison between Rural and Urban Appalachian Children in Hospice Care. Southern medical journal. 2022;115(3):192-7.
301. Friedel M, Gilson A, Bouckenaere D, Brichard B, Fonteyne C, Wojcik T, et al. Access to paediatric palliative care in children and adolescents with complex chronic conditions: a retrospective hospital-based study in Brussels, Belgium. BMJ paediatrics open. 2019;3(1):e000547.
302. Garcia-Fernandez de Villalta M, Mellado-Pena MJ, Delgado-Hierro A, Limia A, Climent Alcala FJ, Escosa-Garcia L, et al. Greater efforts are needed to ensure that children with complex medical conditions are properly vaccinated. Acta paediatrica (Oslo, Norway). 2019:422-3.
303. Harmon A, Jordan M, Platt A, Wilson J, Keith K, Chandrashekaran S, et al. Goal-Concordance in Children with Complex Chronic Conditions. The Journal of pediatrics. 2023;253:278-85.e4.
304. Houtrow AJ, Carle A, Perrin JM, Stein REK. Children With Special Health Care Needs on Supplemental Security Income for Disability Have More Health Impacts and Needs Than Other Children With Special Health Care Needs on Medicaid. Academic pediatrics. 2020;20(2):258-66.
305. Lindley LC, Cohrs AC, Keim-Malpass J, Leslie DL. Children Enrolled in Hospice Care Under Commercial Insurance: A Comparison of Different Age Groups. The American journal of hospice & palliative care. 2019;36(2):123-9.
306. Lindley LC, Cozad MJ, Fortney CA. Pediatric Complex Chronic Conditions: Evaluating Two Versions of the Classification System. Western journal of nursing research. 2020;42(6):454-61.
307. Lindley LC, Cozad MJ, Svynarenko R, Keim-Malpass J, Mack JW. A National Profile of Children Receiving Pediatric Concurrent Hospice Care, 2011 to 2013. Journal of hospice and palliative nursing. 2021;23(3):214-20.
308. Lindley LC, Fortney CA. Pediatric Complex Chronic Conditions: Does the Classification System Work for Infants? The American journal of hospice & palliative care. 2019;36(10):858-63.
309. Lindley LC, Fortney CA, Cozad MJ. Predictive Ability of an Illness Severity Measure: Implications for Nursing Research. Journal of nursing measurement. 2021;29(2):213-26.

310. Marcus KL, Kao P-C, Ma C, Wolfe J, DeCourcey DD. Symptoms and Suffering at End of Life for Children With Complex Chronic Conditions. Journal of pain and symptom management. 2022;63(1):88-97.
311. Markham JL, Richardson T, Teufel RJ, Hersh AL, DePorre A, Fleegler EW, et al. Impact of COVID-19 on Admissions and Outcomes for Children With Complex Chronic Conditions. Hospital pediatrics. 2022;12(4):337-53.
312. Pérez-Ardanaz B, José Peláez-Cantero M, Miguel Morales-Asencio J, Vellido-González C, Gómez-González A, León-Campos Á, et al. Socioeconomic Factors and Quality of Life Perceived by Parents and Children with Complex Chronic Conditions in Spain. Children (Basel). 2021;8(10):1-12.
313. Perez-Ardanaz B, Morales-Asencio JM, Pelaez-Cantero MJ, Garcia-Mayor S, Canca-Sanchez JC, Marti-Garcia C. Fatigue, quality of life and health resource utilisation in children with complex chronic diseases. Anales del Sistema Sanitario de Navarra. 2022;45(2):e1008.
314. Rafferty KA, Beck G, McGuire M. When Facing Hopeful and Hopeless Experiences: Using Snyder's Hope Theory to Understand Parents' Caregiving Experiences for Their Medically Complex Child. Journal of pediatric health care. 2020;34(6):542-9.
315. Rafferty KA, Beck GA. "You Are Not Alone": Advice Giving for Parents of Children Living with Complex Chronic Conditions. Health communication. 2020;35(11):1386-95.
316. Rafferty KA, Nemmers-Bello S, Tietz S, Lipovac M. Mothers' Support Preferences With Their Chronically Ill Child's Health Care Team. Western journal of nursing research. 2022;44(10):912-9.
317. Rupp Hanzen Andrades G, Abud Drumond Costa C, Crestani F, Tedesco Tonial C, Fiori H, Santos IS, et al. Association of nutritional status with clinical outcomes of critically ill pediatric patients with complex chronic conditions. Clinical nutrition (Edinburgh, Scotland). 2022;41(12):2786-91.
318. Silber JH, Rosenbaum PR, Pimentel SD, Calhoun S, Wang W, Sharpe JE, et al. Comparing Resource Use in Medical Admissions of Children With Complex Chronic Conditions. Medical care. 2019;57(8):615-24.
319. Verlaat CW, Wubben N, Visser IH, Hazelzet JA, van der Hoeven J, Lemson J, et al. Retrospective cohort study on factors associated with mortality in high-risk pediatric critical care patients in the Netherlands. BMC Pediatr. 2019;19(1):274.
320. White MJ, Sutton AG, Ritter V, Fine J, Chase L. Interfacility Transfers Among Patients With Complex Chronic Conditions. Hospital pediatrics. 2020;10(2):114-22.
321. Oztek Celebi FZ, Senel S. Patients with chronic conditions and their complex care needs in a tertiary care hospital. Archives de pediatrie : organe officiel de la Societe francaise de pediatrie. 2021;28(6):470-4.
322. Parker CL, Wall B, Tumin D, Stanley R, Warren L, Deal K, et al. Care Coordination Program for Children With Complex Chronic Conditions Discharged From a Rural Tertiary-Care Academic Medical Center. Hospital pediatrics. 2020;10(8):687-93.
323. Jenkins AM, Berry JG, Perrin JM, Kuhlthau K, Hall M, Dunbar P, et al. What Types of Hospitals Do Adolescents and Young Adults With Complex Chronic Conditions Use? Acad Pediatr. 2022;22(6):1033-40.

324. Vance AJ, Costa DK, Brandon DH. Parenting Self-Efficacy in Fathers of Medically Complex Infants: A Longitudinal Study. Journal of neonatal nursing. 2021;27(6):439-43.
325. Richard J, Azar R, Doucet S, Luke A. Pediatric Patient and Family Advisory Councils: A Guide to Their Development and Ongoing Implementation. Journal of patient experience. 2020;7(6):1476-81.
326. Brown CM, Williams DJ, Hall M, Freundlich KL, Johnson DP, Lind C, et al. Trends in Length of Stay and Readmissions in Children's Hospitals. Hospital pediatrics. 2021;11(6):554-62.
327. Nogueira A, Correia D, Loureiro M, Gomes B, Cancelinha C. The needs of children receiving end of life care and the impact of a paediatric palliative care team: a retrospective cohort study. European journal of pediatrics. 2022:525-31.
328. Sobotka SA, Hall DE, Thurm C, Gay J, Berry JG. Home Health Care Utilization in Children With Medicaid. Pediatrics. 2022;149(2):1-9.
329. Pinto M, Gomes R, Tanabe RF, Costa ACCD, Moreira MCN. Analysis of the cost of care for children and adolescents with medical complex chronic conditions. Ciencia & saude coletiva. 2019;24(11):4043-52.
330. Bjur KA, Wi C-I, Ryu E, Crow SS, King KS, Juhn YJ. Epidemiology of Children With Multiple Complex Chronic Conditions in a Mixed Urban-Rural US Community. Hospital pediatrics. 2019;9(4):281-90.
331. Agud M, de Medrano I, Mendez-Echevarria A, Sainz T, Roman F, Ruiz Carrascoso G, et al. Risk factors for antibiotic-resistant bacteria colonisation in children with chronic complex conditions. Scientific reports. 2022;12(1):7223.
332. Godoy-Molina E, Fernandez-Ferrandez T, Ruiz-Sanchez JM, Cordon-Martinez A, Perez-Frias J, Navas-Lopez VM, et al. A scale for the identification of the complex chronic pediatric patient (PedCom Scale): A pilot study. Anales de pediatria. 2022;97(3):155-60.

Online Resource 4D: Reference list Life Limiting Conditions (LLC)

**Definitions LLC (n=32)**	
Definition Fraser et al. (2012)	(333-348)
Definition Together for Short Lives (2018)	(349-364)
**Studies using other term patient category with definition Together for Short Lives**	
Non-malignant LCC	(349, 350)

333. Bowers AP, Chan RJ, Herbert A, Yates P. Estimating the prevalence of life-limiting conditions in Queensland for children and young people aged 0-21 years using health administration data. Australian health review. 2020;44(4):630-6.
334. Clarke T, Connolly M. Parent's Lived Experience of Memory Making With Their Child at or Near End of Life. The American journal of hospice & palliative care. 2022;39(7):798-805.
335. Constantinou G, Garcia R, Cook E, Randhawa G. Children's unmet palliative care needs: a scoping review of parents' perspectives. BMJ supportive & palliative care. 2019;9(4):439-50.
336. Coombes L, Braybrook D, Roach A, Scott H, Harardottir D, Bristowe K, et al. Achieving child-centred care for children and young people with life-limiting and life-threatening conditions-a qualitative interview study. European journal of pediatrics. 2022;181(10):3739-52.
337. Engler J, Gruber D, Engler F, Hach M, Seipp H, Kuss K, et al. Parents' Perspectives on Hospital Care for Children and Adolescents with Life-Limiting Conditions: A Grounded Theory Analysis of Narrative Interviews. Journal of palliative medicine. 2020;23(4):466-74.
338. Fahner J, Rietjens J, van der Heide A, Milota M, van Delden J, Kars M. Evaluation showed that stakeholders valued the support provided by the Implementing Pediatric Advance Care Planning Toolkit. Acta paediatrica (Oslo, Norway). 2020:237-46.
339. Fisher V, Atkin K, Fraser LK. The health of mothers of children with a life-limiting condition: A qualitative interview study. Palliative medicine. 2022;36(9):1418-25.
340. Fisher V, Fraser L, Taylor J. Experiences of fathers of children with a life-limiting condition: a systematic review and qualitative synthesis. BMJ supportive & palliative care. 2023;13(1):15-26.
341. Fraser LK, Gibson-Smith D, Jarvis S, Norman P, Parslow RC. Estimating the current and future prevalence of life-limiting conditions in children in England. Palliative medicine. 2021;35(9):1641-51.
342. Fraser LK, Gibson-Smith D, Jarvis S, Papworth A, Neefjes V, Hills M, et al. Polypharmacy in Children and Young People With Life-limiting Conditions From 2000 to 2015: A Repeated Cross-sectional Study in England. Journal of pain and symptom management. 2022;64(3):213-21.e1.
343. Hartley J, Bluebond-Langner M, Candy B, Downie J, Henderson EM. The Physical Health of Caregivers of Children With Life-Limiting Conditions: A Systematic Review. Pediatrics. 2021;148(2):1-25.
344. Hayden J, Larkin MA, Noonan H, Conroy M, Twomey F, O'Reilly V, et al. Palliative care services for children with life-limiting conditions. Ir J Med Sci. 2023;192(3):1285-1290.

345. Kalm B, Lai K, Darro N. Care of children with home mechanical ventilation in the healthcare continuum. Hospital practice. 2021;49:456-66.
346. Mulligan S, Guerin S, McKiernan A, Brown A, Hartnett M, Gray D, et al. The core features and outcomes of a specialised camp programme for children with life-limiting conditions and their families: A qualitative multi-perspective approach. Journal of child health care. 2020;24(4):515-28.
347. Sidgwick P, Fraser J, Fortune P-M, McCulloch R. Parallel planning and the paediatric critical care patient. Archives of disease in childhood. 2019;104(10):994-7.
348. Jaaniste T, Cuganesan A, Wei Ling C, Tan SC, Coombs S, Heaton M, et al. Living with a child who has a life-limiting condition: The functioning of well-siblings and parents. Child: Care, Health & Development. 2022;48(2):269-76.
349. Kiernan G, Hurley F, Price J. 'With every fibre of their being': Perspectives of healthcare professionals caring for children with non-malignant life-limiting conditions. Child: care, health and development. 2022;48(2):250-8.
350. Hurley F, Kiernan G, Price J. 'Starting Out in Haziness': Parental Experiences Surrounding the Diagnosis of their Child's Non-Malignant Life-Limiting Condition in Ireland. Journal of pediatric nursing. 2021;59:25-31.
351. Chocarro Gonzalez L, Rigal Andres M, de la Torre-Montero JC, Barcelo Escario M, Martino Alba R. Effectiveness of a Family-Caregiver Training Program in Home-Based Pediatric Palliative Care. Children (Basel, Switzerland). 2021;8(3):1-14.
352. Connor EO, Corcoran Y. Caring for a child with a life limiting condition: The experiences of nurses in an intellectual disability service provider. J Intellect Disabil. 2022;26(4):938-953.
353. Dreier LA, Zernikow B, Stening K, Wager J. Insights into the Frequency and Distinguishing Features of Sleep Disorders in Pediatric Palliative Care Incorporating a Systematic Sleep Protocol. Children (Basel, Switzerland). 2021;8(1):1-11.
354. Engler J, Schutze D, Hach M, Ploeger C, Engler F, Erler A. Specialized outpatient palliative care for children, adolescents, and their families-the special needs of the target group. Results of the ELSAH study. Bundesgesundheitsblatt Gesundheitsforschung Gesundheitsschutz. 2022;65(3):357-366.
355. Fraser LK, Murtagh FE, Aldridge J, Sheldon T, Gilbody S, Hewitt C. Health of mothers of children with a life-limiting condition: a comparative cohort study. Archives of disease in childhood. 2021;106(10):987-93.
356. Olafson EA. Finding a unique path: Embodying parenting in the midst of conflicting complexity within pediatric palliative care. Dissertation Abstracts International: Section B: The Sciences and Engineering. 2021;82(6):not specified.
357. Postavaru G-I. A meta-ethnography of parents' experiences of their children's life-limiting conditions. Qualitative Research in Psychology. 2019;16(2):253-75.
358. Barker MM, Beresford B, Bland M, Fraser LK. Prevalence and Incidence of Anxiety and Depression Among Children, Adolescents, and Young Adults With Life-Limiting Conditions: A Systematic Review and Meta-analysis. JAMA Pediatrics. 2019;173(9):835-44.
359. Barker MM, Beresford B, Fraser LK. Incidence of anxiety and depression in children and young people with life-limiting conditions. Pediatr Res. 2023;93(7):2081-2090.

360. Devitt A, Hara MO. Perceptions of nurses caring for children with life-limiting conditions in an acute setting in the Republic of Ireland. International journal of palliative nursing. 2021;27(6):281-91.
361. Dunbar H, Carter B. Experiencing place identity and place belongingness at a children's hospice: Parents' perspectives. J Child Health Care. 2021;25(1):161-171.
362. Mitterer S, Zimmermann K, Bergstrasser E, Simon M, Gerber A-K, Fink G. Measuring Financial Burden in Families of Children Living With Life-Limiting Conditions: A Scoping Review of Cost Indicators and Outcome Measures. Value in health. 2021;24(9):1377-89.
363. Oakley S, Dunbar H, de Vries K. Parent-led strategies supporting personal well-being when caring for a child with a life-limiting condition: A scoping review. Journal of child health care. 2022;26(4):648-67.
364. Postavaru G-I, Swaby H, Swaby R. A meta-ethnographic study of fathers' experiences of caring for a child with a life-limiting illness. Palliative medicine. 2021;35(2):261-79.

Online Resource 4E: Reference list Profound and Multiple Intellectual Disabilities (PMID)

**Definition PMID (n=24)**	
Definition Nakken & Vlaskamp (2002)	(365-383)
**Studies using other term patient category with definitions PMID (n=5)**	
Severe or profound intellectual and multiple disabilities	(384)
Children with significant cognitive and motor developmental delays (SDD)	(385)
Children with severe or profound intellectual disabilities	(386)
(SPID)	(387)
Children with polyhandicap	(388)
Complex and/or profound needs	

365. Peltomaki S, Pirttimaa R, Pyhalto K, Kontu EK. Setting individual goals for pupils with profound intellectual and multiple disabilities-Engaging in the activity area-based curriculum making. Education Sciences. 2021;11(9):1-16.

366. Sato T. Creation of Care Through Communication by Nurses, Welfare Workers, and Persons (Children) With Profound Intellectual Multiple Disabilities at a Day Care Center: Emancipation From the Japanese "Shame Culture". ANS Advances in nursing science. 2022;45(2):E69-E93.

367. Degache F, Bonjour A, Michaud D, Mondada L, Newman CJ. The effects of tandem skiing on posture and heart rate in children with profound intellectual and multiple disabilities. Developmental neurorehabilitation. 2019;22(4):234-9.

368. Dhondt A, Van Keer I, van der Putten A, Maes B. Communicative abilities in young children with a significant cognitive and motor developmental delay. Journal of applied research in intellectual disabilities. 2020;33(3):529-41.

369. Flink AR, Broberg M, Strid K, Thunberg G, Johnels JA. Following children with severe or profound intellectual and multiple disabilities and their mothers through a communication intervention: single-case mixed-methods findings Int J Dev Disabil. 2022;69(6):869-887.

370. Flink AR, Johnels JA, Broberg M, Thunberg G. Examining perceptions of a communication course for parents of children with profound intellectual and multiple disabilities. International Journal of Developmental Disabilities. 2022;68(2):156-67.

371. Geuze L, Goossensen A. Exploring the Experiences of Dutch Parents Caring for Children with Profound Intellectual and Multiple Disabilities: A Thematic Analysis of Their Blogs. Global qualitative nursing research. 2021;8:23333936211028170.

372. Geuze L, Goossensen A. Caring for children with profound intellectual and multiple disabilities: Images and metaphors expressed by Dutch parents. Disability & Society. 2023:Not specified.

373. Geuze L, Goossensen A, Schrevel S. "continuously struggling for balance": The lived experiences of dutch parents caring for children with profound intellectual and multiple disabilities. J Intellect Dev Disabil. 2023 Jun;48(2):161-171.

374. Kruithof K, Olsman E, Nieuwenhuijse A, Willems D. Parents' views on medical decisions related to life and death for their ageing child with profound intellectual and multiple disabilities: A qualitative study. Research in developmental disabilities. 2022;121:104154.

375. Kruithof K, Willems D, Etten-Jamaludin F, Olsman E. Parents' knowledge of their child with profound intellectual and multiple disabilities: An interpretative synthesis. Journal of Applied Research in Intellectual Disabilities. 2020;33(6):1141-50.
376. Lahaije STA, Luijkx J, Waninge A, van der Putten AAJ. Support needs of families with a child with profound intellectual and multiple disabilities. Int J Dev Disabil. 2023;70(7):1184-1197.
377. Luijkx J, van der Putten AAJ, Vlaskamp C. A valuable burden? The impact of children with profound intellectual and multiple disabilities on family life. Int J Dev Disabil. 2019;44(2):184-9.
378. Neyroud MC, Newman CJ. Parents' Perspectives on Adaptive Sports in Children with Profound Intellectual and Multiple Disabilities. Children (Basel, Switzerland). 2021;8(9):1-10.
379. Nieuwenhuijse AM, Willems DL, Olsman E. Physicians' perceptions on Quality of Life of persons with profound intellectual and multiple disabilities: A qualitative study. Journal of Intellectual and Developmental Disability. 2020;45(2):176-83.
380. Petitpierre G, Dind J, De Blasio C, Gremaud G. Odour detection in children and young people with profound intellectual and multiple disabilities. J Appl Res Intellect Disabil. 2022;35(2):519-530.
381. Rensfeldt Flink A, Bostrom P, Gillberg C, Lichtenstein P, Lundstrom S, Asberg Johnels J. Exploring co-occurrence of sensory, motor and neurodevelopmental problems and epilepsy in children with severe-profound intellectual disability. Research in developmental disabilities. 2021;119:104114.
382. Skarsaune SN, Hanisch HM. Holding and Professional Care: On Self-Determination for Persons With Profound Intellectual and Multiple Disabilities. Research & Practice for Persons with Severe Disabilities. 2023;48(1):25-40.
383. Van Keer I, Dhondt A, Van der Putten A, Maes B. Lessons learned: A critical reflection on child- and contextual variables related to the development of children with a significant cognitive and motor developmental delay. Research in developmental disabilities. 2022;120:104142.
384. Rensfeld Flink A, Thunberg G, Nyman A, Broberg M, Asberg Johnels J. Augmentative and alternative communication with children with severe/profound intellectual and multiple disabilities: speech language pathologists' clinical practices and reasoning. Disability and rehabilitation Assistive technology. 2022:1-13.
385. Dhondt A, Van keer I, van der Putten A, Maes B. Analysis of early expressive communicative behaviour of young children with significant cognitive and motor developmental delays. British Journal of Learning Disabilities. 2022:Not specified.
386. Vandesande S, Steegmans D, Maes B. Parents’ views on facilitating and inhibiting factors in the development of attachment relationships with their children with severe disabilities. Journal of Social & Personal Relationships. 2023;40(2):600-23.
387. Rousseau MC, Guilluy E, Leblanc J, Willocq D, Demoures AC, Carteron L, et al. Impact of an intensive multimodal educative program on behavioral disorders of poly handicapped patients: a randomized controlled trial. Archives de pediatrie. 2022;29(4):292-9.
388. Fraser L, Johnston S, Wardrop P, Shanks M, Allen A. Long-term quality of life in children with complex needs undergoing cochlear implantation. International journal of pediatric otorhinolaryngology. 2020;136:110223.

Online Resource 4F: Reference list Pediatric Chronic Conditions (PCC)

**Definitions PCC (n=21)**	
Definition Mokkink et al. (2008)	(389-409)
**Additional information**	
Sociodemographic aspects of illness 397	(397)
Daily life limitations	(398-400, 404, 406)
Duration of disease	(397)
Psychological problems	(409)

389. Barnard-Brak L, McGaha V, Little TD, Fearon-Drake D. Medical Home Outcomes for School Aged Children With Chronic Health Care Needs: A Mokken Analysis. Quality management in health care. 2023;32(1):16-21.
390. Pinquart M. Posttraumatic stress symptoms and disorders in parents of children and adolescents with chronic physical illnesses: A meta-analysis. Journal of Traumatic Stress. 2019;32(1):88-96.
391. Pinquart M. Featured Article: Depressive Symptoms in Parents of Children With Chronic Health Conditions: A Meta-Analysis. Journal of pediatric psychology. 2019;44(2):139-49.
392. Pinquart M. Posttraumatic stress symptoms and disorders in children and adolescents with chronic physical illnesses: A meta-analysis. Journal of Child & Adolescent Trauma. 2020;13(1):1-10.
393. Pinquart M. Health-related quality of life of young people with and without chronic conditions. Journal of Pediatric Psychology. 2020;45(7):780-92.
394. Wilkin T, Stott A, Lin JL, Pate J, McEwen A, Verhagen A, et al. Free Online Decision Tools to Support Parents Making Decisions About Their Children's Chronic Health Condition: An Environmental Scan. Acad Pediatr. 2023;23(5):874-883.
395. Zylbersztejn A, Verfurden M, Hardelid P, Gilbert R, Wijlaars L. Phenotyping congenital anomalies in administrative hospital records. Paediatric and perinatal epidemiology. 2020;34(1):21-8.
396. Davis E, Smith-Adcock S, Towns L. Experiences of elementary school counselors and students in using reality art therapy to address chronic conditions. Professional School Counseling. 2019;22(1).
397. Barrio Cortes J, Suarez Fernandez C, Bandeira de Oliveira M, Munoz Lagos C, Beca Martinez MT, Lozano Hernandez C, et al. Chronic diseases in the paediatric population: Comorbidities and use of primary care services. Anales de Pediatria. 2020;93(3):183-93.
398. Brigden A, Anderson E, Linney C, Morris R, Parslow R, Serafimova T, et al. Digital Behavior Change Interventions for Younger Children With Chronic Health Conditions: Systematic Review. Journal of medical Internet research. 2020;22(7):e16924.
399. Deavin A. A qualitative exploration of family members' experiences of paediatric chronic illness. Dissertation Abstracts International: Section B: The Sciences and Engineering. 2021;82(6):Not specified.
400. Long E, Barrett T, Lockhart G. Chronic health conditions and adolescent friendship: Perspectives from social network analysis. International Journal of Adolescent Medicine and Health. 2021;33(5):20180293.

401. Martinez B, Pechlivanoglou P, Meng D, Traubici B, Mahood Q, Korczak D, et al. Clinical Health Outcomes of Siblings of Children with Chronic Conditions: A Systematic Review and Meta-Analysis. The Journal of pediatrics. 2022;250:83-92.e8.
402. Maurice-Stam H, Nijhof SL, Monninkhof AS, Heymans HSA, Grootenhuis MA. Review about the impact of growing up with a chronic disease showed delays achieving psychosocial milestones. Acta Paediatrica. 2019;108(12):2157-69.
403. Rollins J, Rollins C, Boocks LA, Sitz T. Supporting children living with chronic medical conditions through empathetic art. Journal of Child and Family Studies. 2020;29(8):2218-33.
404. Schlebusch L, Huus K, Samuels A, Granlund M, Dada S. Participation of young people with disabilities and/or chronic conditions in low- and middle-income countries: a scoping review. Developmental medicine and child neurology. 2020;62(11):1259-65.
405. N, Pereira M, Otto C, Ravens-Sieberer U, Canavarro MC, Bullinger M. Do 8- to 18-year-old children/adolescents with chronic physical health conditions have worse health-related quality of life than their healthy peers? a meta-analysis of studies using the KIDSCREEN questionnaires. Quality of life research. 2019;28(7):1725-50.
406. Wolock ER, Queen AH, Rodríguez GM, Weisz JR. Chronic Illness and Internalizing Symptomatology in a Transdiagnostic Clinical Sample of Youth. J Pediatr Psychol. 2020;45(6):633-42.
407. Wong Chung R, Willemen A, Voorman J, Ketelaar M, Becher J, Verheijden J, et al. Managing oneself or managing together? Parents’ perspectives on chronic condition self-management in Dutch pediatric rehabilitation services. Disability and Rehabilitation. 2019;42:1-11.
408. Wong Chung R, Willemen A, Voorman J, Ketelaar M, Becher J, Verheijden J, et al. Managing oneself or managing together? Parents' perspectives on chronic condition self-management in Dutch pediatric rehabilitation services. Disability and Rehabilitation: An International, Multidisciplinary Journal. 2020;42(23):3348-58.
409. Dinleyici M, Carman KB, Ozdemir C, Harmanci K, Eren M, Kirel B, et al. Quality-of-life Evaluation of Healthy Siblings of Children with Chronic Illness. Balkan medical journal. 2019;37(1):34-42.

Online Resource 4G: Reference list Technology Dependence (TD)

**Definition TD (n=10)**	
Definition US Congress Office of Technology Assessment (OTA)	(410-416)
**Studies using other term patient category with definitions TD (n=3)**	
Medically complex, technology-dependent children	(417)
Children dependent on respiratory support	(418)
Children with invasive long-term ventilator dependence	(419)

410. Fernandez-Medina IM, Granero-Molina J, Hernandez-Padilla JM, Jimenez-Lasserrotte MDM, Ruiz-Fernandez MD, Fernandez-Sola C. Socio-family support for parents of technology-dependent extremely preterm infants after hospital discharge. Journal of child health care : for professionals working with children in the hospital and community. 2022;26(1):42-55.
411. Sobotka SA, Lynch E, Quinn MT, Awadalla SS, Agrawal RK, Peek ME. Unmet Respite Needs of Children With Medical Technology Dependence. Clinical pediatrics. 2019;58(11):1175-86.
412. Toly VB, Blanchette JE, Al-Shammari T, Musil CM. Caring for technology-dependent children at home: Problems and solutions identified by mothers. Appl Nurs Res. 2019;50:151195.
413. Camara C, Callum J. Care of children and young people who are dependent on technology. British Journal of Nursing. 2020;29(7):403-5.
414. Chan YH, Lim CZ-R, Bautista D, Malhotra R, Ostbye T. The Health and Well-Being of Caregivers of Technologically Dependent Children. Global pediatric health. 2019;6:2333794X18823000.
415. Sobotka SA, Lynch E, Peek ME, Graham RJ. Readmission drivers for children with medical complexity: Home nursing shortages cause health crises. Pediatric pulmonology. 2020;55(6):1474-80.
416. Toly VB, Zauszniewski JA, Yu J, Sattar A, Rusincovitch B, Musil CM. Resourcefulness Intervention Efficacy for Parent Caregivers of Technology-Dependent Children: A Randomized Trial. Western journal of nursing research. 2022;44(3):296-306.
417. Mitchell TK, Bray L, Blake L, Dickinson A, Carter B. 'It doesn't feel like our house anymore': The impact of medical technology upon life at home for families with a medically complex, technology-dependent child. Health & place. 2022;74:102768.
418. Dumas HM, Hughes ML, O'Brien JE. Children Dependent on Respiratory Support: A 10-Year Review from One Pediatric Post-Acute Care Hospital. Pediatric pulmonology. 2020:2050-4.
419. Giambra BK, Mangeot C, Benscoter DT, Britto MT. A Description of Children Dependent on Long Term Ventilation via Tracheostomy and Their Hospital Resource Use. Journal of pediatric nursing. 2021;61:96-101.

Online Resource 4H: Reference list Pediatric Chronic Critical Illness (PCCI)

**Definitions PCCI (n=9)**	
Shapiro et al. (2017)	(420-426)
Definition Shapiro with additional information	(427)
Demirkiran et al. (2021)	(428)

420. Grandjean C, Ullmann P, Marston M, Maitre M-C, Perez M-H, Ramelet A-S. Sources of Stress, Family Functioning, and Needs of Families With a Chronic Critically Ill Child: A Qualitative Study. Frontiers in pediatrics. 2021;9:740598.
421. Ruth AR, Boss RD, Donohue PK, Shapiro MC, Raisanen JC, Henderson CM. Living in the Hospital: The Vulnerability of Children with Chronic Critical Illness. The Journal of clinical ethics. 2020;31(4):340-52.
422. Shappley RKH, Noles DL, Spentzas T. Pediatric Chronic Critical Illness: Validation, Prevalence, and Impact in a Children's Hospital. Pediatric critical care medicine : a journal of the Society of Critical Care Medicine and the World Federation of Pediatric Intensive and Critical Care Societies. 2021;22(12):e636-e9.
423. Troch R, Schwartz J, Boss R. Slow and Steady: A Systematic Review of ICU Care Models Relevant to Pediatric Chronic Critical Illness. Journal of pediatric intensive care. 2020;9(4):233-40.
424. Wright-Sexton LA, Compretta CE, Blackshear C, Henderson CM. Isolation in Parents and Providers of Children With Chronic Critical Illness. Pediatric critical care medicine : a journal of the Society of Critical Care Medicine and the World Federation of Pediatric Intensive and Critical Care Societies. 2020;21(8):e530-e7.
425. Zorko DJ, McNally JD, Rochwerg B, Pinto N, O'Hearn K, Almazyad MA, et al. Defining Pediatric Chronic Critical Illness: A Scoping Review. Pediatric Critical Care Medicine. 2023;24(2):91-103.
426. Shapiro MC, Boss RD, Donohue PK, Weiss EM, Madrigal V, Henderson CM. A Snapshot of Chronic Critical Illness in Pediatric Intensive Care Units. Journal of Pediatric Intensive Care. 2021;13(1):55-62.
427. Barone S, Boss RD, Raisanen JC, Shepard J, Donohue PK. Our life at home: Photos from families inform discharge planning for medically complex children. Birth (Berkeley, Calif). 2020;47(3):278-89.
428. Demirkiran H, Kilic M, Tomak Y, Dalkiran T, Yurttutan S, Basaranoglu M, et al. Evaluation of the incidence, characteristics, and outcomes of pediatric chronic critical illness. PloS one. 2021;16(5):e0248883.

Online Resource 4I: Reference list Other Findings

**Other findings (n=94)**	(429-522)
**Other patient categories (n=18)**	
Children with physical disabilities	(429)
Children with disabilities	(430, 431)
Long stay patients	(432)
Medically fragile infants	(433, 434)
Genetic condition in children	(435)
Children with complex health conditions	(436)
Children with complex health needs	(437)
Children with complex medical needs	(438)
Children with complex chronic health conditions	(439)
Children with chronic illness and medical complexity	(440)
Children with multiple complex needs	(441)
Children with health complexities and special needs	(442)
Children with complex care needs	(443)
Children with palliative care needs	(444, 445)
Children with severe multiple disabilities	(446)
**PCC definitions with considerable variation (n=21)**	
Time indication	(447-464)
Medical care	(449-453, 458, 460-462, 465)
Daily life limitations	(449, 453, 460, 461, 464-466)
Quality of life	(447, 464)
Psychological problems	(458, 463, 467)
**Definition used for other patient category (n=36)**	
Population CMC with definition CCC	(468-479)
Population CMC with definition CYSHCN	(480-489)
Population CYSHCN with definition CMC	(490)
Population CCC with definition CMC	(491-493)
CYSHCN as a **s**ubset of CMC	(494-497)
Population TD with definition CMC	(498-500)
Population TD with definition Feudtner et al.(2005	(501-503)
**Operationalization tools used as definition (n=19)**	
CSHCN screener	(504, 505)
PMCA	(506-508)
CSHCN according to the SHCN screener Bethell et al.	(509-520)
Chronic conditions indicator	(521)
NSCH	(522)

429. Ow N, Appau A, Matout M, Mayo NE. What is QOL in children and adolescents with physical disabilities? A thematic synthesis of pediatric QOL literature. Quality of Life Research: An International Journal of Quality of Life Aspects of Treatment, Care & Rehabilitation. 2021;30(5):1233-48.

430. Chien AT, Toomey SL, Kuo DZ, Van Cleave J, Houtrow AJ, Okumura MJ, et al. Care Quality and Spending Among Commercially Insured Children With Disabilities. Academic pediatrics. 2019;19(3):291-9.
431. Heneghan JA, Sobotka SA, Hallman M, Pinto N, Killien EY, Palumbo K, et al. Outcome Measures Following Critical Illness in Children With Disabilities: A Scoping Review. Frontiers in pediatrics. 2021;9:689485.
432. Edwards JD, Williams EP, Wagman EK, McHale BL, Malone CT, Kernie SG. A Single-Centered Randomized Controlled Trial of Primary Pediatric Intensivists and Nurses. Journal of intensive care medicine. 2022;37(12):1580-6.
433. MacKay L, Benzies K, Barnard C, Raffin Bouchal S. Parental Experiences Caring for Their Hospitalized Medically Fragile Infants: A Description of Grief, Stress, and Coping. The Canadian journal of nursing research. 2021;53(3):191-201.
434. Mackay LJ, Benzies KM, Barnard C, Hayden KA. A scoping review of parental experiences caring for their hospitalised medically fragile infants. Acta paediatrica (Oslo, Norway). 2020;109(2):266-75.
435. Kueny AM, Ayres L, Tripp-Reimer T. Old order Amish family management of children with genetic conditions. Journal of Family Nursing. 2021;27(4):327-38.
436. Azar R, Doucet S, Horsman AR, Charlton P, Luke A, Nagel DA, et al. A concept analysis of children with complex health conditions: implications for research and practice. BMC pediatrics. 2020;20(1):251.
437. Ming DY, Zhao C, Tang X, Chung RJ, Rogers UA, Stirling A, et al. Predictive Modeling to Identify Children With Complex Health Needs At Risk for Hospitalization. Hospital pediatrics. 2023.
438. DeFoor W, Nehus E, Schulte M, Huesman S, Libs A, Niehaus R, et al. Enteral nutrition and the risk of nephrolithiasis in complex pediatric patients. Journal of pediatric urology. 2022;18(6):743.e1-6.
439. Fernandez HGC, Moreira MCN, Gomes R. Making decisions on health care for children / adolescents with complex chronic conditions: a review of the literature. Cien Saude Colet. 2019;24(6):2279-92.
440. Bravo L, Killela MK, Reyes BL, Santos KMB, Torres V, Huang C-C, et al. Self-Management, Self-Efficacy, and Health-Related Quality of Life in Children With Chronic Illness and Medical Complexity. Journal of Pediatric Healthcare. 2020;34(4):304-14.
441. Van Dongen T, Sabbe B, Glazemakers I. Collaboration for children with complex needs: What adolescents, parents, and practitioners tell us. Journal of child health care. 2020;24(1):19-32.
442. Perez-Ardanaz B, Morales-Asencio JM, Garcia-Pinero JM, Lupianez-Perez I, Morales-Gil IM, Kaknani-Uttumchandani S. Socioeconomic Status and Health Services Utilization for Children With Complex Chronic Conditions Liable to Receive Nurse-Led Services: A Cross-Sectional Study. Journal of nursing scholarship. 2019;51(5):518-25.
443. Luke A, Luck KE, Doucet S. Experiences of Caregivers as Clients of a Patient Navigation Program for Children and Youth with Complex Care Needs: A Qualitative Descriptive Study. International journal of integrated care. 2020;20(4):10.

444. Clancy M, Taylor J, Bradbury-Jones C, Phillimore J. A systematic review exploring palliative care for families who are forced migrants. Journal of advanced nursing. 2020;76(11):2872-84.
445. Breneol S, Doucet S, McIsaac J-L, Riveroll A, Cassidy C, Charlton P, et al. Programmes to support transitions in community care for children with complex care needs: a scoping review. BMJ open. 2022;12(7):e056799.
446. Mensch SM, Echteld MA, Lemmens R, Oppewal A, Evenhuis HM, Rameckers EAA. The relationship between motor abilities and quality of life in children with severe multiple disabilities. Journal of intellectual disability research. 2019;63(2):100-12.
447. Cobham VE, Hickling A, Kimball H, Thomas HJ, Scott JG, Middeldorp CM. Systematic review: Anxiety in children and adolescents with chronic medical conditions. Journal of the American Academy of Child & Adolescent Psychiatry. 2020;59(5):595-618.
448. Luther AWM, Reaume SV, Qadeer RA, Thompson K, Ferro MA. Substance use disorders among youth with chronic physical illness. Addictive Behaviors. 2020;110:106517.
449. Ullah F, Kaelber DC. Using Large Aggregated De-Identified Electronic Health Record Data to Determine the Prevalence of Common Chronic Diseases in Pediatric Patients Who Visited Primary Care Clinics. Acad Pediatr. 2021;21(6):1084-93.
450. van Schelven F, Boeije H, Marien V, Rademakers J. Patient and Public Involvement of young people with a chronic condition in projects in health and social care: A scoping review. Health Expectations: An International Journal of Public Participation in Health Care & Health Policy. 2020;23(4):789-801.
451. da Silveira A, Chaves de Vargas TG, Portela de Oliveira J, Henrich Cazuni M, da Rosa B, de Vargas Bueno T, et al. Nursing care for children and adolescents with special health needs. Ciencia, Cuidado e Saude. 2022;21:1-7.
452. Hookway L, Lewis J, Brown A. The challenges of medically complex breastfed children and their families: A systematic review. Maternal & child nutrition. 2021;17(4):e13182.
453. Nabors L, Liddle M, Graves ML, Kamphaus A, Elkins J. A family affair: Supporting children with chronic illnesses. Child: Care, Health and Development. 2019;45(2):227-33.
454. Pugh P, Hemingway P, Christian M, Higginbottom G. Children's, parents', and other stakeholders' perspectives on the factors influencing the initiation of early dietary change in the management of childhood chronic disease: A mixed studies systematic review using a narrative synthesis. Patient Education and Counseling. 2021;104(4):844-57.
455. Rennie K, Racine M, Ruda VM. Childhood chronic illnesses in the schools. Pediatric health conditions in schools: A clinician's guide for working with children, families, and educators. 2020:3-17.
456. Toledano-Toledano F, Dominguez-Guedea MT. Psychosocial factors related with caregiver burden among families of children with chronic conditions. BioPsychoSocial medicine. 2019;13:6.
457. Wright SR, Graham CD, Houghton R, Ghiglieri C, Berry E. Acceptance and commitment therapy (ACT) for caregivers of children with chronic conditions: A mixed methods systematic review (MMSR) of efficacy, process, and acceptance. Journal of Contextual Behavioral Science. 2023;27:72-97.

458. Courtwright SE, Le Pard A, Jones J. Emotional wellbeing in adolescents living with chronic conditions: A metasynthesis of the qualitative literature. Journal of Adolescent Health. 2022;70(6):864-76.
459. Sheng N, Ma J, Ding W, Zhang Y. Effects of caregiver-involved interventions on the quality of life of children and adolescents with chronic conditions and their caregivers: A systematic review and meta-analysis. Quality of Life Research: An International Journal of Quality of Life Aspects of Treatment, Care & Rehabilitation. 2019;28(1):13-33.
460. Spurr S, Danford CA, Roberts KJ, Sheppard-LeMoine D, Machado Silva-Rodrigues F, Darezzo Rodrigues Nunes M, et al. Fathers' Experiences of Caring for a Child with a Chronic Illness: A Systematic Review. Children (Basel, Switzerland). 2023;10(2):197.
461. Helton JJ, House NG. Children with chronic health conditions and maltreatment re-report. Children and Youth Services Review. 2019;105.
462. Ruskin D, Young M, Sugar C, Nofech-Mozes J. Mindfulness and acceptance interventions for parents of children and adolescents diagnosed with chronic medical conditions: A systematic review. The Journal of Alternative and Complementary Medicine. 2021;27(2):120-35.
463. Stefko JM, Stolfi A, Pascoe JM. Screening for Children's Chronic Health Conditions and Their Strengths and Difficulties in Primary Care. Journal of developmental and behavioral pediatrics. 2022;43(1):e1-e8.
464. Thomas S, White V, Ryan N, Byrne L. Effectiveness of play therapy in enhancing psychosocial outcomes in children with chronic illness: A systematic review. Journal of pediatric nursing. 2022;63:e72-e81.
465. Takken T, de Jong N, Duijf M, van den Berg S, Wendel-Vos W. Results from the Netherlands' 2018 Report Card and Report Card+ on physical activity for children and youth with and without chronic medical condition. Public health. 2020;185:161-6.
466. Spencer NJ, Ludvigsson J, You Y, Francis K, Abu Awad Y, Markham W, et al. Household income and maternal education in early childhood and activity-limiting chronic health conditions in late childhood: findings from birth cohort studies from six countries. Journal of epidemiology and community health. 2022:939-48.
467. Noonan K, Reichman NE, Corman H, Jimenez ME. School and Community Involvement of Adolescents With Chronic Health Conditions. The Journal of adolescent health. 2020;67(4):576-82.
468. Ahuja N, Mack WJ, Russell CJ. Technology-Dependent Pediatric Inpatients at Children's Versus Non children's Hospitals. Hospital pediatrics. 2020;10(6):481-8.
469. Braun L, Steurer M, Henry D. Healthcare Utilization of Complex Chronically Ill Children Managed by a Telehealth-Based Team. Frontiers in pediatrics. 2021;9:689572.
470. Heneghan JA, Goodman DM, Ramgopal S. Variable Identification of Children With Medical Complexity in United States PICUs. Pediatric critical care medicine. 2023;24(1):56-61.
471. Howell KD, Kelly MM, DeMuri GP, McBride JA, Katz B, Edmonson MB, et al. COVID-19 Vaccination Intentions for Children With Medical Complexity. Hospital pediatrics. 2022;12(9):e295-e302.

472. Larson IA, Rodean J, Richardson T, Bergman D, Morehous J, Colvin JD. Agreement of Provider and Parent Perceptions of Complex Care Medical Homes After a Care Management Intervention. Journal of pediatric health care. 2021;35(1):91-8.
473. Markham JL, Hall M, Goldman JL, Bettenhausen JL, Gay JC, Feinstein J, et al. Readmissions Following Hospitalization for Infection in Children With or Without Medical Complexity. Journal of hospital medicine. 2021;16(3):134-41.
474. Nkoy F, Stone B, Hofmann M, Fassl B, Zhu A, Mahtta N, et al. Home-Monitoring Application for Children With Medical Complexity: A Feasibility Trial. Hospital pediatrics. 2021;11(5):492-502.
475. Notario PM, Gentile E, Amidon M, Angst D, Lefaiver C, Webster K. Home-Based Telemedicine for Children with Medical Complexity. Telemedicine journal and e-health. 2019;25(11):1123-32.
476. Rush M, Herrera N, Melwani A. Discharge Communication Practices for Children With Medical Complexity: A Retrospective Chart Review. Hospital pediatrics. 2020;10(8):651-6.
477. Danzig JA, Katz EB. Musculoskeletal and skin considerations in children with medical complexity: Common themes and approaches to management. Current problems in pediatric and adolescent health care. 2021;51(9):101074.
478. Leary JC, Price LL, Scott CER, Kent D, Wong JB, Freund KM. Developing Prediction Models for 30-Day Unplanned Readmission Among Children With Medical Complexity. Hospital pediatrics. 2019;9(3):201-8.
479. Auger KA, Shah SS, Huang B, Brady PW, Weinberg SH, Reamer E, et al. Discharge Medical Complexity, Change in Medical Complexity and Pediatric 30-day Readmission. Journal of hospital medicine. 2019;14(8):474-81.
480. Hogan AK, Galligan MM, Stack NJ, Leach KF, Aredas BL, English R, et al. A Tertiary Care-based Complex Care Program: Improving Care for Children With Medical Complexity. Medical care. 2020;58(11):958-62.
481. Oliveira PV, Enes CC, Nucci LB. How are children with medical complexity being identified in epidemiological studies? A systematic review. World J Pediatr. 2023;19(10):928-938.
482. Schulz J, Serrano V, Buchholz M, Natvig C, Talmi A. Increased behavioral health needs and continued psychosocial stress among children with medical complexity and their families during the covid-19 pandemic. Infant Ment Health J. 2022;43(1):111-126.
483. Yoder A, Ogden R, Jr., Williams K. Pharmacist Integration Into Care for Patients With Medical Complexity: An Intervention to Optimize Complex Therapeutic Regimens Through Medication Services. Hospital pediatrics. 2021;11(3):303-7.
484. Yamoah J. The role of social support in mitigating parental burnout for mothers of children with medical complexity. Dissertation Abstracts International: Section B: The Sciences and Engineering. 2021;82(10):Not specified.
485. Baddour K, Mady LJ, Schwarzbach HL, Sabik LM, Thomas TH, McCoy JL, et al. Exploring caregiver burden and financial toxicity in caregivers of tracheostomy-dependent children. International journal of pediatric otorhinolaryngology. 2021;145:110713.

486. Suzuki S, Kamibeppu K. Impact of respite care on health-related quality of life in children with medical complexity: A parent proxy evaluation. Journal of pediatric nursing. 2022;67:e215-e23.
487. Ware EJ, Beveridge MS, Rosado AI, Nageswaran S. Practical Needs in the Home Care of Latino Children With Medical Complexity. Home healthcare now. 2020;38(4):202-8.
488. Blanco MA, Lilly CM, Bavinger BC, Garcia S, Hojnicki MP. Caring for Medically Complex Children in the Outpatient Setting. Advances in pediatrics. 2021;68:89-102.
489. Hounsell KG, Moore C, Zahavi A, Arje D, Weiser N, Esser K, et al. The Experience of Housing Needs Among Families Caring for Children With Medical Complexity. Pediatrics. 2021;148(1):1-9.
490. Tennant R, Allana S, Mercer K, Burns CM. Exploring the Experiences of Family Caregivers of Children With Special Health Care Needs to Inform the Design of Digital Health Systems: Formative Qualitative Study. JMIR formative research. 2022;6(1):e28895.
491. Dunbar PJ, Sobotka SA, Rodean J, Pulcini CD, Macy ML, Thomson J, et al. Prevalence of and Spending on Ear, Nose, Throat, and Respiratory Infections Among Children With Chronic Complex Conditions. Academic pediatrics. 2023;23(2):434-40.
492. Mattiello RMA, Pazin-Filho A, Aragon DC, Cupo P, Carlotti APCP. Impact of children with complex chronic conditions on costs in a tertiary referral hospital. Revista de saude publica. 2022;56:89.
493. Novais MCM, Victor DS, da Silva Rodrigues D, Freitas BO, Barreto NMPV, de Jesus da Silva Mendes D, et al. Factors associated with de-hospitalization of children and adolescents with complex chronic condition. Revista Paulista de Pediatria. 2021;39:2020118.
494. Lynch A, Raziano V, Feehan K, Thompson E, Massey P, Turchi R. Unmet Needs of Male Caregivers of Children and Youth with Special Health Care Needs. Maternal and child health journal. 2021;25(12):1992-2001.
495. Swann-Thomsen HE, Vineyard J, Hanks J, Hofacer R, Sitts C, Flint H, et al. Pediatric care coordination and risk tiering: Moving beyond claims data. Journal of pediatric rehabilitation medicine. 2021;14(3):485-93.
496. Yu JA, Bayer ND, Beach SR, Kuo DZ, Houtrow AJ. A National Profile of Families and Caregivers of Children With Disabilities and/or Medical Complexity. Acad Pediatr. 2022;22(8):1489-98.
497. Yamoah J, Brown L. Understanding the types of social support that can mitigate parental burnout in mothers of children with medical complexity. Child Care Health Dev. 2023 Jul;49(4):732-739.
498. Choi YH, Kim MS, Kim CH, Song IG, Park JD, In Suh D, et al. Looking into the life of technology-dependent children and their caregivers in Korea: lifting the burden of too many responsibilities. BMC pediatrics. 2020;20(1):486.
499. Matsuzawa A, Arai J, Shiroki Y, Hirasawa A. Healthcare for children depend on medical technology and parental quality of life in Japan. Pediatrics international. 2022;64(1):e15006.
500. Rush M, Khan A, Barber J, Bloom M, Anspacher M, Fratantoni K, et al. Length of Stay and Barriers to Discharge for Technology-Dependent Children During the COVID-19 Pandemic. Hospital pediatrics. 2023;13(1):80-7.

501. Heneghan JA, Reeder RW, Dean JM, Meert KL, Berg RA, Carcillo J, et al. Characteristics and Outcomes of Critical Illness in Children With Feeding and Respiratory Technology Dependence. Pediatric critical care medicine. 2019;20(5):417-25.
502. Moynihan K, Franca UL, Casavant DW, Graham RJ, McManus ML. Hospital Access Patterns of Children With Technology Dependence. Pediatrics. 2023;151(4).
503. Norton M, Hagstrom A. Finding a New Normal: Maternal Experiences Transitioning to Home From the Neonatal Intensive Care Unit Caring for Technology-Dependent Infants. Advances in neonatal care. 2022;22(1):E2-E12.
504. Hofacer RD, Panatopoulos A, Vineyard J, Tivis R, Nguyen E, Jingjing N, et al. Clinical Care Coordination in Medically Complex Pediatric Cases: Results From the National Survey of Children With Special Health Care Needs. Global pediatric health. 2019;6:2333794X19847911.
505. Ross MH, Parnell LS, Spears TG, Ming DY. Telemedicine Video Visits for Children with Medical Complexity in a Structured Clinical Complex Care Program. Global pediatric health. 2020;7:2333794X20952196.
506. Ravid NL, Zamora K, Rehm R, Okumura M, Takayama J, Kaiser S. Implementation of a multidisciplinary discharge videoconference for children with medical complexity: a pilot study. Pilot and feasibility studies. 2020;6:27.
507. Reuland CP, Collins J, Chiang L, Stewart V, Cochran AC, Coon CW, et al. Oregon's approach to leveraging system-level data to guide a social determinants of health-informed approach to children's healthcare. BMJ Innovations. 2021;7(1):18-25.
508. Vaz LE, Wagner DV, Ramsey KL, Jenisch C, Austin JP, Jungbauer RM, et al. Identification of Caregiver-Reported Social Risk Factors in Hospitalized Children. Hosp Pediatr. 2020;10(1):20-8.
509. Akobirshoev I, Parish S, Mitra M, Dembo R. Impact of Medical Home on Health Care of Children With and Without Special Health Care Needs: Update from the 2016 National Survey of Children's Health. Maternal and child health journal. 2019;23(11):1500-7.
510. Evangelista Cabral I, Silva da Motta I, Pereira Pimentel TG, Pacheco de Oliveira Corrêa M, Moreira Arrué A, Neves ET. Demandas de crianças com necessidades especiais de saúde na atenção primária da cidade do rio de janeiro. Ciencia, Cuidado e Saude. 2020;19:1-10.
511. Geweniger A, Haddad A, Barth M, Hogl H, Mund A, Insan S, et al. Mental health of children with and without special healthcare needs and of their caregivers during COVID-19: a cross-sectional study. BMJ paediatrics open. 2022;6(1):e001509.
512. Hagerman TK, McKernan GP, Carle AC, Yu JA, Stover AD, Houtrow AJ. The Mental and Physical Health of Mothers of Children with Special Health Care Needs in the United States. Maternal and child health journal. 2022;26(3):500-10.
513. Montes G. US children with special health care needs and ethnic discrimination: results from multivariate modeling. World journal of pediatrics. 2019;15(2):182-9.
514. Rast JE, Roux AM, Connor G, Ezeh TH, Shea L, Turchi RM, et al. The Medical Home and Mental Health Services in Children and Youth with Special Health Care Needs. Maternal and child health journal. 2023 Jun;27(6):1097-1106.

515. Rennane S, Dick A. Effects of Medicaid Automatic Enrollment on Disparities in Insurance Coverage and Caregiver Burden for Children with Special Health Care Needs. Medical care research and review. 2023;80(1):65-78.
516. Vasan A, Kyle MA, Venkataramani AS, Kenyon CC, Fiks AG. Inequities in Time Spent Coordinating Care for Children and Youth with Special Health Care Needs. Acad Pediatr. 2023;23(8):1526-1534.
517. Warden C, Yun K, Semere W. Using the Children with Special Health Care Needs Screener with Immigrant Families: An Analysis of the National Survey of Children's Health. J Immigr Minor Health. 2019;21(1):189-97.
518. Kaji N, Ando S, Nishida A, Yamasaki S, Kuwabara H, Kanehara A, et al. Children with special health care needs and mothers' anxiety/depression: Findings from the Tokyo Teen Cohort study. Psychiatry and clinical neurosciences. 2021;75(12):394-400.
519. Chisolm DJ, Keedy HE, Dolce M, Chavez L, Abrams MA, Sanders L. Do health literacy disparities explain racial disparities in family-centered care for youths with special health care needs? Patient Educ Couns. 2021;104(4):887-95.
520. Kan K, Gupta R, Davis MM, Heard-Garris N, Garfield C. Adverse Experiences and Special Health Care Needs Among Children. Maternal and child health journal. 2020;24(5):552-60.
521. Feinstein JA, Rodean J, Hall M, Doupnik SK, Gay JC, Markham JL, et al. Outpatient Prescription Opioid Use in Pediatric Medicaid Enrollees With Special Health Care Needs. Pediatrics. 2019;143(6):1-9.
522. Andersen JA, Morrow JE, Gibbs L, Hernandez NI. Caregiver reports of physician risk counseling for adolescents with special health care needs. Patient Education and Counseling. 2022;105(6):1581-1586.
