# Supplementary material for: Definitions and operationalizations of pediatric chronic patients: a scoping review
Source: Eur J Pediatr. 2025 Nov 25;184(12):789. doi: 10.1007/s00431-025-06556-0 (PMC12644160; doi:10.1007/s00431-025-06556-0)
Supplement: Supplementary file 5 — Online resource 5 (PDF 141 KB) [file 431_2025_6556_MOESM5_ESM.pdf]

**Definitions and operationalizations of pediatric chronic patients: A scoping review**

**European Journal of Pediatrics**

Cor-Jan van der Perk (CJP) [a,b,c], MSc, RN; Lisa-Maria van Klaveren (LK) [c,d], MSc, MA; Karlijn S. Timmer (KT) [a,b,c], MSc, RN, Heleen N. Haspels [a,e] , MSc; Faridi S. Jamaludin [f], Lotte Haverman [a] PhD; Willem B. de Vries [a], MD, PhD; Anne M. Eskes (AE) [g], RN, PhD; Jolanda M. Maaskant (JM) [a,b,h], RN, PhD

**Affiliations** [a]Amsterdam UMC, Emma Children's Hospital, Amsterdam, the Netherlands; [b]Amsterdam Reproduction & Development Research Institute, Amsterdam, the Netherlands; [c]Amsterdam Public Health, Amsterdam, the Netherlands; [d]Amsterdam UMC, Institute of Education and Training, Amsterdam, the Netherlands; [e]Erasmus Medical Centre, Sophia Children's Hospital, Department of Pediatric Intensive Rotterdam, the Netherlands; [f]Research support, Medical Library, Amsterdam UMC, University of Amsterdam, Amsterdam, the Netherlands; [g]Amsterdam UMC, Department of Surgery, Amsterdam, the Netherlands; [h]Amsterdam UMC, Department of Internal Medicine, Amsterdam, the Netherlands

**Corresponding author address:** Cor-Jan van der Perk, Emma Children's Hospital Amsterdam UMC, University of Amsterdam, Meibergdreef 9, 1105 AZ, Amsterdam the Netherlands, c.j.vanderperk@amsterdamumc.nl

**Online resource 5: References to Operationalizations**

| **Methods/ Tools** | **Patient category** |
|---|---|
| Practice or opinion based | CMC (1-43)<br>C(Y)SHCN (44-57)<br>CCC (58, 59)<br>PCC (60-64)<br>TD ((65, 66)<br>PCCI (67, 68)<br>LLC (69-71)<br>PMID/ PIMD (72) |
| CSHCN screener | C(Y)SHCN (73-89)<br>CMC (90-96)<br>PCC (97) |
| CCCCS | CCC (98-118)<br>CMC (119-125)<br>TD (126) |

| | |
|---|---|
| NSCH | CSHCN (127-136)<br>CMC (137-141)<br>PCC (142)<br>CCHC (143) |
| PMCA<br>PMCA + CCI + CCCS<br>PMCA + CWDA | CMC (144-152)<br>CMC (153)<br>CMC (154) |
| ICD | CMC (155)<br>LLC (156)<br>PCC (157, 158)<br>TD (159, 160) |
| SODCMC | CMC (161-165) |
| GMFCS | PMID/PIMD (166-168) |
| CRG + CCI | MCC (169, 170) |
| OTA | TD (171, 172) |
| ACCAPED | CMC (173) |
| AMGC | CMC (174) |
| Eligibility questionnaire | CMC (175) |
| BLSS | CSHCN (176) |
| Various operationalization methods in Systematic Review (CCCS, PMCA) | CMC (177) |

CSHCN: children with special healthcare needs, Complex Chronic Condition Classification System, NSCH: National Survey Children's Health, CCHC: Children with complex health conditions, PMCA: Pediatric Medical Complexity Algorithm, CCI Chronic Condition Indicator, CWDA: Children with Disabilities Algorithm, ICD: International Classification Of Diseases, MCC: Medically complex children, SODCMC: Standard Operational Definition for Children with Medical Complexity, GMFCS: Gross Motor Function Classification System, PMID: Profound and Multiple Intellectual Disabilities, CRG: Clinical Risk Groups, OTA: Office of Technology Assessment (OTA) criteria, ACCAPED: The Assessment of Complex Clinical Assistance Needs in Pediatrics, AMGC: Adjusted Morbidity Groups Classification System, BLSS: Bob's Level of Support Scale, CCC: Children with complex Chronic Conditions

1. Aryee E, Perrin JM, Clancy S, Merrill C, Curran M, Oreskovic NM. Mental Health of Caregivers of Children with Medical Complexity During COVID-19. Journal of developmental and behavioral pediatrics : JDBP. 2023;44(3):e212-e7.
2. Black L, Shaunfield S, Labellarte PH, Gaebler-Spira D, Foster CC. Physical and Environmental Barriers to Mobility and Participation in Children With Medical Complexity: A Qualitative Study. Clinical pediatrics. 2022;61(10):717-26.
3. Boss RD, Henderson CM, Weiss EM, Falck A, Madrigal V, Shapiro MC, et al. The Changing Landscape in Pediatric Hospitals: A Multicenter Study of How Pediatric Chronic Critical Illness Impacts NICU Throughput. American journal of perinatology. 2022;39(6):646-51.
4. Braun L, Steurer M, Henry D. Healthcare Utilization of Complex Chronically Ill Children Managed by a Telehealth-Based Team. Frontiers in pediatrics. 2021;9:689572.
5. Burrell M, Ciccarelli M. Identifying Children With Medical Complexity for Care Coordination in Primary Care Settings. Clinical pediatrics. 2022:99228221144803.
6. Caggiano S, Pavone M, Cherchi C, Paglietti MG, Schiavino A, Petreschi F, et al. Children with medical complexity and paediatric palliative care: data by a respiratory intermediate care unit. Pediatric pulmonology. 2022:918-26.
7. Coller RJ, Kelly MM, Howell KD, Warner G, Butteris SM, Ehlenbach ML, et al. In-Home COVID-19 Testing for Children With Medical Complexity: Feasibility and Association

With School Attendance and Safety Perceptions. American journal of public health. 2022;112:S878-S82.
8. Costain G, Walker S, Marano M, Veenma D, Snell M, Curtis M, et al. Genome Sequencing as a Diagnostic Test in Children With Unexplained Medical Complexity. JAMA Network Open. 2020;3(9):e2018109-e.
9. Dewan T, Birnie K, Drury J, Jordan I, Miller M, Neville A, et al. Experiences of medical traumatic stress in parents of children with medical complexity. Child: care, health and development. 2023;49(2):292-303.
10. Donnelly S, Shaw E, Timoney P, Foca M, Hametz P. Parents' Assessment of an Advanced-Practice Nurse and Care Coordination Assistant Model Medical Care Coordination Program for Children With Medical Complexity. Journal of pediatric health care : official publication of National Association of Pediatric Nurse Associates & Practitioners. 2020;34(4):325-32.
11. Foster CC, Fuentes MM, Wadlington LA, Jacob-Files E, Desai AD, Simon TD, et al. Caregiver and provider experiences of physical, occupational, and speech therapy for children with medical complexity. Journal of pediatric rehabilitation medicine. 2021;14(3):505-16.
12. Frankel H, Matiz LA, Friedman S. Siblings of Children with Medical Complexity-A Vulnerable Population in the Medical Home. Journal of health care for the poor and underserved. 2022;33(2):702-13.
13. Hamilton H, West AN, Ammar N, Chinthala L, Gunturkun F, Jones T, et al. Analyzing Relationships Between Economic and Neighborhood-Related Social Determinants of Health and Intensive Care Unit Length of Stay for Critically Ill Children With Medical Complexity Presenting With Severe Sepsis. Frontiers in public health. 2022;10:789999.
14. Hogan AK, Galligan MM, Stack NJ, Leach KF, Aredas BL, English R, et al. A Tertiary Care-based Complex Care Program: Improving Care for Children With Medical Complexity. Medical care. 2020;58(11):958-62.
15. Hsu NM, Morris K, Banaag A, Koehlmoos TP. TRICARE Extended Care Health Option Program: Prevalence of pediatric ECHO enrollees and healthcare service utilization in the Military Health System. Disability and health journal. 2023:101451.
16. Jacobs S, Davies N, Butterick KL, Oswell JL, Siapka K, Smith CH. Shared decision-making for children with medical complexity in community health services: a scoping review. BMJ paediatrics open. 2023;7(1):e001866.
17. Lawrence PR, Spratling R. A Theory for Understanding Parental Workload and Capacity to Care for Children With Medical Complexity. Research and theory for nursing practice. 2022;36(1):34-46.
18. Lin E, Scharbach K, Liu B, Braun M, Tannis C, Wilson K, et al. A Multidisciplinary Home Visiting Program for Children With Medical Complexity. Hospital pediatrics. 2020;10(11):925-31.
19. Lin JL, Huber B, Amir O, Gehrmann S, Ramirez KS, Ochoa KM, et al. Barriers and Facilitators to the Implementation of Family-Centered Technology in Complex Care: Feasibility Study. Journal of medical Internet research. 2022;24(8):e30902.
20. Mantler T, Jackson KT, Baer J, White J, Ache B, Shillington K, et al. Changes in Care-A Systematic Scoping Review of Transitions for Children with Medical Complexities. Current pediatric reviews. 2020;16(3):165-75.
21. Matsuzawa A, Shiroki Y, Arai J, Hirasawa A. Care coordination for children with medical complexity in Japan: Caregivers' perspectives. Child: care, health and development. 2020;46(4):436-44.
22. Murphy NA, Alvey J, Valentine KJ, Mann K, Wilkes J, Clark EB. Children With Medical Complexity: The 10-Year Experience of a Single Center. Hospital pediatrics. 2020;10(8):702-8.
23. Nassel D, Chartrand C, Dore-Bergeron M-J, Lefebvre F, Ballantyne M, Van Overmeire B, et al. Very Preterm Infants with Technological Dependence at Home: Impact on Resource Use and Family. Neonatology. 2019;115(4):363-70.

24. Nkoy F, Stone B, Hofmann M, Fassl B, Zhu A, Mahtta N, et al. Home-Monitoring Application for Children With Medical Complexity: A Feasibility Trial. Hospital pediatrics. 2021;11(5):492-502.
25. Notario PM, Gentile E, Amidon M, Angst D, Lefaiver C, Webster K. Home-Based Telemedicine for Children with Medical Complexity. Telemedicine journal and e-health : the official journal of the American Telemedicine Association. 2019;25(11):1123-32.
26. Parpia C, Moore C, Beatty M, Miranda S, Adams S, Stinson J, et al. Evaluation of a Secure Messaging System in the Care of Children With Medical Complexity: Mixed Methods Study. JMIR formative research. 2023;7:e42881.
27. Prieto V, Rozmus C, Cohen E, LoBiondo-Wood G. Caregiver Burden, Caregiving Satisfaction, and Health-Related Quality of Life Among Caregivers of Children with Medical Complexity. Pediatric Nursing. 2022;48(3):111-21.
28. Rojas CR, Moore A, Coffin A, McClam C, Ehritz C, Hogan A, et al. Medication Rounds: A Tool to Promote Medication Safety for Children with Medical Complexity. Joint Commission journal on quality and patient safety. 2023;49(4):226-34.
29. Sonsteng-Person M, Garcia-Perez J, Copeland V, Lievano-Karim L, Abrams D, Jarman B, et al. "What I Would Do to Take Away Your Pain": A Photovoice Project Conducted by Mothers of Children With Medical Complexity. Qualitative health research. 2023;33(3):204-19.
30. Teicher J, Moore C, Esser K, Weiser N, Arje D, Cohen E, et al. The Experience of Parental Caregiving for Children With Medical Complexity. Clinical pediatrics. 2022:99228221142102.
31. Thomson J, Butts B, Camara S, Rasnick E, Brokamp C, Heyd C, et al. Neighborhood Socioeconomic Deprivation and Health Care Utilization of Medically Complex Children. Pediatrics. 2022;149(4):1-9.
32. van der Perk C-J, van de Riet L, Alsem M, van Goudoever JB, Maaskant J. Prognostic factors influencing parental empowerment after discharge of their hospitalized child: A cross-sectional study. Journal of pediatric nursing. 2022;66:e145-e51.
33. Ware EJ, Beveridge MS, Rosado AI, Nageswaran S. Practical Needs in the Home Care of Latino Children With Medical Complexity. Home healthcare now. 2020;38(4):202-8.
34. Werner NE, Fleischman A, Warner G, Barton HJ, Kelly MM, Ehlenbach ML, et al. Feasibility Testing of Tubes@HOME: A Mobile Application to Support Family-Delivered Enteral Care. Hospital pediatrics. 2022;12(7):663-73.
35. Yamada H, Ohno K, Shiota M, Togawa M, Utsunomiya Y, Akaboshi S, et al. Prevalence and clinical characteristics of children with medical complexity in Tottori Prefecture, Japan: A population-based longitudinal study. Brain & development. 2020;42(10):747-55.
36. Yu J, Cook S, Imming C, Knezevich L, Ray K, Houtrow A, et al. A Qualitative Study of Family Caregiver Perceptions of High-Quality Care at a Pediatric Complex Care Center. Academic pediatrics. 2021:107-15.
37. Yu JA, Henderson C, Cook S, Ray K. Family Caregivers of Children With Medical Complexity: Health-Related Quality of Life and Experiences of Care Coordination. Academic pediatrics. 2020;20(8):1116-23.
38. Foster CC, Kwon S, Whitlow L, Cullen JP, Agrawal RK, Goodman D, et al. Connecting Hospital to Home: Characteristics of and Rehospitalization Rates in Hospitalized Children With Private-Duty Nursing. Hospital pediatrics. 2019;9(7):530-7.
39. Lim A, Butt ML, Dix J, Elliott L, Paes B. Respiratory syncytial virus (RSV) infection in children with medical complexity. European journal of clinical microbiology & infectious diseases : official publication of the European Society of Clinical Microbiology. 2019;38(1):171-6.
40. McKenzie K, Dudevich A, Costante A, Chen X-K, Foebel AD. How Children and Youth with Medical Complexity Use Hospital and Emergency Department Care across Canada. Healthcare quarterly (Toronto, Ont). 2021;24(1):10-3.

41. Nageswaran S, Banks Q, Golden SL, Gower WA, King NMP. The role of religion and spirituality in caregiver decision-making about tracheostomy for children with medical complexity. Journal of health care chaplaincy. 2022;28(1):95-107.
42. Nageswaran S, Gower WA, King NMP, Golden SL. Tracheostomy decision-making for children with medical complexity: What supports and resources do caregivers need? Palliative & supportive care. 2022:1-7.
43. Verduci E, Salvatore S, Bresesti I, Di Profio E, Pendezza E, Bosetti A, et al. Semi-Elemental and Elemental Formulas for Enteral Nutrition in Infants and Children with Medical Complexity-Thinking about Cow's Milk Allergy and Beyond. Nutrients. 2021;13(12):4230-.
44. Cosmo de Oliveira Carvalho C, Guilherme Pereira Pimentel T, Evangelista Cabral I. CHILD WITH SPECIAL HEALTH NEEDS AT ONE HOSPITAL OF THE BRAZILIAN UNIFIED HEALTH SYSTEM. Revista de Pesquisa: Cuidado e Fundamental. 2021;13(1):1296-302.
45. Costich MA, Peretz PJ, Davis JA, Stockwell MS, Matiz LA. Impact of a Community Health Worker Program to Support Caregivers of Children With Special Health Care Needs and Address Social Determinants of Health. Clinical pediatrics. 2019;58(11):1315-20.
46. da Silveira A, Santini Costenaro RG, Tatsch Neves E. ADOLESCENTS WITH SPECIAL HEALTH CARE NEEDS: CHALLENGES OF SCHOOL INCLUSION ACCORDING TO FAMILY MEMBERS/CAREGIVERS' VIEWPOINT. Revista de Pesquisa: Cuidado e Fundamental. 2020;12(1):1290-5.
47. Havinga J, Tumin D, Peedin L. Birth Weight Gradient in Parent-Reported Special Healthcare Needs among Children Born Preterm. The Journal of pediatrics. 2019:73-8.
48. Liu S, Lombardi J, Fisher PA. The COVID-19 Pandemic Impact on Households of Young Children With Special Healthcare Needs. Journal of pediatric psychology. 2022;47(2):158-70.
49. McKay S. Immigrant Children With Special Health Care Needs: A Review. Current problems in pediatric and adolescent health care. 2019;49(2):45-9.
50. Moretti A, Cianci P, De Paoli A, Meroni F, Taje S, Mariani M, et al. Burden of care in families of patients with rare genetic diseases: analysis of a large Italian cohort. European journal of medical genetics. 2021;64(7):104230.
51. Munambah N, Cordier R, Speyer R, Toto S, Ramugondo EL. A Systematic Review Comparing the Play Profiles of Children with Special Health Care Needs with Typically Developing Children. BioMed research international. 2020;2020:9582795.
52. O'Connor M, O'Connor E, Quach J, Vashishtha R, Goldfeld S. Trends in the prevalence and distribution of teacher-identified special health-care needs across three successive population cohorts. Journal of paediatrics and child health. 2019;55(3):312-9.
53. Pegorin TC, Furlan de Léo MM, Spiegelberg Zuge S, de Brum CN, da Rosa L, da Conceição VM. Quality of life and mental disorders in caregivers of children with special needs. Rev Rene. 2021;22(1):1-8.
54. Precce ML, Moraes JRMM, Pacheco STA, Silva LFD, Conceicao DSD, Rodrigues EDC. Educational demands of family members of children with special health care needs in the transition from hospital to home. Revista brasileira de enfermagem. 2020;73:e20190156.
55. Stille CJ, Coller RJ, Shelton C, Wells N, Desmarais A, Berry JG. National Research Agenda on Health Systems for Children and Youth With Special Health Care Needs. Academic pediatrics. 2022;22(2):S1-S6.
56. Wells R, Daniel P, Barger B, Rice CE, Bandlamudi M, Crimmins D. Impact of medical home-consistent care and child condition on select health, community, and family level outcomes among children with special health care needs. Children's Health Care. 2020:No-Specified.
57. Lillvis DF, Sheehan KM, Yu J, Noyes K, Bass KD, Kuo DZ. Characterizing physical trauma in children and youth with special health care needs. The journal of trauma and acute care surgery. 2022;93(3):299-306.
58. Fonseca R, Carvalho M, Querido A, Figueiredo MH, Bally J, Charepe Z. Therapeutic letters: A qualitative study exploring their influence on the hope of parents of children receiving pediatric palliative care in Portugal. Journal for specialists in pediatric nursing : JSPN. 2021;26(3):e12325.

59. Mattiello RMA, Pazin-Filho A, Aragon DC, Cupo P, Carlotti APCP. Impact of children with complex chronic conditions on costs in a tertiary referral hospital. Revista de saude publica. 2022;56:89.
60. Cobham VE, Hickling A, Kimball H, Thomas HJ, Scott JG, Middeldorp CM. Systematic review: Anxiety in children and adolescents with chronic medical conditions. Journal of the American Academy of Child & Adolescent Psychiatry. 2020;59(5):595-618.
61. da Silveira A, Chaves de Vargas TG, Portela de Oliveira J, Henrich Cazuni M, da Rosa B, de Vargas Bueno T, et al. NURSING CARE FOR CHILDREN AND ADOLESCENTS WITH SPECIAL HEALTH NEEDS. Ciencia, Cuidado e Saude. 2022;21:1-7.
62. Noonan K, Reichman NE, Corman H, Jimenez ME. School and Community Involvement of Adolescents With Chronic Health Conditions. The Journal of adolescent health : official publication of the Society for Adolescent Medicine. 2020;67(4):576-82.
63. Ullah F, Kaelber DC. Using Large Aggregated De-Identified Electronic Health Record Data to Determine the Prevalence of Common Chronic Diseases in Pediatric Patients Who Visited Primary Care Clinics. Acad Pediatr. 2021;21(6):1084-93.
64. Zylbersztejn A, Verfurden M, Hardelid P, Gilbert R, Wijlaars L. Phenotyping congenital anomalies in administrative hospital records. Paediatric and perinatal epidemiology. 2020;34(1):21-8.
65. Choi YH, Kim MS, Kim CH, Song IG, Park JD, In Suh D, et al. Looking into the life of technology-dependent children and their caregivers in Korea: lifting the burden of too many responsibilities. BMC pediatrics. 2020;20(1):486.
66. Matsuzawa A, Arai J, Shiroki Y, Hirasawa A. Healthcare for children depend on medical technology and parental quality of life in Japan. Pediatrics international : official journal of the Japan Pediatric Society. 2022;64(1):e15006.
67. Ruth AR, Boss RD, Donohue PK, Shapiro MC, Raisanen JC, Henderson CM. Living in the Hospital: The Vulnerability of Children with Chronic Critical Illness. The Journal of clinical ethics. 2020;31(4):340-52.
68. Shappley RKH, Noles DL, Spentzas T. Pediatric Chronic Critical Illness: Validation, Prevalence, and Impact in a Children's Hospital. Pediatric critical care medicine : a journal of the Society of Critical Care Medicine and the World Federation of Pediatric Intensive and Critical Care Societies. 2021;22(12):e636-e9.
69. Mitterer S, Zimmermann K, Bergstrasser E, Simon M, Gerber A-K, Fink G. Measuring Financial Burden in Families of Children Living With Life-Limiting Conditions: A Scoping Review of Cost Indicators and Outcome Measures. Value in health : the journal of the International Society for Pharmacoeconomics and Outcomes Research. 2021;24(9):1377-89.
70. Dunbar H, Carter B. Experiencing place identity and place belongingness at a children's hospice: Parents' perspectives. Journal of child health care : for professionals working with children in the hospital and community. 2020:1367493520915134.
71. Oakley S, Dunbar H, de Vries K. Parent-led strategies supporting personal well-being when caring for a child with a life-limiting condition: A scoping review. Journal of child health care : for professionals working with children in the hospital and community. 2022;26(4):648-67.
72. Luijkx J, van der Putten AAJ, Vlaskamp C. A valuable burden? The impact of children with profound intellectual and multiple disabilities on family life. Journal of Intellectual and Developmental Disability. 2019;44(2):184-9.
73. Matiz LA, Kostacos C, Robbins-Milne L, Chang SJ, Rausch JC, Tariq A. Integrating Nurse Care Managers in the Medical Home of Children with Special Health Care needs to Improve their Care Coordination and Impact Health Care Utilization. Journal of pediatric nursing. 2021;59:32-6.
74. Matiz LA, Robbins-Milne L, Rausch JA. EMR Adaptations to Support the Identification and Risk Stratification of Children with Special Health Care Needs in the Medical Home. Maternal and child health journal. 2019;23(7):919-24.
75. Pankewicz A, Davis RK, Kim J, Antonelli R, Rosenberg H, Berhane Z, et al. Children With Special Needs: Social Determinants of Health and Care Coordination. Clinical pediatrics. 2020;59(13):1161-8.

76. Vasan A, Kyle MA, Venkataramani AS, Kenyon CC, Fiks AG. Inequities in Time Spent Coordinating Care for Children and Youth with Special Health Care Needs. Academic pediatrics. 2023.
77. Arrue AM, Hokerberg YHM, Jantsch LB, da Gama SGN, de Oliveira RdVC, Okido ACC, et al. Prevalence of children with special healthcare needs: An epidemiological survey in Brazil. Journal of pediatric nursing. 2022;67:95-101.
78. Dembo RS, LaFleur J, Akobirshoev I, Dooley DP, Batra N, Mitra M. Racial/ethnic health disparities among children with special health care needs in Boston, Massachusetts. Disability and health journal. 2022;15(3):101316.
79. Geweniger A, Haddad A, Barth M, Hogl H, Mund A, Insan S, et al. Mental health of children with and without special healthcare needs and of their caregivers during COVID-19: a cross-sectional study. BMJ paediatrics open. 2022;6(1):e001509.
80. Ghandour RM, Hirai AH, Kenney MK. Children and Youth With Special Health Care Needs: A Profile. Pediatrics. 2022;149:S731-S47.
81. Johnels L, Vehmas S, Wilder J. Musical interaction with children and young people with severe or profound intellectual and multiple disabilities: a scoping review. International Journal of Developmental Disabilities. 2021:No-Specified.
82. Li M, Ji C, Wang B, Yao D, Wang X, Zeng Y, et al. Incomplete Vaccination Among Children With Special Health Care Needs in Zhejiang, China: Analysis of Retrospective Data. Frontiers in pediatrics. 2019;7:173.
83. Sonik RA, Coleman-Jensen A, Parish SL. Household food insufficiency, health status and emergency healthcare utilisation among children with and without special healthcare needs. Public Health Nutrition. 2020;23(17):3204-10.
84. Jenkins AM, Berry JG, Perrin JM, Kuhlthau K, Hall M, Dunbar P, et al. What Types of Hospitals Do Adolescents and Young Adults With Complex Chronic Conditions Use? Academic pediatrics. 2022;22(6):1033-40.
85. Lindly OJ, Martin AJ, Lally K. A Profile of Care Coordination, Missed School Days, and Unmet Needs Among Oregon Children with Special Health Care Needs with Behavioral and Mental Health Conditions. Community mental health journal. 2020;56(8):1571-80.
86. Moeenuddin Z, Kim-Kupfer C, Owchar E, Baker J, Duffield A, Santoro T. The Influence of Care Coordination on Patients With Special Health Care Needs in a Pediatric Residency Continuity Clinic. Global pediatric health. 2019;6:2333794X19848677.
87. Montes G. US children with special health care needs and ethnic discrimination: results from multivariate modeling. World journal of pediatrics : WJP. 2019;15(2):182-9.
88. Rose-Jacobs R, De Cuba SE, Bovell-Ammon A, Black MM, Coleman SM, Cutts D, et al. Housing instability among families with young children with special health care needs. Pediatrics. 2019;144(2):e20181704.
89. Evangelista Cabral I, Silva da Motta I, Pereira Pimentel TG, Pacheco de Oliveira Corrêa M, Moreira Arrué A, Neves ET. DEMANDAS DE CRIANÇAS COM NECESSIDADES ESPECIAIS DE SAÚDE NA ATENÇÃO PRIMÁRIA DA CIDADE DO RIO DE JANEIRO. Ciencia, Cuidado e Saude. 2020;19:1-10.
90. Lin JL, Clark CL, Halpern-Felsher B, Bennett PN, Assis-Hassid S, Amir O, et al. Parent Perspectives in Shared Decision-Making for Children With Medical Complexity. Academic pediatrics. 2020;20(8):1101-8.
91. Looman WS, Park YS, Gallagher TT, Weinfurter EV. Outcomes research on children with medical complexity: A scoping review of gaps and opportunities. Child: care, health and development. 2020;46(1):121-31.
92. Ming DY, Jackson GL, Sperling J, Gray M, Wyman Roth N, Spears T, et al. Mobile Complex Care Plans to Enhance Parental Engagement for Children With Medical Complexity. Clinical pediatrics. 2019;58(1):34-41.
93. Mooney-Doyle K, Lindley LC. The Association between Poverty and Family Financial Challenges of Caring for Medically Complex Children. Nursing Economic$. 2019;37(4):198-208.

94. Mooney-Doyle K, Lindley LC. Family and Child Characteristics Associated With Caregiver Challenges for Medically Complex Children. Family & community health. 2020;43(1):74-81.
95. Parente V, Parnell L, Childers J, Spears T, Jarrett V, Ming D. Point-of-Care Complexity Screening Algorithm to Identify Children With Medical Complexity. Hospital pediatrics. 2020:44-51.
96. Ross MH, Parnell LS, Spears TG, Ming DY. Telemedicine Video Visits for Children with Medical Complexity in a Structured Clinical Complex Care Program. Global pediatric health. 2020;7:2333794X20952196.
97. Barnard-Brak L, McGaha V, Little TD, Fearon-Drake D. Medical Home Outcomes for School-Aged Children With Chronic Health Care Needs: A Mokken Analysis. Quality management in health care. 2023;32(1):16-21.
98. Arias Lopez MDP, Fernandez AL, Fiquepron K, Meregalli C, Ratto ME, Siaba Serrate A. Prevalence of Children With Complex Chronic Conditions in PICUs of Argentina: A Prospective Multicenter Study. Pediatric critical care medicine : a journal of the Society of Critical Care Medicine and the World Federation of Pediatric Intensive and Critical Care Societies. 2019:E143-E51.
99. Bogetz JF, Revette A, DeCourcey D. Bereaved Parent Perspectives on the Benefits and Burdens of Technology Assistance among Children with Complex Chronic Conditions. Journal of palliative medicine. 2022;25(2):250-8.
100. Bogetz JF, Revette A, DeCourcey DD. Clinical Care Strategies That Support Parents of Children With Complex Chronic Conditions. Pediatric critical care medicine : a journal of the Society of Critical Care Medicine and the World Federation of Pediatric Intensive and Critical Care Societies. 2021;22(7):595-602.
101. Bogetz JF, Revette A, Rosenberg AR, DeCourcey D. "I Could Never Prepare for Something Like the Death of My Own Child": Parental Perspectives on Preparedness at End of Life for Children With Complex Chronic Conditions. Journal of pain and symptom management. 2020;60(6):1154-62.e1.
102. Fornehed MLC, Svynarenko R, Keim-Malpass J, Cozad MJ, Qualls KA, Stone WL, et al. Comparison between Rural and Urban Appalachian Children in Hospice Care. Southern medical journal. 2022;115(3):192-7.
103. Godoy-Molina E, Fernandez-Ferrandez T, Ruiz-Sanchez JM, Cordon-Martinez A, Perez-Frias J, Navas-Lopez VM, et al. A scale for the identification of the complex chronic pediatric patient (PedCom Scale): A pilot study. Anales de pediatria. 2022;97(3):155-60.
104. Lindley LC, Cohrs AC, Keim-Malpass J, Leslie DL. Children Enrolled in Hospice Care Under Commercial Insurance: A Comparison of Different Age Groups. The American journal of hospice & palliative care. 2019;36(2):123-9.
105. Parker CL, Wall B, Tumin D, Stanley R, Warren L, Deal K, et al. Care Coordination Program for Children With Complex Chronic Conditions Discharged From a Rural Tertiary-Care Academic Medical Center. Hospital pediatrics. 2020;10(8):687-93.
106. Bogetz JF, Revette A, Partin L, DeCourcey DD. Relationships and Resources Supporting Children With Serious Illness and Their Parents. Hospital pediatrics. 2022;12(9):832-42.
107. Berry JG, Difazio RL, Melvin P, Glader L, Casto E, Shore BJ. Hospital resource use after hip reconstruction surgery in children with neurological complex chronic conditions. Developmental medicine and child neurology. 2020:204-10.
108. Houtrow AJ, Carle A, Perrin JM, Stein REK. Children With Special Health Care Needs on Supplemental Security Income for Disability Have More Health Impacts and Needs Than Other Children With Special Health Care Needs on Medicaid. Academic pediatrics. 2020;20(2):258-66.
109. Lindley LC, Cozad MJ, Fortney CA. Pediatric Complex Chronic Conditions: Evaluating Two Versions of the Classification System. Western journal of nursing research. 2020;42(6):454-61.

110. Lindley LC, Fortney CA. Pediatric Complex Chronic Conditions: Does the Classification System Work for Infants? The American journal of hospice & palliative care. 2019;36(10):858-63.
111. Lindley LC, Fortney CA, Cozad MJ. Predictive Ability of an Illness Severity Measure: Implications for Nursing Research. Journal of nursing measurement. 2021;29(2):213-26.
112. Rupp Hanzen Andrades G, Abud Drumond Costa C, Crestani F, Tedesco Tonial C, Fiori H, Santos IS, et al. Association of nutritional status with clinical outcomes of critically ill pediatric patients with complex chronic conditions. Clinical nutrition (Edinburgh, Scotland). 2022;41(12):2786-91.
113. Hannan KE, Bourque SL, Palmer C, Tong S, Hwang SS. Prevalence and Predictors of Medical Complexity in a National Sample of VLBW Infants. Hospital pediatrics. 2021;11(5):525-35.
114. Deming RS, Wolfe J, DeCourcey DD. Weighing Distress and Benefit: Understanding the Research Participation Experiences of Bereaved Parents of Children with Complex Chronic Conditions. Journal of pain and symptom management. 2020:39-.
115. Dunbar PJ, Sobotka SA, Rodean J, Pulcini CD, Macy ML, Thomson J, et al. Prevalence of and Spending on Ear, Nose, Throat, and Respiratory Infections Among Children With Chronic Complex Conditions. Academic pediatrics. 2023;23(2):434-40.
116. Friedel M, Gilson A, Bouckenaere D, Brichard B, Fonteyne C, Wojcik T, et al. Access to paediatric palliative care in children and adolescents with complex chronic conditions: a retrospective hospital-based study in Brussels, Belgium. BMJ paediatrics open. 2019;3(1):e000547.
117. Lindley LC, Cozad MJ, Svynarenko R, Keim-Malpass J, Mack JW. A National Profile of Children Receiving Pediatric Concurrent Hospice Care, 2011 to 2013. Journal of hospice and palliative nursing : JHPN : the official journal of the Hospice and Palliative Nurses Association. 2021;23(3):214-20.
118. Pérez-Ardanaz B, José Peláez-Cantero M, Miguel Morales-Asencio J, Vellido-González C, Gómez-González A, León-Campos Á, et al. Socioeconomic Factors and Quality of Life Perceived by Parents and Children with Complex Chronic Conditions in Spain. Children. 2021;8(10):1-12.
119. Blaine K, Wright J, Pinkham A, O'Neill M, Wilkerson S, Rogers J, et al. Medication Order Errors at Hospital Admission Among Children With Medical Complexity. Journal of patient safety. 2022;18(1):e156-e62.
120. Jaaniste T, Cuganesan A, Wei Ling C, Tan SC, Coombs S, Heaton M, et al. Living with a child who has a life-limiting condition: The functioning of well-siblings and parents. Child: Care, Health & Development. 2022;48(2):269-76.
121. Peinado Fabregat MI, Saynina O, Sanders LM. Obesity and Overweight Among Children With Medical Complexity. Pediatrics. 2023;151(1).
122. Leary JC, Krcmar R, Yoon GH, Freund KM, LeClair AM. Parent Perspectives During Hospital Readmissions for Children With Medical Complexity: A Qualitative Study. Hospital pediatrics. 2020;10(3):222-9.
123. Marquez C, Thompson R, Feinstein JA, Orth LE. Identifying opportunities for pediatric medication therapy management in children with medical complexity. Journal of the American Pharmacists Association : JAPhA. 2022;62(5):1587-95.e3.
124. Vance AJ, Pan W, Malcolm WH, Brandon DH. Development of parenting self-efficacy in mothers of high-risk infants. Early human development. 2020;141:104946.
125. Leary JC, Price LL, Scott CER, Kent D, Wong JB, Freund KM. Developing Prediction Models for 30-Day Unplanned Readmission Among Children With Medical Complexity. Hospital pediatrics. 2019;9(3):201-8.
126. Rush M, Khan A, Barber J, Bloom M, Anspacher M, Fratantoni K, et al. Length of Stay and Barriers to Discharge for Technology-Dependent Children During the COVID-19 Pandemic. Hospital pediatrics. 2023;13(1):80-7.
127. Andersen JA, Morrow JE, Gibbs L, Hernandez NI. Caregiver reports of physician risk counseling for adolescents with special health care needs. Patient Education and Counseling. 2021:No-Specified.

128. Foster CC, Chorniy A, Kwon S, Kan K, Heard-Garris N, Davis MM. Children With Special Health Care Needs and Forgone Family Employment. Pediatrics. 2021;148(3):1-11.
129. Akobirshoev I, Parish S, Mitra M, Dembo R. Impact of Medical Home on Health Care of Children With and Without Special Health Care Needs: Update from the 2016 National Survey of Children's Health. Maternal and child health journal. 2019;23(11):1500-7.
130. Fuller AE, Brown NM, Grado L, Oyeku SO, Gross RS. Material Hardships and Health Care Utilization Among Low-Income Children with Special Health Care Needs. Academic pediatrics. 2019;19(7):733-9.
131. Gigli KH, Graaf G. Changes in Use and Access to Care for Children and Youth With Special Health Care Needs During the COVID-19 Pandemic. Journal of pediatric health care : official publication of National Association of Pediatric Nurse Associates & Practitioners. 2023;37(2):185-92.
132. Hagerman TK, McKernan GP, Carle AC, Yu JA, Stover AD, Houtrow AJ. The Mental and Physical Health of Mothers of Children with Special Health Care Needs in the United States. Maternal and child health journal. 2022;26(3):500-10.
133. Khanijahani A, Pawcio S. Household food insecurity and childhood obesity/overweight among children with special healthcare needs: Results from a nationally representative sample of 10-17 years old U.S. children. Pediatric obesity. 2023;18(5):e13015.
134. Sellmaier C. Physical and Mental Health of Mothers and Fathers Caring for Children with Special Health Care Needs: The Influence of Community Resources. Journal of Family Issues. 2022;43(11):2815-40.
135. Balistreri KS. Food insufficiency and children with special healthcare needs. Public health. 2019;167:55-61.
136. McLellan SE, Mann MY, Scott JA, Brown TW. A Blueprint for Change: Guiding Principles for a System of Services for Children and Youth With Special Health Care Needs and Their Families. Pediatrics. 2022;149:S722-S30.
137. Yu JA, Bayer ND, Beach SR, Kuo DZ, Houtrow AJ. A National Profile of Families and Caregivers of Children With Disabilities and/or Medical Complexity. Academic pediatrics. 2022;22(8):1489-98.
138. Yu JA, McKernan G, Hagerman T, Schenker Y, Houtrow A. Identifying children with medical complexity from the national survey of children s health combined 2016 17 data set. Hospital Pediatrics. 2021;11(2):192-7.
139. Bayer ND, Hongyue W, Yu JA, Kuo DZ, Halterman JS, Yue L. A National Mental Health Profile of Parents of Children With Medical Complexity. Pediatrics. 2021;148(2):1-10.
140. Jolliff A, Werner NE, Barton HJ, Howell KD, Kelly MM, Morgen M, et al. Caregiver perceptions of in-home COVID-19 testing for children with medical complexity: a qualitative study. BMC pediatrics. 2022;22(1):533.
141. Yu JA, McKernan G, Hagerman T, Schenker Y, Houtrow A. Most Children With Medical Complexity Do Not Receive Care in Well-Functioning Health Care Systems. Hospital pediatrics. 2021;11(2):183-91.
142. Rollins J, Rollins C, Boocks LA, Sitz T. Supporting children living with chronic medical conditions through empathetic art. Journal of Child and Family Studies. 2020;29(8):2218-33.
143. Azar R, Doucet S, Horsman AR, Charlton P, Luke A, Nagel DA, et al. A concept analysis of children with complex health conditions: implications for research and practice. BMC pediatrics. 2020;20(1):251.
144. Desai AD, Wang G, Wignall J, Kinard D, Singh V, Adams S, et al. User-centered design of a longitudinal care plan for children with medical complexity. Journal of the American Medical Informatics Association : JAMIA. 2020;27(12):1860-70.
145. Valdez RS, Lunsford C, Bae J, Letzkus LC, Keim-Malpass J. Self-Management Characterization for Families of Children With Medical Complexity and Their Social Networks: Protocol for a Qualitative Assessment. JMIR research protocols. 2020;9(1):e14810.
146. Gold JM, Chadwick W, Gustafson M, Valenzuela LF, Mello A, Nasr A. Parent perceptions and experiences regarding medication education at time of hospital discharge for children with medical complexity. Hospital Pediatrics. 2020;10(8):679-86.

147. Heneghan JA, Goodman DM, Ramgopal S. Variable Identification of Children With Medical Complexity in United States PICUs. Pediatric critical care medicine : a journal of the Society of Critical Care Medicine and the World Federation of Pediatric Intensive and Critical Care Societies. 2023;24(1):56-61.
148. Sadof M, Carlin S, Brandt S, Maypole J. A Step-by-Step Guide to Building a Complex Care Coordination Program in a Small Setting. Clinical pediatrics. 2019;58(8):897-902.
149. Wang G, Wignall J, Kinard D, Singh V, Foster C, Adams S, et al. An implementation model for managing cloud-based longitudinal care plans for children with medical complexity. Journal of the American Medical Informatics Association : JAMIA. 2021;28(1):23-32.
150. Ming DY, Jones KA, White MJ, Pritchard JE, Hammill BG, Bush C, et al. Healthcare Utilization for Medicaid-Insured Children with Medical Complexity: Differences by Sociodemographic Characteristics. Maternal and child health journal. 2022;26(12):2407-18.
151. Foster CC, Fuentes MM, Wadlington LA, Jacob-Files E, Desai AD, Simon TD, et al. Caregiver and Provider Experiences of Home Healthcare Quality for Children With Medical Complexity. Home healthcare now. 2020;38(3):138-46.
152. Xu WY, Li Y, Song C, Bose-Brill S, Retchin SM. Out-of-Network Care in Commercially Insured Pediatric Patients According to Medical Complexity. Med Care. 2022;60(5):375-80.
153. Blackmer AB, Fox D, Arendt D, Phillips K, Feinstein JA. Perceived Versus Demonstrated Understanding of the Complex Medications of Medically Complex Children. The journal of pediatric pharmacology and therapeutics : JPPT : the official journal of PPAG. 2021;26(1):62-72.
154. Leyenaar JK, Schaefer AP, Freyleue SD, Austin AM, Simon TD, Van Cleave J, et al. Prevalence of Children With Medical Complexity and Associations With Health Care Utilization and In-Hospital Mortality. JAMA pediatrics. 2022;176(6):e220687.
155. Moyes A, Abbott T, Baker S, Reid C, Thorne R, Morelius E. A parent first: Exploring the support needs of parents caring for a child with medical complexity in Australia. Journal of pediatric nursing. 2022;67:e48-e57.
156. Bowers AP, Chan RJ, Herbert A, Yates P. Estimating the prevalence of life-limiting conditions in Queensland for children and young people aged 0-21 years using health administration data. Australian health review : a publication of the Australian Hospital Association. 2020;44(4):630-6.
157. Luther AWM, Reaume SV, Qadeer RA, Thompson K, Ferro MA. Substance use disorders among youth with chronic physical illness. Addictive Behaviors. 2020;110:106517.
158. Silva N, Pereira M, Otto C, Ravens-Sieberer U, Canavarro MC, Bullinger M. Do 8- to 18-year-old children/adolescents with chronic physical health conditions have worse health-related quality of life than their healthy peers? a meta-analysis of studies using the KIDSCREEN questionnaires. Quality of life research : an international journal of quality of life aspects of treatment, care and rehabilitation. 2019;28(7):1725-50.
159. Giambra BK, Mangeot C, Benscoter DT, Britto MT. A Description of Children Dependent on Long Term Ventilation via Tracheostomy and Their Hospital Resource Use. Journal of pediatric nursing. 2021;61:96-101.
160. Berry JG, Goodman DM, Coller RJ, Agrawal R, Kuo DZ, Cohen E, et al. Association of Home Respiratory Equipment and Supply Use with Health Care Resource Utilization in Children. The Journal of pediatrics. 2019;207:169-75.e2.
161. Ann de Banate M, Maypole J, Sadof M. Care coordination for children with medical complexity. Current Opinion in Pediatrics. 2019;31(4):575-82.
162. Bergman DA, Keller D, Kuo DZ, Lerner C, Mansour M, Stille C, et al. Costs and Use for Children With Medical Complexity in a Care Management Program. Pediatrics. 2020;145(4):1-10.
163. Gallo M, Agostiniani R, Pintus R, Fanos V. The child with medical complexity. Italian journal of pediatrics. 2021;47(1):1.
164. Maypole J, Gavin T, de Banate MA, Sadof M. Lessons Learned, Best Practices: Care Coordination for Children with Medical Complexity. Pediatric annals. 2020;49(11):e457-e66.

165. Jewitt N, Orkin J, Cohen E, Narang I, Al-Saleh S, Amin R. The impact of polysomnograms and family-centred decision making in children with medical complexity. Paediatrics & child health. 2021;26(2):114-9.
166. Degache F, Bonjour A, Michaud D, Mondada L, Newman CJ. The effects of tandem skiing on posture and heart rate in children with profound intellectual and multiple disabilities. Developmental neurorehabilitation. 2019;22(4):234-9.
167. Dhondt A, Van Keer I, van der Putten A, Maes B. Communicative abilities in young children with a significant cognitive and motor developmental delay. Journal of applied research in intellectual disabilities : JARID. 2020;33(3):529-41.
168. Kruithof K, Olsman E, Nieuwenhuijse A, Willems D. Parents' views on medical decisions related to life and death for their ageing child with profound intellectual and multiple disabilities: A qualitative study. Research in developmental disabilities. 2022;121:104154.
169. Penela-Sanchez D, Ricart S, Vidiella N, Garcia-Garcia JJ. A study of paediatric patients with complex chronic conditions admitted to a paediatric department over a 12 month period. Estudio de los pacientes pediatricos cronicos complejos ingresados en un servicio de pediatria a lo largo de 12 meses. 2020.
170. Perros I, Papalexakis EE, Vuduc R, Searles E, Sun J. Temporal phenotyping of medically complex children via PARAFAC2 tensor factorization. Journal of biomedical informatics. 2019;93:103125.
171. Toly VB, Blanchette JE, Al-Shammari T, Musil CM. Caring for technology-dependent children at home: Problems and solutions identified by mothers. Appl Nurs Res. 2019;50:151195.
172. Toly VB, Zauszniewski JA, Yu J, Sattar A, Rusincovitch B, Musil CM. Resourcefulness Intervention Efficacy for Parent Caregivers of Technology-Dependent Children: A Randomized Trial. Western journal of nursing research. 2022;44(3):296-306.
173. Amarri S, Ottaviani A, Campagna A, De Panfilis L. Children with medical complexity and paediatric palliative care: a retrospective cross-sectional survey of prevalence and needs. Italian journal of pediatrics. 2021;47(1):110.
174. Carrilero N, Dalmau-Bueno A, Garcia-Altes A. Comorbidity patterns and socioeconomic inequalities in children under 15 with medical complexity: a population-based study. BMC pediatrics. 2020;20(1):358.
175. Buchanan F, Lai C, Cohen E, Milo-Manson G, Shachak A. Decision-making for Parents of Children With Medical Complexities: Activity Theory Analysis. Journal of participatory medicine. 2022;14(1):e31699.
176. Morton B, Damato EG, Ciccarelli MR, Currie J. Care Coordination for Children with Special Healthcare Needs Anticipating Transition: A Program Evaluation. Journal of pediatric nursing. 2021;61:7-14.
177. Sidra M, Sebastianski M, Ohinmaa A, Rahman S. Reported costs of children with medical complexity-A systematic review. Journal of child health care : for professionals working with children in the hospital and community. 2022:13674935221109683.
