# Supplementary material for: Definitions and operationalizations of pediatric chronic patients: a scoping review
Source: Eur J Pediatr. 2025 Nov 25;184(12):789. doi: 10.1007/s00431-025-06556-0 (PMC12644160; doi:10.1007/s00431-025-06556-0)
Supplement: Supplementary file 6 — Online resource 6 (PDF 129 KB) [file 431_2025_6556_MOESM6_ESM.pdf]

Definitions and operationalizations of pediatric chronic patients: A scoping review
European Journal of Pediatrics

Cor-Jan van der Perk (CJP) [a,b,c], MSc, RN; Lisa-Maria van Klaveren (LK) [c,d], MSc, MA; Karlijn S. Timmer (KT) [a,b,c], MSc, RN, Heleen N. Haspels [a,e] , MSc; Faridi S. Jamaludin [f], Lotte Haverman[a], PhD; Willem B. de Vries [a], MD, PhD; Anne M. Eskes (AE)[g], RN, PhD; Jolanda M. Maaskant (JM)[a,b,h], RN, PhD

Affiliations
[a] Amsterdam UMC, Emma Children's Hospital, Amsterdam, the Netherlands;
[b] Amsterdam Reproduction & Development Research Institute, Amsterdam, the Netherlands;
[c] Amsterdam Public Health, Amsterdam, the Netherlands;
[d] Amsterdam UMC, Institute of Education and Training, Amsterdam, the Netherlands;
[e] Erasmus Medical Centre, Sophia Children's Hospital, Department of Pediatric Intensive Rotterdam, the Netherlands;
[f] Research support, Medical Library, Amsterdam UMC, University of Amsterdam, Amsterdam, the Netherlands;
[g] Amsterdam UMC, Department of Surgery, Amsterdam, the Netherlands;
[h] Amsterdam UMC, Department of Internal Medicine, Amsterdam, the Netherlands

Corresponding author address:
Cor-Jan van der Perk
Emma Children's Hospital Amsterdam UMC, University of Amsterdam
Meibergdreef 9,
1105 AZ, Amsterdam
the Netherlands
c.j.vanderperk@amsterdamumc.nl

Online resource 6: Overview of tools used to define categories

	CSHCN screener	PMCA	CCI	NSCH
CMC	2	2		
CSHCN	12		1	1
PCC		1		

CMC: children with medical complexity; CSHCN: Children with special healthcare needs; PCC: Pediatric Chronic Conditions; PMCA: Pediatric medical Complexity Algorithm; CCI: Chronic Condition Indicator; NSCH:National Survey of Child Health
